# Robust axis elongation by Nodal-dependent restriction of BMP signaling

**DOI:** 10.1242/dev.202316

**Published:** 2024-02-19

**Authors:** Alexandra Schauer, Kornelija Pranjic-Ferscha, Robert Hauschild, Carl-Philipp Heisenberg

**Affiliations:** Institute of Science and Technology Austria, Klosterneuburg 3400, Austria

**Keywords:** Axial elongation, BMP signaling, Gastrulation, Zebrafish

## Abstract

Embryogenesis results from the coordinated activities of different signaling pathways controlling cell fate specification and morphogenesis. In vertebrate gastrulation, both Nodal and BMP signaling play key roles in germ layer specification and morphogenesis, yet their interplay to coordinate embryo patterning with morphogenesis is still insufficiently understood. Here, we took a reductionist approach using zebrafish embryonic explants to study the coordination of Nodal and BMP signaling for embryo patterning and morphogenesis. We show that Nodal signaling triggers explant elongation by inducing mesendodermal progenitors but also suppressing BMP signaling activity at the site of mesendoderm induction. Consistent with this, ectopic BMP signaling in the mesendoderm blocks cell alignment and oriented mesendoderm intercalations, key processes during explant elongation. Translating these *ex vivo* observations to the intact embryo showed that, similar to explants, Nodal signaling suppresses the effect of BMP signaling on cell intercalations in the dorsal domain, thus allowing robust embryonic axis elongation. These findings suggest a dual function of Nodal signaling in embryonic axis elongation by both inducing mesendoderm and suppressing BMP effects in the dorsal portion of the mesendoderm.

## Teaser

**Summary:** Nodal signaling renders embryonic axis elongation in zebrafish blastoderm explants and intact embryos robust by suppressing BMP activity to allow effective cell intercalations.

## INTRODUCTION

Embryonic development relies on cell fate specification and tissue shape changes mediated by conserved signaling pathways. These pathways often display dual functions, instructing embryo patterning and the morphogenetic capacity of cells (Heisenberg and Solnica-Krezel, 2008; Rogers and Schier, 2011; Briscoe and Small, 2015; Gilmour et al., 2017; Sagner and Briscoe, 2017; Valet et al., 2022; Bailles et al., 2022). In line with this, cell fate specification is tightly linked with the emergence of distinct cellular behaviors, such as cell migration and intercalation, collectively leading to large-scale tissue reorganization (Keller et al., 2003; Solnica-Krezel, 2005; Solnica-Krezel and Sepich, 2012; Gilmour et al., 2017; Hannezo and Heisenberg, 2019; Collinet and Lecuit, 2021; Valet et al., 2022). Thus, to pattern and shape the embryo robustly and reproducibly, fate specification and morphogenesis have to be closely spatiotemporally coordinated.

A hallmark of vertebrate gastrulation, the first major morphogenetic process in embryogenesis leading to germ layer formation (ectoderm, mesoderm and endoderm), is the elongation of the embryonic body along its anterior-posterior (AP) axis, involving large-scale cell rearrangements and cell fate specification (Solnica-Krezel and Sepich, 2012). During zebrafish gastrulation, AP body axis elongation is driven by highly conserved convergence and extension (C&E) movements (Tada and Heisenberg, 2012; Williams and Solnica-Krezel, 2020a). These C&E movements comprise cells undergoing medially oriented intercalations (mediolateral intercalations and planar-medial intercalations; Yin et al., 2008; Glickman et al., 2003) in dorsal positions of the gastrula and collective cell migration anteriorly and ventrolaterally, the combination of which is supposed to drive body axis elongation (Tada and Heisenberg, 2012; Williams and Solnica-Krezel, 2020a). Concomitant with axis elongation, the tissues undergoing C&E movements become patterned along their AP and dorsal-ventral (DV) axes by the graded activity of the conserved transforming growth factor β signals Nodal and bone morphogenetic protein (BMP), key factors regulating both axial patterning and morphogenesis during zebrafish gastrulation (Myers et al., 2002b; Schier and Talbot, 2005; Heisenberg and Solnica-Krezel, 2008; Zinski et al., 2018; Pinheiro and Heisenberg, 2020; Williams and Solnica-Krezel, 2020a; Hill, 2022; Pinheiro et al., 2022). Although the processes setting up the graded activity domains of these signals, termed ‘morphogens’, and their capacity to control fate specification and cell behavior have been studied for some time (Myers et al., 2002b; Schier and Talbot, 2005; Heisenberg and Solnica-Krezel, 2008; Zinski et al., 2018; Rogers and Müller, 2019; Pinheiro and Heisenberg, 2020; Williams and Solnica-Krezel, 2020a; Hill, 2022; Pinheiro et al., 2022), much less is known about how their activities are spatiotemporally coordinated.

Seminal work in *Xenopus* has demonstrated that cultured gastrula explants containing the dorsal blastopore lip or activin-treated prospective animal ectoderm can undergo medio-lateral cell intercalations leading to axis elongation (Keller et al., 1985, 1992; Symes and Smith, 1987; Keller and Danilchik, 1988; Sokol and Melton, 1991; Wilson and Keller, 1991; Shih and Keller, 1992a,b; Green et al., 2004; Ninomiya et al., 2004). Interestingly, *Xenopus* explant elongation depends on proper mesendodermal AP patterning (Ninomiya et al., 2004), and the specific combination of factors inducing mesendoderm (Green et al., 1990; Cunliffe and Smith, 1992; Howard and Smith, 1993; Graff et al., 1994), suggesting a close link between signaling, patterning and axis elongation also *in vitro*. More recently, it has been shown that mammalian embryonic stem cell-derived 3D *in vitro* models of gastrulation (gastruloids) undergo elongation (van den Brink et al., 2014; Turner et al., 2017; Beccari et al., 2018; Moris et al., 2020; Anlaş et al., 2021c preprint; Xu et al., 2021), and that, similar to *Xenopus* explants, this elongation depends on the specific morphogen signaling regime (van den Brink et al., 2014; Turner et al., 2017; Anlaş et al., 2021 preprint; Underhill and Toettcher, 2023; Hennessy et al., 2023 preprint). This suggests that also in mammalian gastrulation models, the appropriate spatiotemporal organization of morphogen signaling is a key determinant for axis elongation.

Here, we have used zebrafish embryonic explants to investigate how the spatial organization of morphogen signaling domains affects axis elongation. We found that mesendoderm elongation in explants is predominantly driven by oriented cell intercalations, which can only occur when BMP signaling levels are sufficiently low within the mesendoderm. Nodal signaling, in addition to its role in triggering mesendoderm induction, maintains such low BMP signaling levels at the site of explant elongation by suppressing BMP activity, a crucial function also required in the intact embryo for robust body axis elongation.

## RESULTS

### Mesendoderm elongation during gastrulation is driven by cell intercalation behavior in explants

**Fig. 1.**
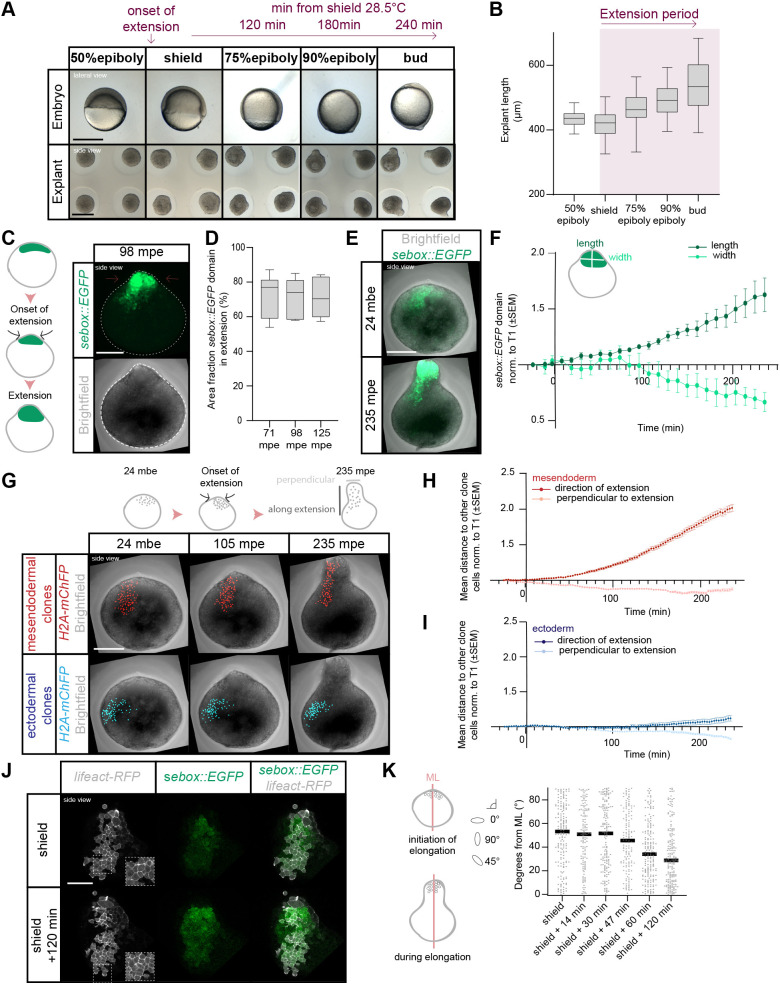
**Mesendoderm morphogenesis and cell dispersal during explant elongation.** (A) Single-plane brightfield images of embryos (lateral view) and explants (side-view) at consecutive stages from 50% epiboly to bud stage. Timeline at the top indicates the explant extension onset and the time between the indicated developmental stages (28.5°C). (B) Explant length at consecutive stages from 50% epiboly to bud stage (*n*=45, *N*=3). (C) Maximum intensity projection of fluorescence (top) and brightfield (bottom) images (side-views) of explants obtained from Tg(*sebox::EGFP*) embryos at 98 min post explant elongation onset (mpe). (D) Area fraction of EGFP expression (mesendoderm) within the extension of explants obtained from Tg(*sebox::EGFP*) embryos at 71, 98 and 125 mpe (*n*=7, *N*=7). (E) Maximum intensity projection of fluorescence/brightfield images (side-views) of explants from Tg(*sebox::EGFP*) embryos 24 min before the onset of explant elongation (mbe; top) and at 235 mpe (bottom). (F) EGFP expression domain length/width ratio in blastoderm explants from Tg(*sebox::EGFP*) embryos during explant elongation (*n*=6, *N*=6). Length is calculated in the direction of mesendoderm elongation and width oriented perpendicular to the length. (G) Maximum intensity projections of fluorescence/brightfield images (side-views) of blastoderm explants before elongation (24 mbe; left), during elongation (105 mpe; middle) and at the end (235 mpe; right) showing clonally labeled nuclei in the mesendoderm (red) or ectoderm (blue). Images shown for clones at 24 mbe and 235 mpe correspond to the explant as shown for the mesendodermal progenitor domain in E. Note that wild-type explant images for mesendodermal clones correspond to those in Fig. 3A. (H,I) Mesendodermal (H) and ectodermal (I) clone dispersal parallel and perpendicular to the explant elongation axis assessed as mean distance of each tracked cell to other clone cells during elongation (mesendoderm >320 cells analyzed per time point, *n*=5, *N*=5; ectoderm >240 cells analyzed per time point, *n*=5, *N*=5). (J) Single-plane high-resolution images of explants (side-views) obtained from Tg(*sebox::EGFP*) embryos at the onset of explant elongation (corresponding to embryonic shield stage) and during elongation (shield+120 min). Cell outlines are marked by *lifeact-RFP* (gray). (K) Cell alignment assessed by deviation (degrees) of the main cell extension axis from the main mediolateral (ML) explant axis at the onset of explant elongation (shield: 168 cells, *n*=5, *N*=5) and during elongation (shield+14 min: 132 cells; shield+30 min: 162 cells; shield+47 min: 144 cells; shield+60 min: 184 cells; shield+120 min: 188 cells; *n*=5, *N*=5). Scale bars: 500 µm (A); 200 µm (C,E,G); 100 µm (J).

Body axis elongation in zebrafish embryos during gastrulation is the result of the concerted action of various cell behaviors characteristic for different germ layer identities induced by morphogen gradients (Myers et al., 2002b; Heisenberg and Solnica-Krezel, 2008; Tada and Heisenberg, 2012; Zinski et al., 2018; Williams and Solnica-Krezel, 2020a). Zebrafish embryonic explants, similar to other *in vitro* gastrulation models (Anlaş and Trivedi, 2021; Steventon et al., 2021; Emig and Williams, 2022), undergo elongation reminiscent of axis extension in intact embryos upon local Nodal signaling activation (Fig. 1A,B, Fig. S1A), with the extension being composed largely of mesendodermal progenitors (Xu et al., 2014; Fulton et al., 2020; Williams and Solnica-Krezel, 2020b; Schauer et al., 2020; Cheng et al., 2023). However, the underlying cellular rearrangements and contributions of germ layer progenitor types to this process are not yet fully understood. To address this, we first analyzed how progenitors change their position throughout the elongation movement. In the initial phase of explant elongation, the extended part of the explant was predominantly composed of mesendodermal cells, identified by the expression of the pan-mesendodermal marker *sebox* (Poulain and Lepage, 2002; Ruprecht et al., 2015) (Fig. 1C,D, Fig. S1B,C). Moreover, during explant elongation the mesendodermal tissue within the extension changed its shape by elongating along the explant elongation axis and narrowing perpendicular to it (Fig. 1E,F, Fig. S1D, Movie 1), while keeping its area largely constant (Fig. S1E), consistent with suggestions that whole explant elongation is driven by C&E movements rather than oriented growth (Xu et al., 2014; Williams and Solnica-Krezel, 2020b; Fulton et al., 2020; Schauer et al., 2020; Cheng et al., 2023). Analysis of the displacement of the ectoderm and mesendoderm boundaries during the elongation process showed that the mesendoderm undergoes more extensive and earlier elongation than ectoderm (Fig. S1F), suggesting that mesendoderm morphogenesis constitutes an important driving force for explant elongation. In line with this, lowering Nodal signaling activity and concomitantly the amount of mesendoderm in the explants reduces the extent of explant elongation (Fig. S1G-H′), further supporting a link between mesendoderm C&E movements and overall explant elongation.

To gain insight into the cell rearrangements occurring within the mesendoderm, we tracked the movement and analyzed the dispersal of *sebox::EGFP*-positive cells over time by generating cell clones of varying sizes and position within the mesendoderm expressing the nuclear marker *H2A-mChFP* (Fig. 1G,H). During explant elongation, the mean distance between clonal cells increased along the elongation axis and decreased perpendicular to it (2.02-fold change in distance in direction of and 0.88-fold change in distance perpendicular to extension over 260 min) (Fig. 1G,H, Fig. S1I,I′, Movies 2-4). The overall organization of the mesendoderm was largely preserved, as evidenced by the relative distances of mesendodermal progenitors to the explant tip at the onset versus the end of the extension process being correlated (R^2^=0.6748, Fig. S1J). To determine whether this cell rearrangement pattern was the result of medially oriented mesendodermal cell intercalations, similar to dorsal tissues of the embryo (Glickman et al., 2003; Yin et al., 2008), we analyzed the orientation of the longest cell axis relative to the explant mediolateral axis during elongation (Fig. 1J,K) (Williams and Solnica-Krezel, 2020b). At the onset of explant elongation (corresponding to embryonic shield stage), the cells appeared randomly oriented (Fig. 1J,K, Fig. S1K, Movies 5,6). However, during the subsequent elongation process, the longest cell axis became increasingly aligned with the mediolateral explant axis (Fig. 1J,K, Fig. S1K, Movies 5,6), consistent with the notion that these cells were undergoing coordinated cell alignment, a hallmark for medially oriented cell intercalations (Glickman et al., 2003).

Interestingly, extending our cell dispersal analysis to the ectoderm showed that ectodermal progenitors initially exhibited only limited clone elongation along the explant elongation axis (0.99-fold change in distance over 122 min compared with 1.2-fold change for mesendodermal progenitors), but that ectodermal clonal elongation became more pronounced at late stages of the process (1.12-fold change in distance in direction of and 0.83-fold change in distance perpendicular over 260 min) (Fig. 1G,I, Fig. S1I,I′, Movies 7-9). This suggests that the coordinated activity of mesendodermal and ectodermal C&E movements might be required to reach full explant elongation.

### BMP-mediated dorsoventral patterning determines the elongation capacity of explants

**Fig. 2.**
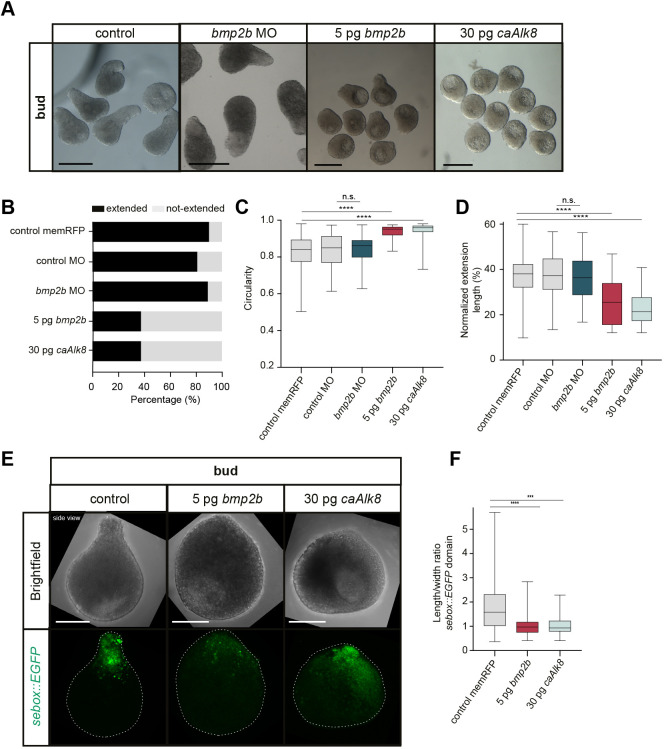
**Changes in explant elongation upon BMP signaling overactivation.** (A) Single-plane brightfield images of explants from wild-type embryos (control: *n*=191, *N*=11), and embryos injected with 1.5 ng *bmp2b* morpholino (MO) (*n*=55, *N*=5), 5 pg *bmp2b* (*n*=56, *N*=5) or 30 pg *caAlk8* mRNA (*n*=112, *N*=6) (side-views) at bud stage. Control explants correspond to explants shown in Fig. S2D. (B) Percentage of extended/not-extended explants from wild-type embryos (control: *n*=191, *N*=11; 5 ng control MO: *n*=63, *N*=5), and embryos injected with 1.5 ng *bmp2b* MO (*n*=55, *N*=5), 5 pg *bmp2b* (*n*=56, *N*=5) or 30 pg *caAlk8* mRNA (*n*=112, *N*=6) at bud stage. (C) Circularity of extended/not-extended explants from wild-type embryos (control: *n*=191, *N*=11; control MO: *n*=63, *N*=5), and embryos injected with 1.5 ng *bmp2b* MO (*n*=55, *N*=5), 5 pg *bmp2b* (*n*=56, *N*=5) or 30 pg *caAlk8* mRNA (*n*=112, *N*=6) at bud stage. ****P<0.0001 (Kruskal-Wallis test); ns, not significant (unpaired *t*-test). (D) Normalized extension length of explants from wild-type embryos (control: *n*=172, *N*=11; control MO: *n*=51, *N*=5), and embryos injected with 1.5 ng *bmp2b* MO (*n*=49, *N*=5), 5 pg *bmp2b* (*n*=21, *N*=5) or 30 pg *caAlk8* mRNA (*n*=45, *N*=6) at bud stage. ****P<0.0001 (one-way ANOVA); ns, not significant (unpaired *t*-test). Embryos in A-D were co-injected with 50-100 pg *memRFP* or *memGFP* as injection control. (E) Maximum intensity projections of brightfield/fluorescence images (side-views) of explants from Tg(*sebox::EGFP*) wild-type embryos (control: *n*=62, *N*=8), and Tg(*sebox::EGFP*) embryos injected with 5 pg *bmp2b* (*n*=33, *N*=6) or 30 pg *caAlk8* mRNA (*n*=31, *N*=4) at bud stage. Control explants correspond to explants shown in Fig. S3A. (F) Length/width ratio of the EGFP expression domain in explants from Tg(*sebox::EGFP*) wild-type embryos (control: *n*=62, *N*=8), and Tg(*sebox::EGFP*) embryos injected with 5 pg *bmp2b* (*n*=33, *N*=6) or 30 pg *caAlk8* mRNA (*n*=31, *N*=4) at bud stage. Embryos in E,F were co-injected with 50-100 pg *memRFP* as injection control. ****P<0.0001, ***P=0.0002 (Kruskal–Wallis test). Scale bars: 500 µm (A); 200 µm (E).

Given that mesendodermal cell rearrangements are important for explant elongation and that different mesendodermal fates display distinct morphogenetic capacities in zebrafish embryos (Tada and Heisenberg, 2012; Williams and Solnica-Krezel, 2020a), we assessed the role of mesendodermal patterning for explant elongation. Taking advantage of the inherent variability in the induction of mesodermal cell fates in explants (Schauer et al., 2020; Fulton et al., 2020), we first analyzed the relationship between explant elongation/length and expression domain size of dorsal [*hgg* (*ctslb*) (Thisse et al., 1994), *flh* (*noto*) (Talbot et al., 1995)], paraxial [*papc* (*pcdh8*); Yamamoto et al., 1998), ventrolateral (*tbx16*; Griffin et al., 1998) and ventral (*drl*; Herbomel et al., 1999) mesodermal markers at the end of gastrulation, corresponding to embryonic bud stage (Fig. S2A-C). Although there was no clear correlation between expression domain size of the tested markers and explant length (Fig. S2A), we noted that the most ventral mesodermal fate markers tested were rarely expressed (Fig. S2B,C). To test whether induction of these most ventral mesodermal fates might interfere with explant elongation, we analyzed the effect of changing BMP signaling levels, a key determinant for zebrafish DV patterning (Zinski et al., 2018), on explant elongation by treating embryos with various positive and negative BMP regulators. We found that reducing BMP signaling by morpholino-mediated *bmp2b* knockdown (Lele et al., 2001a) or overexpression of the BMP antagonist Chordin (*chrd-GFP*; Hammerschmidt et al., 1996; Pomreinke et al., 2017) affected neither the frequency of explant elongation nor the average extension length (Fig. 2A-D, Fig. S2D-G). In contrast, increasing BMP signaling and thereby ventral mesodermal cell fates by overexpressing *bmp2b* or *caAlk8* (constitutively active BMP receptor; Bauer et al., 2001) (Fig. S2B,C) severely reduced the explant elongation frequency (Fig. 2A-C, Fig. S2D-F) and the average extension length (Fig. 2A,D, Fig. S2D,G). This reduction in explant elongation frequency was accompanied by a reduced length/width ratio of the mesendoderm (Fig. 2E,F, Fig. S3A,B) and depended on the expression of the BMP downstream signaling mediator *smad5* (Fig. S3C-F) (von der Hardt et al., 2007). Local BMP signaling activation by *caAlk8* overexpression in large cell clones led to the most severe elongation defects, when these clones were located within the mesendoderm (Fig. S3G-I), further supporting that overactivation, but not abrogation, of BMP signaling affects explant elongation.

**Fig. 3.**
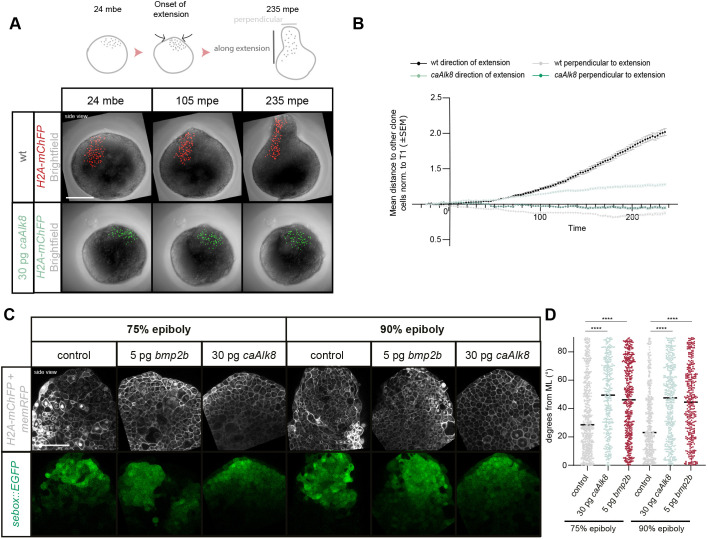
**Changes in clone elongation and cell alignment in explants upon BMP signaling overactivation.** (A) Maximum intensity projections of fluorescence/brightfield images (side-views) of explants before elongation (24 mbe; left), during elongation (105 mpe; middle) and at the end (235 mpe; right) showing clonally labeled mesendodermal cell nuclei in explants from wild-type (wt) embryos (red) or embryos injected with 30 pg *caAlk8* mRNA (green). Note that wild-type explant images correspond to those in Fig. 1G. (B) Mesendodermal clone dispersal parallel and perpendicular to the elongation axis assessed as mean distance of each cell to other clone cells during elongation in explants from wild-type embryos (black/gray) or embryos injected with 30 pg *caAlk8* mRNA (light/dark green) (wild type >320 cells analyzed per time point, *n*=5, *N*=5; 30 pg *caAlk8* >420 cells analyzed per time point, *n*=5, *N*=5). Note that the wild-type explant data corresponds to that shown in Fig. 1H. (C) Single-plane high-resolution images (side-views) of explants obtained from Tg(*sebox::EGFP*) embryos, and Tg(*sebox::EGFP*) embryos injected with 5 pg *bmp2b* and 30 pg *caAlk8* during explant elongation (corresponding to embryonic 75% and 90% epiboly). Cell outlines are marked by *memRFP* (gray), and nuclei by *H2A-mChFP* (gray) mRNA injection. (D) Cell alignment assessed by the deviation (degrees) of the main cell extension axis from the main mediolateral (ML) explant axis during elongation (75% epiboly: wild type: 545 cells, *n*=12, *N*=6; 5 pg *bmp2b*: 417 cells, *n*=11, *N*=6; 30 pg *caAlk8*: 342 cells, *n*=11, *N*=6; 90% epiboly: wild type: 439 cells, *n*=12, *N*=7; 5 pg *bmp2b*: 347 cells, *n*=11, *N*=7; 30 pg *caAlk8*: 399 cells, *n*=11, *N*=7). ****P<0.0001 (Kruskal–Wallis test). Scale bars: 200 µm (A); 100 µm (C).

### BMP signaling reduces mesendodermal cell dispersal and cell alignment

Next, we examined how increased BMP signaling within the mesendoderm reduces explant elongation. Given that BMP signaling has been suggested to spatially restrict oriented cell intercalations to the dorsal side in the zebrafish gastrula (Myers et al., 2002a), we analyzed whether overactivation of BMP in the mesendoderm might reduce explant elongation by inhibiting cell intercalation. Comparing mesendodermal clones in control explants to *caAlk8* overexpressing explants with elevated BMP signaling revealed that clone elongation along the direction of the extension was reduced upon BMP overactivation (2.02-fold mean increase in clone length in wild type versus 1.28-fold increase in *caAlk8* explants) (Fig. 3A,B, Fig. S4A, Movie 10). In line with this, cells in *caAlk8*-overexpressing explants failed to effectively polarize and align along the mediolateral explant axis during elongation (Fig. 3C,D), indicative of reduced medially oriented intercalations. Other functions of BMP signaling in mesendoderm morphogenesis, such as regulating the direction of mesendoderm migration during convergence (von der Hardt et al., 2007), were unlikely to represent main effector processes by which excessive BMP signaling interferes with explant elongation, as decreasing BMP signaling activity (Fig. 2A-D, Fig. S2D-G) or *has2* knockdown (Fig. S4B-D), a crucial convergence movement regulator in embryos (Bakkers et al., 2004), had no clear effect on explant elongation.

Collectively, these observations suggest that BMP-induced mesendoderm ventralization blocks explant elongation by reducing medially oriented mesendodermal cell alignment and intercalation.

### BMP signaling is attenuated in the mesendoderm by Nodal signaling activity

**Fig. 4.**
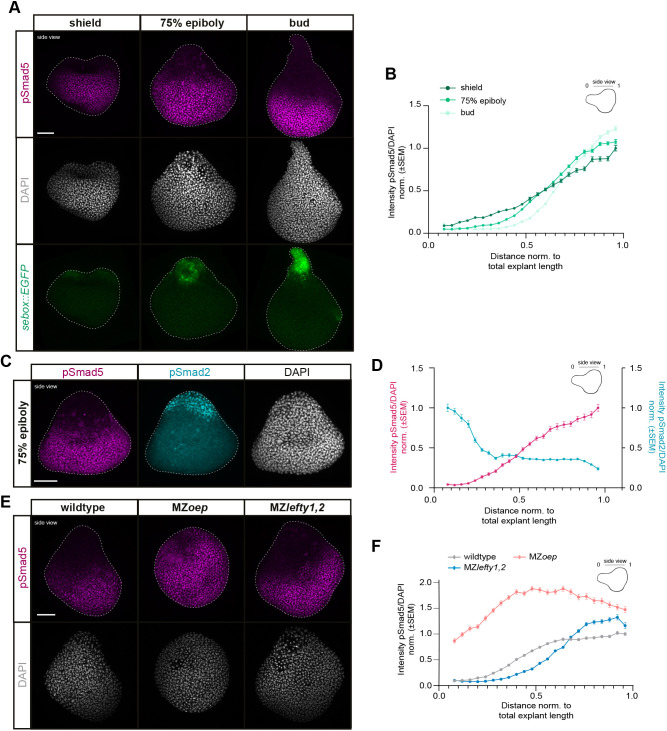
**Repression of BMP signaling activity by Nodal signaling at the sites of mesendoderm induction and explant elongation.** (A) Maximum intensity projections of fluorescence images (side-views) of explants from Tg(*sebox::EGFP*) embryos during explant elongation (corresponding to embryonic shield: *n*=6, *N*=3; 75% epiboly: *n*=6, *N*=3; bud stage: *n*=6, *N*=3) stained for pSmad5 (magenta) and DAPI (gray). (B) Intensity of nuclear pSmad5 normalized to DAPI as function of the distance from the explant tip during explant elongation (corresponding to embryonic shield stage: *n*=6, *N*=3; 75% epiboly: *n*=6, *N*=3; bud stage: *n*=6, *N*=3). Intensities are shown relative to the mean intensity in the bin closest to the back (high-intensity domain) of shield-stage explants; 4% of the explant at the sample edges were excluded due to low number of nuclei. (C) Maximum intensity projections of fluorescence images (side-views) of explants during elongation (corresponding to embryonic 75% epiboly) stained for pSmad5 (magenta), pSmad2 (cyan) and DAPI (gray) (*n*=6, *N*=3). (D) Intensity of nuclear pSmad5 (magenta) and pSmad2 (cyan) normalized to DAPI as function of the distance from the explant tip during explant elongation (corresponding to embryonic 75% epiboly stage) (*n*=6, *N*=3). Intensities are shown relative to the mean intensity in the bin closest to the explant back (high-intensity domain) for pSmad5 and closest to the explant tip for pSmad2 (high-intensity domain); 4% of the explant at the sample edges were excluded due to low number of nuclei. (E) Maximum intensity projections of fluorescence images (side-views) of explants from wild-type, MZ*oep* and MZ*lefty1,2* embryos during elongation (corresponding to embryonic 75% epiboly) stained for pSmad5 (magenta) and DAPI (gray) (wild type: *n*=9, *N*=4; MZ*oep*: *n*=9, *N*=4; MZ*lefty1,2*: *n*=9, *N*=4). (F) Intensity of nuclear pSmad5 normalized to DAPI as function of the distance from the explant tip during elongation (corresponding to embryonic 75% epiboly stage) (wild type: *n*=9, *N*=4; MZ*oep*: *n*=9, *N*=4; MZ*lefty1,2*: *n*=9, *N*=4). Intensities are shown relative to the mean intensity in the first bin closest to the back (high-intensity domain) of wild-type explants; 4% of the explant at the sample edges were excluded due to low number of nuclei. Scale bars: 100 µm (A,C,E).

Given that spatial confinement of BMP signaling activity is important for explant elongation, we next investigated how BMP signaling is distributed within the explants. In line with previous observations (Fulton et al., 2020; Schauer et al., 2020), we found a long-range gradient of BMP signaling, monitored by nuclear localization of the phosphorylated, activated form of the BMP signaling mediator Smad5 (pSmad5), extending across the explant during the elongation phase with low pSmad5 levels overlapping with the site of elongation (Fig. 4A,B). Interestingly, when we analyzed the pSmad5 activity domain in a *sebox::EGFP* background, we noted that the mesendodermal *sebox::EGFP*-positive domain was largely devoid of pSmad5, suggesting that BMP signaling was inherently suppressed in the explant mesendoderm (Fig. 4A,B). This pSmad5 restriction appeared to be important for proper mesendodermal cell alignment as increased pSmad5 levels upon overexpression of 1 pg *bmp2b* led to a loss of proper cell alignment (Fig. S4E-G′).

As mesendoderm induction depends on Nodal signaling (Schier, 2009; Rogers and Müller, 2019; Economou and Hill, 2020; Hill, 2022), we explored whether Nodal signaling negatively regulates the extent of BMP signaling concomitantly with its function in inducing mesendoderm, a potential function consistent with observations in embryos showing that the expression of several components of the BMP signaling pathway is affected by changes in Nodal signaling activity (Gritsman et al., 1999; Sirotkin et al., 2000; Lele et al., 2001b; Bennett et al., 2007; Varga et al., 2007; Xu et al., 2014; Wang et al., 2021 preprint; Cheng et al., 2023). We found that the activities of pSmad5 and the Nodal signaling effector pSmad2 peaked in opposing domains during explant elongation (Fig. 4C,D). Moreover, in explants prepared from mutant embryos with increased Nodal signaling activity (MZ*lefty1,2*) (Rogers et al., 2017) (Fig. S5A,B), or devoid of active Nodal signaling (MZ*oep*) (Gritsman et al., 1999) (Fig. S5A,B), the extent of the domain of high nuclear pSmad5 was reduced (MZ*lefty1,2*) or clearly expanded (MZ*oep*) along the back-tip explant axis (Fig. 4E,F, Fig. S5C-G). This suggests that Nodal signaling represses BMP signaling within the mesendoderm. Given that neither the expansion of the Nodal signaling domain in MZ*lefty1,2* explants (Fig. S5A,B), nor the extent of the pSmad5 domain in wild-type and/or MZ*lefty1,2* explants directly translated into changes in explant length (Fig. S5H,I), this activity of Nodal signaling in restricting BMP signaling appears to be due to a signaling function of Nodal, changing also the amount of mesendoderm (Fig. S5J), rather than being the secondary consequence of Nodal-induced morphogenesis, previously shown to separate BMP and Wnt signaling domains for neuroectoderm patterning (Fulton et al., 2020). To test when Nodal signaling is required for restricting the BMP signaling domain, we treated explants with the Nodal inhibitor SB-505124 (Byfield et al., 2004; Rogers et al., 2017) from the time point of explant preparation (256-cell stage) until the onset of gastrulation. This strongly expanded the pSmad5 domain along the explant axis (Fig. S5K,L), suggesting that Nodal signaling needs to be activated prior to the onset of gastrulation to effectively restrict BMP signaling. In line with this, inhibiting Nodal signaling only after the onset of gastrulation did not change the pSmad5 domain extent (Fig. S5K,L).

### Nodal represses BMP signaling activity during explant elongation by *chrd* upregulation

Next, we investigated how Nodal signaling couples mesendoderm induction with BMP signaling restriction, two prerequisites for explant elongation (Xu et al., 2014; Cheng et al., 2023; Fulton et al., 2020; Schauer et al., 2020; Williams and Solnica-Krezel, 2020b) (Fig. S1G-H′, Fig. 2). Ubiquitous overexpression of the Nodal ligand *cyclops* (*ndr2*), resulting in embryos consisting of only mesendoderm, blocked BMP signaling activity as monitored by the lack of nuclear pSmad5 (Fig. S6A,B). This could be rescued by co-injecting *caAlk8* (Fig. S6A,B), suggesting that Nodal signaling antagonizes BMP signaling activity predominantly by regulating extracellular or surface-bound BMP signaling effectors.

**Fig. 5.**
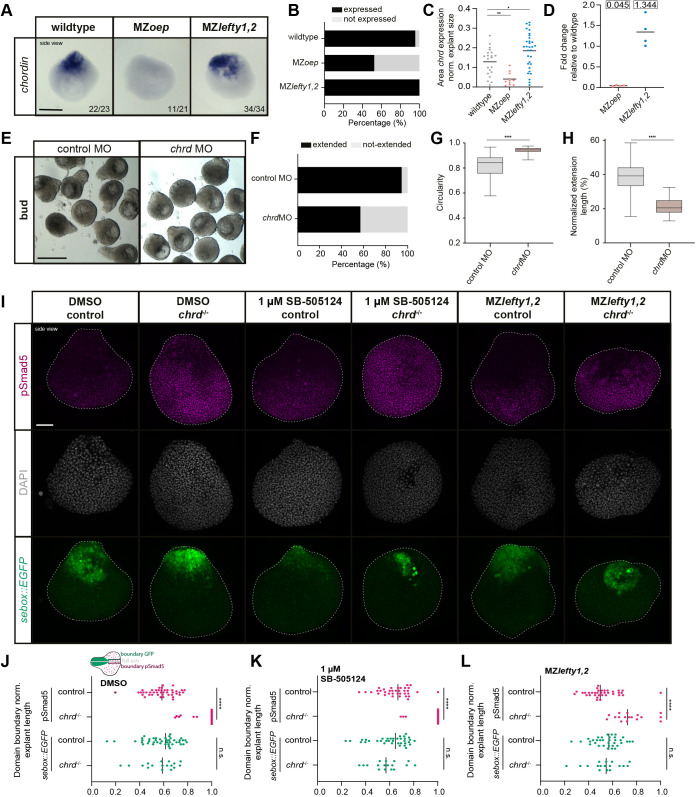
**Restriction of BMP signaling activity and explant elongation by Nodal-dependent *chordin* regulation.** (A) Expression of *chordin* (*chrd*) assessed by *in situ* hybridization in explants from wild-type, MZ*oep* and MZ*lefty1,2* embryos (side-view) during elongation (corresponding to embryonic 75% epiboly). Proportion of explants with a similar expression pattern to the image is shown in the lower corner (wild type: *n*=23, *N*=3; MZ*oep*: *n*=21, *N*=3; MZ*lefty1,2*: *n*=34, *N*=3). (B) Percentage of explants expressing/not-expressing *chrd* (wild type: *n*=23, *N*=3; MZ*oep*: *n*=21, *N*=3; MZ*lefty1,2*: *n*=34, *N*=3). (C) *chrd* expression area assessed by *in situ* hybridization normalized to explant area during elongation (corresponding to embryonic 75% epiboly) (wild type: *n*=21, *N*=3; MZ*oep*: *n*=11, *N*=3; MZ*lefty1,2*: *n*=31, *N*=3). Black line indicates the mean. *P=0.0229, **P=0.0061 (one-way ANOVA). (D) Fold-change of *chrd* expression in explants from MZ*oep* (*N*=4) and MZ*lefty1,2* (*N*=4) embryos relative to explants from wild-type embryos during elongation (corresponding to embryonic 75% epiboly stage). Black line indicates the mean. (E) Single-plane brightfield images (side-views) of explants obtained from wild-type embryos (control MO: *n*=74, *N*=5) and embryos injected with 4.5 ng *chrd* MO (*n*=56, *N*=5) at bud stage. (F) Percentage of extended/not-extended explants from wild-type embryos (control MO: *n*=74, *N*=5) and embryos injected with 4.5 ng *chrd* MO (*n*=56, *N*=5) at bud stage. (G) Circularity of extended/not-extended explants from wild-type embryos (control MO: *n*=74, *N*=5) and embryos injected with 4.5 ng *chrd* MO (*n*=56, *N*=5) at bud stage. ****P<0.0001 (Mann–Whitney test). (H) Normalized extension length of elongated explants from wild-type embryos (control MO: *n*=70, *N*=5) and embryos injected with 4.5 ng *chrd* MO (*n*=28, *N*=5) at bud stage. ****P<0.0001 (unpaired *t*-test). Embryos in E-H were co-injected with 50-100 pg *memRFP* or *memGFP* as injection control. (I) Maximum intensity projections of fluorescence images (side-views) of explants from wild-type embryos and explants from *chrd*^−/−^ mutant embryos treated with DMSO [treated from 256c to 75% epiboly (256c→75% epiboly); wild type: *n*=43, *N*=6; *chrd*^−/−^: *n*=17, *N*=4] or 1 µM SB-505124 (256c→75% epiboly; wild type: *n*=36, *N*=6; *chrd*^−/−^: *n*=17, *N*=6) and explants from MZ*lefty1,2* (*n*=35, *N*=5) and MZ*lefty1,2*;*chrd*^−/−^ (*n*=17, *N*=4) mutant embryos with Tg(*sebox::EGFP*) marking mesendoderm progenitors (green) during elongation (corresponding to embryonic 75% epiboly stage) stained for pSmad5 (magenta) and DAPI (gray). (J-L) Domain of nuclear pSmad5 and EGFP expression measured along a line from the explant back through its center to the domain start normalized to explant length in explants obtained from wild-type embryos and from *chrd*^−/−^ embryos treated with DMSO (256c→75% epiboly; wild type: *n*=43, *N*=6; *chrd*^−/−^: *n*=17, *N*=4) (J), 1 µM SB-505124 (256c→75% epiboly; wild type: *n*=36, *N*=6; *chrd*^−/−^: *n*=17, *N*=6) (K) and from MZ*lefty1,2* (*n*=35, *N*=5) and MZ*lefty1,2;chrd*^−/−^ (*n*=17, *N*=4) mutant embryos (L) during elongation (corresponding to embryonic 75% epiboly) with Tg(*sebox::EGFP*) marking mesendoderm progenitors. Vertical lines indicate the median. ****P<0.0001; ns, not significant (Mann–Whitney test: *sebox::EGFP* domain extent in J and pSmad5 domain extent in K,L; unpaired *t*-test: pSmad domain extent in J and *sebox::EGFP* domain extent in K,L). Scale bars: 200 µm (A); 500 µm (E); 100 µm (I).

The expression domain of a main BMP antagonist, the dorsally expressed *chrd* (Hammerschmidt et al., 1996; Schulte-Merker et al., 1997; Miller-Bertoglio et al., 1997), has been shown to be modulated by Nodal signaling (Gritsman et al., 1999; Sirotkin et al., 2000; Bennett et al., 2007; Varga et al., 2007; Xu et al., 2014; Cheng et al., 2023). Hence, we sought to assess a potential requirement of Nodal-dependent *chrd* activation for restricting BMP signaling at the site of explant elongation. For this, we first analyzed changes in *chrd* expression in explants obtained from MZ*lefty1,2* (Rogers et al., 2017) and MZ*oep* (Gritsman et al., 1999) mutant embryos during explant elongation (corresponding to embryonic 75% epiboly). Whereas increasing Nodal signaling above wild-type levels in MZ*lefty1,2* explants led to mildly elevated *chrd* expression (Fig. 5A-D; note the variability of *chrd* expression in MZ*lefty1,2* explants), reduced Nodal signaling in MZ*oep* mutant explants strongly diminished *chrd* expression as shown by *in situ* hybridization and qPCR (Fig. 5A-D). This suggests that Nodal signaling activation is required for properly establishing the *chrd* expression domain within explants.

To assess whether *chrd* is required for explant elongation by restricting BMP signaling activity downstream of Nodal*,* we first analyzed how *chrd* loss of function affects explant elongation by generating blastoderm explants from *chrd* morphant embryos (Nasevicius and Ekker, 2000). *chrd* morphant explants showed a reduction in explant elongation frequency and length (Fig. 5E-H) and a reduced length/width ratio of the mesendoderm (Fig. S6C,D), suggesting that proper *chrd* function is required for allowing effective explant elongation. To test whether *chrd* functions in this process downstream of Nodal, we analyzed the ability of Nodal signaling to modulate pSmad5 in explants obtained from *chrd* mutant embryos (Hammerschmidt et al., 1996). To this end, we prepared explants from wild-type and *chrd* mutant embryos (Hammerschmidt et al., 1996) with normal Nodal signaling (DMSO), reduced Nodal signaling (1 µM SB-505124; Byfield et al., 2004), leading to an expansion of the pSmad5 domain compared with controls (Fig. S6E,F), or increased Nodal signaling [MZ*lefty1,2* (Rogers et al., 2017) or MZ*lefty1,2*;*chrd*^−/−^ triple mutant explants] (Fig. 5I-L). Whereas the relative positioning of the *sebox::EGFP* expression domain along the main explant axis seemed largely unaffected in *chrd*^−/−^ explants (Fig. 5I-L), the pSmad5 activity domain was strongly expanded into the mesendoderm, and this effect occurred irrespectively of Nodal signaling levels (Fig. 5I-L, Fig. S6G,H). Notably, an area devoid of nuclear pSmad5 was still formed in some *chrd*^−/−^ explants, in particular when Nodal signaling levels were increased (MZ*lefty1,2;chrd*^−/−^ explants) (Fig. 5I-L, Fig. S6G,H), suggesting that additional *chrd*-independent mechanisms might be activated by peak Nodal signaling levels to further repress BMP signaling.

A candidate for mediating the *chrd*-independent function of high Nodal signaling in repressing BMP signaling is *noggin 1* (*nog1*), previously shown to be expressed in a Nodal-sensitive manner and displaying some functional redundancy with *chrd* in DV patterning (Fürthauer et al., 1999; Sirotkin et al., 2000; Dal-Pra et al., 2006; Ramel and Hill, 2013). Interestingly, although qPCR analysis showed that *nog1* expression was upregulated in MZ*lefty1,2* explants compared with wild-type explants at 75% epiboly (Fig. S7A), *nog1* knockdown by two different morpholinos (Dal-Pra et al., 2006) had no major effect on the pSmad5 domain extent (Fig. S7B,C) or explant elongation (Fig. S7D-G). To test whether *nog1* expression is responsible for the limited restriction of pSmad5 in wild-type and MZ*lefty1,2* mutant explants without *chrd*, we analyzed how the pSmad5 domain extent changed along the back-tip axis in explants from control *chrd*^−/−^ mutant (4 ng control MO/embryo) and *nog1*MO;*chrd*^−/−^ double morphant/mutant embryos (Hammerschmidt et al., 1996; Dal-Pra et al., 2006) with normal or increased Nodal signaling (MZ*lefty1,2* mutant) (Fig. S7H-J). This showed that the remaining restriction of pSmad5 in *chrd* mutant explants was lost in *nog1*;*chrd* double morphant/mutants, even when Nodal signaling was elevated (MZ*lefty1,2*; Fig. S7H-J), suggesting that *nog1* and *chrd* display a partially redundant function in restricting BMP signaling from the explant mesendoderm downstream of Nodal signaling.

### Nodal signaling maintains an area of low BMP signaling on the dorsal side of the gastrula for robust axis elongation

**Fig. 6.**
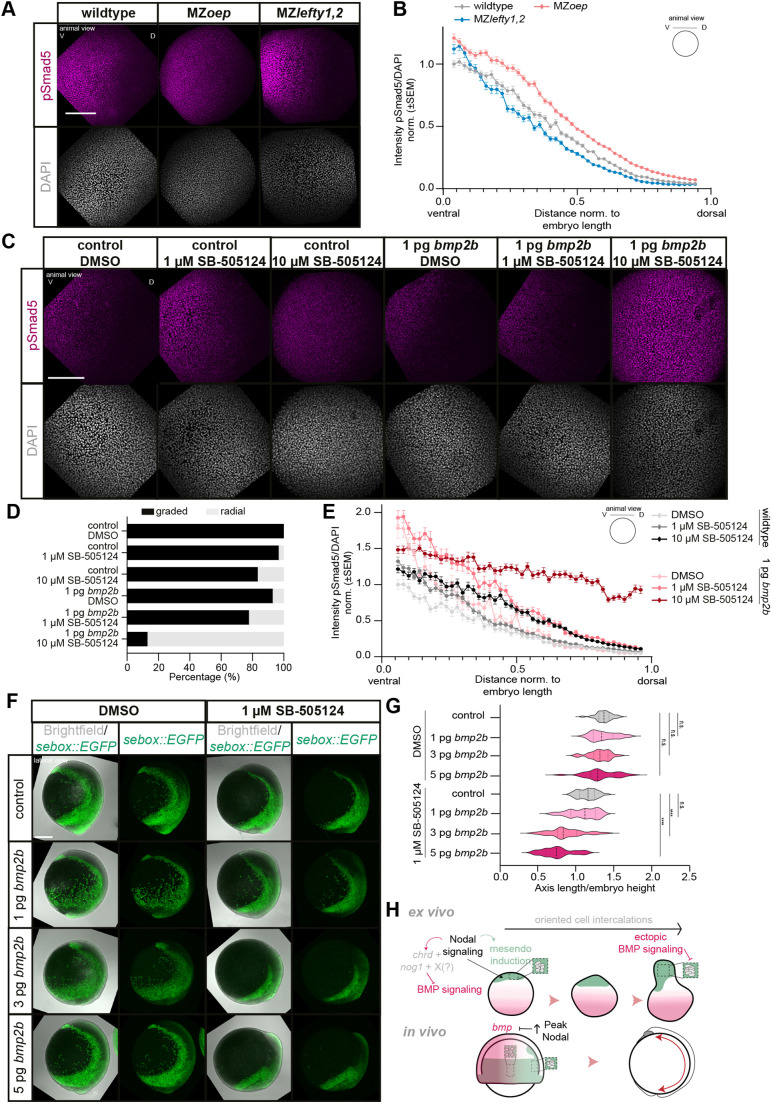
**Suppression of BMP activity dorsally by peak Nodal signaling provides robustness for body axis elongation.** (A) Maximum intensity projections of fluorescence images (animal views) of wild-type, MZ*oep* and MZ*lefty1,2* embryos at 75% epiboly stained for pSmad5 (magenta) and DAPI (gray) (wild type: *n*=11, *N*=5; MZ*oep*: *n*=11, *N*=5; MZ*lefty1,2*: *n*=11, *N*=5). (B) Intensity of nuclear pSmad5 normalized to DAPI as a function of the distance from the ventral side at 75% epiboly stage (wild type: *n*=11, *N*=5; MZ*oep*: *n*=11, *N*=5; MZ*lefty1,2*: *n*=11, *N*=5). Intensities are shown relative to the mean intensity in the bin closest to the ventral side (high-intensity domain) of wild-type embryos. 4% of the embryo at the sample edges were excluded due to low number of nuclei. (C) Maximum intensity projections of fluorescence images (animal views) of DMSO-treated [treated from 4-16c to 75% epiboly (4-16c→75% epiboly); control: *n*=35, *N*=6; 1 pg *bmp2b*: *n*=28, *N*=6], 1 µM SB-505124-treated (4-16c→75% epiboly; control: *n*=30, *N*=6; 1 pg *bmp2b*: *n*=27, *N*=6) and 10 µM SB-505124-treated (4-16c→75% epiboly; control: *n*=36, *N*=6; 1 pg *bmp2b*: *n*=23, *N*=6) embryos at 75% epiboly stage stained for pSmad5 (magenta) and DAPI (gray). (D) Proportion of DMSO-treated (4-16c→75% epiboly: control: *n*=35, *N*=6; 1 pg *bmp2b*: *n*=28, *N*=6), 1 µM SB-505124-treated (4-16c→75% epiboly: control: *n*=30, *N*=6; 1 pg *bmp2b*: *n*=27, *N*=6) and 10 µM SB-505124-treated (4-16c→75% epiboly; control: *n*=36, *N*=6; 1 pg *bmp2b*: *n*=23, *N*=6) embryos at 75% epiboly exhibiting graded nuclear pSmad5 or a radial pSmad5 domain. (E) Intensity of nuclear pSmad5 normalized to DAPI as a function of the distance from the ventral side of 75% epiboly DMSO-treated (4-16c→75% epiboly; control: *n*=7, *N*=3; 1 pg *bmp2b*: *n*=7, *N*=3), 1 µM SB-505124-treated (4-16c→75% epiboly; control: *n*=7, *N*=3; 1 pg *bmp2b*: *n*=7, *N*=3) and 10 µM SB-505124-treated (4-16c→75% epiboly; control: *n*=7, *N*=3; 1 pg *bmp2b*: *n*=7, *N*=3) embryos. Intensities are shown relative to the mean intensity in the bin closest to the ventral side (high-intensity domain) of wild-type embryos; 4% of the embryo at the sample edges were excluded owing to a low number of nuclei in these regions. Embryos in C-E were co-injected with 80 pg *memGFP* as injection control. (F) Maximum intensity projections of brightfield/fluorescence images (lateral views) of DMSO-treated [treated from 4-16c to bud stage (4-16c→bud stage); control: *n*=30, *N*=7; 1 pg *bmp2b*: *n*=28, *N*=7; 3 pg *bmp2b*: *n*=22, *N*=7; 5 pg *bmp2b*: *n*=25, *N*=7] (left) or 1 µM SB-505124-treated (4-16c→bud stage; control: *n*=40, *N*=7; 1 pg *bmp2b*: *n*=31, *N*=7; 3 pg *bmp2b*: *n*=28, *N*=7; 5 pg *bmp2b*: *n*=28, *N*=7) (right) embryos at bud stage expressing Tg(*sebox::EGFP*) (green). (G) Ratio of mesendodermal AP axis length/embryo height (the overall size of the embryo) of DMSO-treated (4-16c→bud stage; control: *n*=30, *N*=7; 1 pg *bmp2b*: *n*=28, *N*=7; 3 pg *bmp2b*: *n*=22, *N*=7; 5 pg *bmp2b*: *n*=25, *N*=7) or 1 µM SB-505124-treated (4-16c→bud stage; control: *n*=40, *N*=7; 1 pg *bmp2b*: *n*=31, *N*=7; 3 pg *bmp2b*: *n*=28, *N*=7; 5 pg *bmp2b*: *n*=28, *N*=7) embryos at bud stage. Embryos in F,G were co-injected with 80 pg *memRFP* as injection control. ****P<0.0001; ns, not significant (one-way ANOVA). (H) Schematic of Nodal-dependent restriction of BMP signaling activity (magenta) from the mesendoderm (green) that permits effective axial elongation by cell intercalations *ex vivo* and *in vivo* by upregulation of *chrd* and *nog1* expression, and other potential contributing factors. Full arrowheads indicate positive regulation of genes and cell fates. Straight lines indicate negative regulation of signaling activities and processes. Scale bar: 200 µm (A,C,F). D, dorsal; V, ventral.

Given the key role of Nodal signaling in mediating explant elongation by inducing mesendoderm and restricting BMP activity, we examined whether a similar function exists in embryos. In contrast to opposing pSmad2 and pSmad5 gradients in explants, BMP and Nodal signaling partially overlap and have partially mutually exclusive activity domains in the intact embryo, with pSmad2 being activated in the embryonic margin and dorsal axis at 75% epiboly, and pSmad5 being activated along the animal-vegetal axis in ventral/lateral domains (Fig. S8A) (reviewed by Schier and Talbot, 2005; Rogers and Müller, 2019; Hill, 2022). Like in explants and consistent with reports in embryos (Gritsman et al., 1999; Sirotkin et al., 2000; Bennett et al., 2007; Varga et al., 2007), *chrd* expression was strongly reduced in MZ*oep* mutants and *chrd* and *nog1* expression were upregulated in MZ*lefty1,2* mutants at 75% epiboly (Fig. S8B). These changes in *chrd* and *nog1* expression were accompanied by changes in the long-range pSmad5 gradient, extending from ventral-to-dorsal in embryos (Ramel and Hill, 2013; Tucker et al., 2008; Zinski et al., 2017; Pomreinke et al., 2017; Rogers et al., 2020; Greenfeld et al., 2021), with the gradient being expanded in MZ*oep* mutants (Gritsman et al., 1999) and reduced in MZ*lefty1,2* mutants (Rogers et al., 2017) at 75% epiboly (Fig. 6A,B, Fig. S8C-F‴). Specifically, we found that at 75% epiboly MZ*oep* mutants showed an overall increase and expansion of the domain of high nuclear pSmad5 levels compared with wild-type embryos (Fig. 6A,B, Fig. S8C,D,F-F‴), whereas the domain of high pSmad5 signaling nuclei was more restricted towards the ventral side in MZ*lefty1,2* mutants (Fig. 6A,B, Fig. S8C,E-F‴). Nodal signaling was required during early development to properly shape the pSmad5 gradient, as treatments with SB-505124 from the 4- to 16-cell stage until shield stage were sufficient to expand the pSmad5 gradient at 75% epiboly, whereas the pSmad5 gradient remained largely unaffected when embryos were treated from shield stage until 75% epiboly (Fig. S8G-L).

To further challenge the hypothesis that Nodal signaling maintains an area of low BMP signaling at the dorsal side of embryos, we analyzed changes in dorsal nuclear pSmad5 levels upon *bmp2b* overexpression in the presence of normal and reduced Nodal signaling. To this end, we injected low amounts of *bmp2b* mRNA, which on its own would not abolish pSmad5 gradient formation (Fig. 6C-E, Fig. S9A,D), in embryos exposed to 1 or 10 µM SB-505124 (Byfield et al., 2004). We found that with increasing SB-505124 concentration, embryos became more sensitive to *bmp2b* overexpression, leading to embryos eventually displaying near-uniform high pSmad5 upon simultaneous BMP overexpression and Nodal signaling reduction (Fig. 6C-E, Fig. S9A-G‴). This showed that Nodal signaling at the dorsal side of the embryo is required to repress BMP signaling effectively.

Importantly, the impact of reduced Nodal signaling on the pSmad5 gradient was less pronounced in the embryos compared with explants (Fig. S9H-I), as evidenced by a more pronounced increase of pSmad5 levels and further spread of peak pSmad5 nuclei in explants compared with embryos with reduced Nodal signaling (MZ*oep* or treatment with 1 µM SB-505124) (Fig. S9H-I). In line with this, *chrd* expression also appeared more strongly downregulated in MZ*oep* explants compared with MZ*oep* embryos (Fig. 5D, Fig. S8B). Collectively, this suggests that Nodal signaling is required in both embryos and explants to effectively create an area of low BMP signaling activity at the site of axial elongation. However, the phenotypic penetrance depends on the overall embryonic context, indicative of additional partially redundant factors, such as extra-embryonic signals, also restricting pSmad5 in the embryo.

When analyzing the effect of BMP overactivation on axis elongation in embryos by overexpressing *caAlk8*, we found that the AP length of the mesendoderm was reduced at bud stage (Fig. S10A,B), consistent with previous findings (Myers et al., 2002a). Furthermore, we observed that cell alignment along the mediolateral axis within the dorsal mesendoderm was disrupted upon BMP overactivation at 90% epiboly (Fig. S10C,D), suggesting that, similar to the situation in explants (Fig. 3), excessive BMP signaling reduces effective mesendoderm C&E movements by reducing medially oriented mesendoderm cell alignment and thus intercalations.

Finally, to test the combined effect of BMP and Nodal signaling on axis elongation in the embryo, we examined whether peak levels of Nodal signaling also provide robustness for embryo body axis extension. In embryos injected with various low amounts of *bmp2b* mRNA (1-5 pg), body axis extension became more variable between embryos, but remained overall largely unchanged compared with controls (Fig. 6F,G). However, when we simultaneously treated BMP overexpressing embryos with 1 µM SB-505124 to reduce peak Nodal signaling levels, we found that axis elongation was consistently reduced (Fig. 6F,G). This points to a conserved function of Nodal signaling in axis extension by effectively repressing BMP function in embryos and explants. To assess further how the concomitant reduction in Nodal signaling and increase in BMP ligand affect axis elongation, we analyzed mesendodermal cell alignment and changes in pSmad5 levels within the dorsal domain of control (DMSO) or 1 µM SB-505124-treated embryos in the presence or absence of ectopic BMP ligand (1 and 5 pg *bmp2b*). This showed that the strongest increase in pSmad5 levels and most disrupted cell alignments were found when BMP ligand was most strongly increased (5 pg *bmp2b*/embryo) and, at the same time, Nodal signaling reduced (1 µM SB-505124; Fig. S10E-G). This is consistent with the notion that the axis elongation defect in these embryos (Fig. 6F,G) is due to defective cell polarization. Strikingly, when correlating changes in pSmad5 levels with cell alignment in individual embryos, we found that also in embryos, which did not show a clearly detectable increase in pSmad5 in the dorsal mesendoderm, cell alignment was affected upon increased BMP ligand and reduced Nodal signaling (Fig. S10H-H″). This suggests that the effect of increased BMP signaling and decreased Nodal signaling on mesendoderm progenitor cell alignment does not strictly require cell-autonomous increases in pSmad5 levels.

## DISCUSSION

Our study describes a thus far understudied role of Nodal signaling in rendering the dorsal mesendoderm less sensitive towards BMP signaling activity during gastrulation movements, thereby providing robustness to axis elongation. Robustness towards extrinsic and intrinsic perturbations is an important feature for development to have a reproducible outcome (Wolpert, 1992; Kitano, 2004; Masel and Siegal, 2009). In the present study, we identify a crucial role for morphogen signaling pathway crosstalk in maintaining the relative spatial organization of distinct signaling activity domains within the gastrula (Fig. 6H), a prerequisite for embryo axis elongation to occur normally even upon variations in morphogen signaling and the overall embryonic context. Cross-regulation between BMP and Nodal signaling displaying opposing morphogenetic activities has also been implicated in zebrafish left-right heart asymmetry (Veerkamp et al., 2013), although how such coordination is realized in different geometric and signaling contexts has not yet been addressed.

Nodal and BMP signaling form activity gradients along the marginal-to-animal and ventral-to-dorsal axes of the zebrafish gastrula, respectively (Schier and Talbot, 2005; Rogers and Müller, 2019; Hill, 2022). Consistent with their partially distinct activity domains, Nodal and BMP signaling can exert opposite functions during patterning by e.g. inducing dorsally derived head structures (Gritsman et al., 2000) and ventral tissues (Kishimoto et al., 1997; Nguyen et al., 1998; Hild et al., 1999; Dick et al., 2000; Schmid et al., 2000), respectively, and during morphogenesis by, for example, differentially regulating cell–cell adhesion (von der Hardt et al., 2007; Barone et al., 2017). Hence, the effective coordination of Nodal and BMP domains is essential to pattern and shape gastrula-stage embryos properly, consistent with our observation that ectopic BMP signaling in dorsal mesendoderm disrupts Nodal-induced cell intercalations and, thus, axis elongation. Strikingly, we found that the BMP signaling function to disrupt effective cell alignment does not strictly require cell-autonomous pSmad5 activation in dorsal tissues, suggesting that BMP signaling activity might affect cell alignment at least in part via long-range effectors and/or through combined effects of Nodal and BMP signaling.

Beyond this necessity to spatially coordinate Nodal and BMP signaling activity for axis elongation, our results show that Nodal signaling actually represses BMP signaling, thereby creating an area of low sensitivity towards BMP effects at the site of mesendoderm induction. Interestingly, although Nodal signaling is required for the early expression of BMP regulators, such as *chrd* or *nog1* together with β-catenin and FGF signaling on the dorsal side of the gastrula (Gritsman et al., 1999; Sirotkin et al., 2000; Maegawa et al., 2006; Varga et al., 2007; Ramel and Hill, 2013) and needs to be activated at pre-gastrula stages to restrict peak pSmad5 levels (Fig. S8G-L), the formation of the pSmad5 gradient appears unaffected by loss of Nodal signaling activity at the onset of gastrulation (Rogers et al., 2020). This lack of Nodal signaling effect on BMP activity might be restricted to pre-gastrula stages given that increased Nodal signaling, as observed in Nodal-type-II-receptor loss of function, is accompanied by a decrease of BMP signaling activity during gastrulation (Preiß et al., 2022). Our data show that, indeed, at late gastrulation stages, peak levels of Nodal signaling in the dorsal mesendoderm render the tissue less sensitive to BMP signaling, a mechanism that might be required to buffer spatial fluctuations in BMP ligand expression within the gastrula. Such a function of Nodal signaling in repressing BMP signaling is supported by our findings in embryos, in which axis elongation defects upon BMP ligand overexpression are more pronounced if peak Nodal signaling levels are reduced, and in explants, in which BMP and Nodal signaling form opposing gradients with Nodal signaling attenuating BMP activity at the site of presumptive explant elongation. Notably, opposing Nodal/BMP activity gradients have recently been shown to arise spontaneously in elongating mouse embryonic stem cell gastruloids (McNamara et al., 2023 preprint), suggesting that their coordinated spatial organization might also be involved in gastruloid morphogenesis.

Why the impact of Nodal signaling becomes more apparent in the explant context than the embryo is still unclear, but it is likely that signaling from the extra-embryonic yolk (Sun et al., 2014) and/or embryo geometry contribute to maintain the pSmad5 gradient within the embryo even when Nodal signaling is absent. Alternatively, or in addition, the increased dependence of the pSmad5 gradient on Nodal signaling in the explants might be due to differences in the spatiotemporal activities of other contributing signaling pathways. In line with this, the expression of several BMP signaling regulators, such as *chrd*, has previously been shown to not only depend on Nodal signaling, but also to require other signaling inputs from FGF and/or β-catenin (Sirotkin et al., 2000; Maegawa et al., 2006; Varga et al., 2007; Ramel and Hill, 2013; Rogers et al., 2020). Moreover, recent studies have shown that reduced Nodal and FGF signaling activity together lead to a strong expansion of the pSmad5 gradient as early as the onset of embryo gastrulation, whereas reducing Nodal signaling alone had only little effect at this stage (Rogers et al., 2020), further supporting the notion of combinatorial signaling being involved in pSmad5 gradient formation.

It has recently been proposed that spatially unconstrained *in vitro* gastrulation models adopt a conserved elongated shape, representing a ‘ground state’ of development (Steventon et al., 2021;  Anlaş and Trivedi, 2021). Consistent with this, zebrafish explants have been shown to elongate upon local Nodal activation (Xu et al., 2014; Fulton et al., 2020; Schauer et al., 2020; Williams and Solnica-Krezel, 2020b; Cheng et al., 2023), a behavior we now show to be associated with medially oriented mesendodermal cell intercalations. As Nodal signaling can, in principle, also trigger ectodermal cell intercalations in zebrafish explants and embryos (Williams and Solnica-Krezel, 2020b), future work will be needed to investigate how the integration of mesendodermal and ectodermal cell dynamics promotes overall axis elongation. In contrast to the prevalence of oriented mesendoderm intercalations during explant elongation, mesendodermal C&E in intact zebrafish embryos requires a combination of collective migration and intercalation movements (Tada and Heisenberg, 2012). Indeed, increasing the population of mesendodermal progenitors associated with migratory behavior by overactivating BMP signaling diminishes body axis elongation in explants and embryos (Myers et al., 2002a; von der Hardt et al., 2007). Considering previous work showing that mediolateral cell intercalations drive *in vitro* axis elongation in *Xenopus* tissue explants (Huebner and Wallingford, 2018; Keller and Sutherland, 2020), our results suggest that cell intercalations constitute a conserved mechanism for elongating mesenchymal embryonic tissues independently of the specific embryonic context. In this process, BMP signaling needs to be tightly spatially controlled, likely for segregating the domain of cell migration, accumulating cells dorsally, versus dorsal cell intercalation, elongating the body axis. As ectopic BMP signaling also interferes with effective elongation of *Xenopus* explants and embryos and mouse embryonic stem cell gastruloids (Graff et al., 1994; De Robertis et al., 2017; Turner et al., 2017; Yoon et al., 2021), such potential balancing function of migration and intercalation could be conserved. Further identification of molecular and cellular effectors by which BMP signaling changes morphogenetic capacities in different organisms will be key to generating insights into the conserved and species-specific BMP functions that shape gastrula-stage embryos.

Properly shaping embryonic tissues requires a large and versatile morphogenetic toolkit, as embryonic morphologies vary widely. For instance, in zebrafish embryos, which spread over a large extra-embryonic yolk cell over the course of gastrulation, mesendoderm is induced in a narrow band of cells all around the germ ring margin, whereas in zebrafish explants, lacking a yolk cell, mesendoderm arises from a local, compact domain (Schier and Talbot, 2005; Rogers and Müller, 2019; Hill, 2022; Schauer et al., 2020; Fulton et al., 2020). This geometrical constraint potentially affects BMP signaling function, capable of forming a long-range activity gradient, in balancing cell migration versus intercalation. It is conceivable that the BMP function of suppressing premature cell intercalations becomes particularly important in embryos, in which mesendoderm specification is spread over long distances and cells need to undergo a long-range convergence movement before arriving at the dorsal side where cell intercalations elongate the body axis. Future work will be needed to address how the activity of BMP signaling during gastrulation has been adapted to the specific embryo geometry in different species to ensure a proper balance between convergent cell migration and dorsal cell intercalation.

## MATERIALS AND METHODS

### Fish lines and husbandry

Maintenance and handling of zebrafish (*Danio rerio*) was performed as previously described (Westerfield, 2000). The following zebrafish strains were used in this study: wild-type AB or a cross of wild-type ABxTL, Tg(*sebox*::*EGFP*) (Ruprecht et al., 2015), MZ*oep* (Gritsman et al., 1999), MZ*lefty1,2* (Rogers et al., 2017), MZ*oep*;Tg(*gsc::EGFP-CAAX*), MZ*lefty1,2*;Tg(*sebox*::*EGFP*), *chordin*^tt250^ (Hammerschmidt et al., 1996), *chordin*^tt250^;Tg(*sebox*::*EGFP*), MZ*lefty1,2*;*chordin*^tt250^;Tg(*sebox*::*EGFP*) and MZ*lefty1,2*;*chordin*^tt250^. Mutant transgenic lines were generated by crosses of the Tg(*sebox*::*EGFP*) and Tg(*gsc::EGFP-CAAX*) lines with the respective mutants. Raising and genotyping of MZ*oep*, MZ*lefty1,2* and *chordin*^tt250^ was performed as described (Gritsman et al., 1999; Rogers et al., 2017; Pomreinke et al., 2017). Embryos were raised in E3 medium or Danieau's solution [58 mM NaCl, 0.7 mM KCl, 0.4 mM MgSO_4_, 0.6 mM Ca(NO_3_)_2_, 5 mM HEPES, pH 7.6] at 25-31°C and staged according to Kimmel et al. (1995). All animal experiments were performed in accordance with the guidelines of the national Animal Experimentation Commission at the Federal Ministry (ETK) of Austria in line with EU and national legislation.

### Blastoderm explant preparation

Blastoderm explants were prepared as previously described (Schauer et al., 2020). In short, the entire blastoderm was removed from the yolk cell at the 256-cell stage using forceps and cultured at 25-31°C in Danieau's solution. Staging of the explants was performed based on sibling embryos from the same egg lay. After approximately 1 h, explants that did not close up properly or showed delayed cleavages were removed. A stereo-microscope (Olympus SZX 12) with a QImaging Micropublisher 5.0 camera was used to take brightfield images of explants for analyzing explant morphology.

### Embryo microinjections

To synthesize mRNAs, the mMessage mMachine Kit (Ambion) was used. One-cell stage and 32- to 128-cell stage injections were performed as described (Westerfield, 2000). The following mRNAs were used: 30-50 pg *H2A-mChFP* (Arboleda-Estudillo et al., 2010), 50-100 pg *membrane-RFP* (Iioka et al., 2004), 50-100 pg *membrane-GFP* (Kimmel and Meyer, 2010), 3-30 pg *constitutively active Alk8* (*caAlk8*) (Bauer et al., 2001; Payne et al., 2001), 1-5 pg *bmp2b* (Kishimoto et al., 1997), 37 pg *chrd-GFP* (Pomreinke et al., 2017) and 10 pg *cyclops* (Rebagliati et al., 1998). For clonally labeling nuclei and/or F-actin, 5 pg *H2A-mChFP* and/or 10 pg *lifeact-RFP* (Behrndt et al., 2012) were injected into a single blastomere at the 32- to 128-cell stage. For overexpressing *caAlk8* in one or two blastomeres, 6 pg *caAlk8* were injected at the 8- to 16-cell stage together with 5 pg *H2A-mChFP*. The following morpholinos were used in this study: 1.5 nl 0.25 mM *has2*MO (5′-AGCAGCTCTTTGGAGATGTCCCGTT-3′) (Bakkers et al., 2004; von der Hardt et al., 2007), 1.5 nl 0.5 mM *smad5*MO (5′-AACAGACTAGACATGGAGGTCATAG-3′) (Lele et al., 2001a; von der Hardt et al., 2007), 1.5 ng *bmp2b*MO (5′-CGCGGACCACGGCGACCATGATC-3′) (Lele et al., 2001a), 4.5 ng *chordin*MO (5′-ATCCACAGCAGCCCCTCCATCATCC-3′) (Nasevicius and Ekker, 2000), 3 ng *noggin1*-MO1 (5′-GCGGGAAATCCATCCTTTTGAAATC-3′) (Dal-Pra et al., 2006), 4 ng *noggin1*-MO2 (5′-GAGATTAAACGCGGGATTTATCCGT-3′) (Dal-Pra et al., 2006) and 4-5 ng control MO (human β-globin MO 5′-CCTCTTACCTCAGTTACAATTTATA-3′). All morpholinos were obtained from GeneTools.

### Live imaging of blastoderm explants and embryos

Sample preparation for live-imaging experiments of explants on upright confocal microscopes was performed as described previously (Schauer et al., 2020) by mounting explants in 800×800 µm agarose molds (Microtissues) in Danieau's solution. Unless otherwise indicated, live imaging on upright microscopes was started when control embryos had reached 50% epiboly stage and explants were oriented in side-view. Imaging was performed using a Zeiss LSM 900 upright microscope equipped with a Zeiss Plan-Apochromat 20×/1.0 water-immersion objective. For high-magnification analysis of cell alignment in blastoderm explants at inverted confocal microscopes, small pieces of 800×800 µm agarose molds were cut and immobilized on glass-bottom dishes (35 mm, MatTek Corporation, P35G-1.5-14-C) with agarose. For analysis of cell alignment in embryos, the embryos were mounted in 0.7% low-melting-point agarose in Danieau's solution on the same type of glass-bottom dishes with the dorsal side, as identified by *sebox:EGFP* signal, facing the objective. Imaging was started at 90% epiboly, unless otherwise indicated, and performed on a Zeiss LSM880 inverted microscope with a Zeiss Plan-Apochromat 40×/1.2 water-immersion objective. In all cases, the temperature during image acquisition was set to 28.5°C.

### Whole-mount *in situ* hybridization (WMISH)

Explants for WMISHs were fixed overnight at 4°C in 4% paraformaldehyde (PFA), washed five times in PBS, transferred to 100% methanol and stored at −20°C until further processing. WMISHs with digoxigenin (DIG)-labeled antisense RNA probes were then performed as described previously (Thisse and Thisse, 2008). Antisense RNA probes were generated using Roche DIG-modified nucleotides with SP6, T7 or T3 RNA polymerase from mMessage mMachine kits (Thermo Fisher Scientific, AM1344). The following RNA probes were used: *chrd* (Schulte-Merker et al., 1997), *hgg* (Thisse et al., 1994), *flh* (Talbot et al., 1995), *papc* (Yamamoto et al., 1998), *tbx16* (Griffin et al., 1998) and *draculin* (Herbomel et al., 1999). Imaging of WMISHs was performed with a QImaging Micropublisher 5.0 camera on an Olympus SZX 12 stereomicroscope.

### Assessment of mesendoderm domain morphology in conjunction with explant morphology

To analyze mesendoderm induction and explant elongation upon reduction of Nodal signaling, explants were treated with either 0.1% DMSO (controls) or 0.5 µM or 1 µM SB-505124 (Byfield et al., 2004) from the 256-cell stage until fixation at bud stage. To analyze BMP-dependent changes in mesendoderm morphology, BMP signaling was overactivated by injection of *caAlk8*, *bmp2b* mRNA or *chrd* MO together with *membraneRFP* or *H2A-mChFP* mRNA (as described above in the ‘Embryo microinjections’ section) in a *sebox*::*EGFP* transgenic background. Amounts of mRNA and morpholino injected for specific experiments are indicated in the respective figure legends. Explants were raised until bud stage, then fixed in 4% PFA overnight at 4°C. To analyze explant elongation and clone localization upon local injection of 6 pg *caAlk8* and/or 5 pg *H2A-mChFP* mRNA, embryos were injected (as described above in the ‘Embryo microinjections’ section) in one or two blastomeres at the 8- to 16-cell stage to create large clones of different localization and fixed at bud stage. The explants were then washed five times in PBS+0.1% Tween 20 and mounted in 0.7% low melting point agarose in 2% agarose molds in side-view for imaging on a Zeiss LSM 800 upright microscope equipped with a Zeiss Plan-Apochromat 20×/1.0 water-immersion objective.

### Whole-mount immunofluorescence

Immunostainings were performed similarly for blastoderm explants and intact embryos. Anti-pSmad5 (1:100; 9516S, Cell Signaling Technology) whole-mount immunofluorescence was performed as described (Pomreinke et al., 2017; Schauer et al., 2020). As secondary antibody, goat anti-rabbit-Alexa Fluor 546 was used (1:500; A11010, Thermo Fisher Scientific). For labeling nuclear pSmad5 together with cell outlines, anti-β-Catenin (1:500; C7207, clone 15B8, Sigma-Aldrich) and chicken anti-GFP (Soh et al., 2020) (1:200; GFP-1020, Aves Labs) antibodies were used for better visualizing the *sebox::EGFP* domain. As secondary antibody, goat anti-chicken-Alexa Fluor 488 (1:500; A11039, Invitrogen) and goat anti-mouse-Alexa Fluor 647 (1:500; A21235, Invitrogen) were additionally added. Anti-pSmad5 and anti-pSmad2 (1:1000; 8828, Cell Signaling Technology) double whole-mount immunofluorescence was performed as described by Soh et al. (2020) with goat anti-rabbit-horseradish peroxidase (1:500; AB_2307391, Jackson ImmunoResearch) and TSA fluorescein detection of pSmad2 activity (Soh et al., 2020). The immunostained blastoderm explants and embryos for gradient analysis were mounted in 2% agarose molds at a Zeiss LSM 880 upright microscope equipped with a Zeiss Plan-Apochromat 20×/1.0 water-immersion objective. Explants were oriented in side-view using either the *sebox::EGFP* (for wild type and MZ*lefty1,2*) or *gsc::EGFP-CAAX* (for MZ*oep*) signal as a marker. Embryos were oriented to be viewed from the animal pole. Imaging conditions between different samples and replicates were kept similar. Explants and embryos co-stained with anti-pSmad5, anti-β-Catenin and anti-GFP for analysis of pSmad5 intensity and cell alignment were mounted on glass-bottom dishes (35 mm, MatTek Corporation, P35G-1.5-14-C) in 0.7% low-melting point agarose in side-view (explants) and dorsal view (embryos), respectively, and imaged on a Zeiss LSM880 inverted microscope with a Zeiss Plan-Apochromat 40×/1.2 water-immersion objective

For staining of pSmad5 in the absence of *chrd*, explants were prepared from either MZ*lefty1,2*;*chrd^tt250^* females crossed with MZ*lefty1,2*;*chrd^tt250^;*Tg(*sebox::EGFP*) mutant males or from crosses of *chrd*^tt250^ females and *chrd^tt250^*;Tg(*sebox::EGFP*) males treated with DMSO (control) or 1 µM SB-505124 and fixed at 75% epiboly. For analyzing the pSmad5 domain in absence of *chrd* and *nog1* activity, explants were prepared from MZ*lefty1,2*; *chrd^tt250^* females crossed with MZ*lefty1,2*;*chrd^tt250^*;Tg(*sebox::EGFP*) mutant males or from crosses of *chrd*^tt250^ females and *chrd^tt250^*;Tg(*sebox::EGFP*) males injected with 4 ng control morpholino or 3 ng *nog1*MO1 and fixed at 75% epiboly. Immunostaining was performed as described above, also adding chicken anti-GFP (Soh et al., 2020) antibody (1:200; GFP-1020, Aves Labs) and as secondary antibody goat anti-chicken-Alexa Fluor 488 (1:500; A11039, Invitrogen) to better visualize the *sebox::EGFP* domain. After imaging, the explants were genotyped as described (Pomreinke et al., 2017) to identify homozygous mutants.

For immunostaining of pSmad5 upon simultaneous overactivation of BMP signaling and reduction in Nodal signaling activity, wild-type embryos were injected with 1 pg *bmp2b* and 80 pg *membrane-GFP* mRNA at the one-cell stage and treated with 0.1% DMSO and 1 µM or 10 µM of the Nodal inhibitor SB-505124 (Byfield et al., 2004; Rogers et al., 2017), respectively, from the 4- to 16-cell stage until 75% epiboly. Fixation and further processing for immunostaining was performed as described above.

For analyzing the time window during which Nodal signaling was required to restrict BMP signaling activity, control explants were treated from the 256-cell stage until 75% epiboly with 0.1% DMSO; explant treatments with 40 µM of the Nodal inhibitor SB-505124 (Byfield et al., 2004; Rogers et al., 2017) were performed from the 256-cell stage until 75% epiboly, from the 256-cell stage until shield stage, and from shield stage until 75% epiboly. For embryos, control embryos were treated with 0.1% DMSO from the 4- to 16-cell stage until 75% epiboly; embryo treatments with 40 µM of the Nodal inhibitor SB-505124 (Byfield et al., 2004; Rogers et al., 2017) were performed from the 4- to 16-cell stage until 75% epiboly, from the 4- to 16-cell stage until shield stage, and from shield stage until 75% epiboly. Embryos and explants were fixed at 75% epiboly stage and immunostainings for pSmad5 were performed as described above.

### Embryo axis length measurements

To analyze embryo axis length upon BMP overactivation, Tg(*sebox::EGFP*) embryos were injected with 80 pg *membraneRFP* mRNA (for controls) or 30 pg *caAlk8* with 80 pg *membraneRFP* mRNA and fixed at bud stage in 4% PFA at 4°C overnight. To analyze embryo axis length upon BMP overactivation and simultaneous lowering of Nodal signaling levels, Tg(*sebox::EGFP*) embryos were injected with *membrane-RFP* or *H2A-mChFP* (for controls) or 1-5 pg *bmp2b* with *membrane-RFP* or *H2A-mChFP* mRNA and treated with 0.1% DMSO (control) or 1 µM SB-505124 (Byfield et al., 2004; Rogers et al., 2017) from the 4- to 16 cell stage until bud stage and fixed at bud stage in 4% PFA at 4°C overnight. The embryos were washed in PBS+0.1% Tween 20 and mounted in 0.7% low-melting-point agarose in lateral view on glass-bottom dishes (µ-Slide 4 Well, Ibidi) for imaging on a Zeiss LSM880 inverted microscope with a Zeiss Plan-Apochromat 10× objective.

### Quantitative real-time PCR (qRT-PCR)

Total RNA extraction of ten embryos or explants from wild-type, MZ*oep* or MZ*lefty1,2* backgrounds at 75% epiboly was performed using 0.5 ml TRIzol (Invitrogen) according to the manufacturer's protocol. Genomic DNA removal was performed using the DNA-free DNA Removal Kit (Thermo Fisher Scientific) according to the manufacturer's protocol. cDNA and negative control NO-RT cDNA reactions were performed using the iScript™ Reverse Transcription Supermix for qRT-PCR according to the manufacturer's protocol with 500 ng total RNA as starting material. Primers for qRT-PCR were checked for linear amplification by a concentration series of cDNAs. For the experiment, a 1:10 cDNA dilution was used. As a housekeeping gene for normalization, we used *elongation factor 1 α* (*EF1α*) (Miesfeld et al., 2015). Primers for *chrd* (5′-CGACTCTTCCACCAATCACA-3′, 5'-CAGATACGCCGTACCTTCAT-3′) and *nog1* (5′-TGACACTTTACCCCTGCTGG-3′, 5′-GAAAGCGGCTGTCAAAGTCC-3′) were designed using Primer3. The qPCR SYBR Green Mix (Thermo Fisher Scientific) was used for all qRT-PCRs, which were run on a Bio-Rad C1000 Thermal Cycler. Reactions were performed in triplicate.

### Image analysis

Image analysis was performed using Bitplane Imaris or Fiji (Schindelin et al., 2012).

### Analysis of explant morphology

For quantifying explant and mesendoderm morphogenesis in high-resolution, live-imaging time-lapse movies, the onset of extension was defined as the time point after which a clear elongation of the explant could be seen in more than three subsequent frames on a 3D reconstruction using Bitplane Imaris. To quantify the shape of the explants, images were exported to Fiji and the explant circumference was outlined based on brightfield images to generate a binarized image of the explant and the background by thresholding. A reference axis was defined by drawing a 40-pixel-wide box in the center of the explant. The morphoLibJ geodisc distance map function was used in Fiji to calculate intensity values based on the distance of each pixel in the explant from the reference axis. These intensities were then used to reconstruct the explant shape by measuring the intensity values of the furthest points from the axis on one side of the explant.

For length measurements of explants at specific developmental stages, the distance from the tip of the explant to the back was measured in Fiji using the segmented line tool on brightfield side-view images. Quantification of blastoderm explant morphology on brightfield side-view images was performed as described previously (Schauer et al., 2020). In short, explants were considered extended if a clear indentation could be seen between the round and extended part of the explant. Explants were manually outlined to quantify explant circularity using the Circularity plugin in Fiji. Extended and not extended explants were pooled in this analysis. The length of the explant extension was quantified by measuring the distance from the tip of the explant to the indentation point with the ‘segmented line’ tool from Fiji and normalized to the full explant length. Only explants for which the extension was clearly visible were included in this analysis.

For measuring explant length upon local *caAlk8* overexpression, clones were categorized based on clone localization at bud stage into ‘mostly mesendodermal’ (if <25% of the clone was outside of the *sebox*::*EGFP*-positive domain), ‘mostly ectodermal’ (if <25% of the clone was inside of the *sebox*::*EGFP*-positive domain) or ‘ectodermal plus mesendodermal’ clones (if at least 25% of the clone could be found in both domains). The segmented line tool was then used in Fiji to measure the length from the tip of the extension to the base of the extension and the length of the whole explant on maximum intensity projections based on brightfield images in side-view.

### Analysis of *sebox*::*EGFP* domain morphology

To approximate the proportion of mesendoderm within the extension of explants, the onset of explant extension was identified as described above. In Fiji, a ‘SUM’ projection was made at the indicated time points after the onset of extension. The image was then binarized by thresholding and a median filter of 2-pixel width was applied to reduce noise. The circumference of the extension and base of the extension were outlined on the brightfield images using the freehand selection tool in Fiji. This mask was then applied to quantify the area fraction on the binarized *sebox::EGFP* image to measure the proportion of mesendodermal tissues in the extension. Note that for this quantification, explants where the ectoderm overlaid mesendoderm in the plane of the projection had to be excluded. To analyze the localization of the *sebox::EGFP* domain along the extension, images were binarized as described. A 50-pixel-wide line was drawn in the middle of the explant from the tip of the extension towards the end of the explant, and the plot profile function was used to obtain intensity values along this axis.

To quantify the length and width of the *sebox::EGFP* domain over time, the segmented line tool was used in Fiji to measure the extent of the EGFP signal in the direction of the extension (length) and the extent of the EGFP signal in the middle of the domain perpendicular to the extension (width). To quantify the length/width ratio of the *sebox::EGFP* domain upon BMP overactivation, length and width were similarly determined. The area of the *sebox::EGFP* domain was measured by manually outlining the GFP-positive domain using the freehand selection tool in Fiji on maximum intensity projections. The boundaries of the domain were confirmed by looking through individual sections to reduce information loss due to the projection.

### Analysis of clone dispersal

To automatically track clone cells in blastoderm explants in 3D based on their nuclear signal at a time resolution of less than 3 min, the ‘spot detection’ plugin from Imaris was employed. Tracking parameters were set using autoregressive motion and allowing a gap of one frame in the tracks and a maximum distance of 10 µm between subsequent time points. All tracks were manually verified, and incomplete tracks were either removed or manually completed to encompass the whole analysis time frame. The tracks were divided into mesendodermal and ectodermal cells based on their location within the *sebox::EGFP*-positive or -negative domain, respectively, and their 3D coordinates were extracted for further analysis. We used a custom MATLAB script (doi:10.15479/AT:ISTA:14926) to calculate the mean distance of each cell to all clone cells in the direction of the extension and perpendicular to it in a 2D coordinate system. As explants were not stably positioned during imaging, the main explant axis was defined manually by placing a spot at the bottom of the analysis volume at the extension tip every ten time frames and a stable spot at the round part of the explant opposite the extension. All spots were projected along their *z*-axis, and the mean distance of each cell to other cells in the clone was measured either in the direction of the extension or perpendicular to it, relative to the initial time point. Notably, tracks that began at later time points were excluded from the analysis.

### Analysis of cell alignment

Cell alignment was measured as described previously (Williams and Solnica-Krezel, 2020b). In short, explants and embryos were oriented with the direction of explant elongation and anterior pole of the embryo pointing up in Fiji, as identified based on short time-lapse movies. Rotation of embryos was performed in 3D in Imaris in cases for which the dorsal side of the embryo was not parallel to the imaging plane. The outlines of the cell body were drawn based on membrane or Lifeact (F-actin) signal on individual planes for live samples using the freehand selection tool in Fiji, and the angle of the longest axis of the best fit ellipse to the main explant or mediolateral embryonic axis was measured.

To analyze cell alignment on fixed samples in conjunction with pSmad5 intensities, explants and embryos were oriented as here described for live samples, and the outlines of the cell body drawn based on the β-Catenin signal on individual planes using the freehand selection tool in Fiji. DAPI and pSmad5 intensity were measured in a 6-pixel-wide circle in the nucleus, as determined based on the DAPI signal. For background subtraction, pSmad5 intensity was measured in a 4-pixel-wide circle in five random positions within the cytoplasm in the center of the analyzed stack. Cell alignment and pSmad5 intensities were measured 45-60 µm from the top of the explants and within 37.5 µm of the top of the mesendoderm in embryos to reduce depth-related effects for intensity measurements. For embryos, 20-30 cells were analyzed per sample. For explants, 9-27 cells were analyzed per sample.

### Analysis of gene expression domains

The area of gene expression domains was determined by outlining the signal based on *in situ* hybridizations in Fiji using the freehand selection tool and normalized to the area of the whole explant. The length of the explants was measured using the segmented line tool in Fiji. To exclude the possibility that explants were lacking Nodal signaling altogether, only explants forming an elongation were considered for this analysis.

### Analysis of pSmad5 and pSmad2 nuclear localization

Signaling pathway activity profiles were determined as described previously using a custom MATLAB script (Schauer et al., 2020). In short, the spot detection function in Imaris was used to automatically determine nuclear coordinates based on DAPI signal. Nuclei were analyzed to a depth of 180 µm from the top for explants in side-view and at 60-120 µm from the animal pole for embryos. Enveloping layer nuclei, yolk syncytial layer nuclei and nuclei with inhomogeneous DAPI signal as well as clearly dividing cells were manually excluded before further analysis steps as described previously (Schauer et al., 2020). Reference points to calculate signaling activity profiles were determined by manually drawing a line of nuclei using the same spot detection tool on the side of highest pSmad5 intensity in explants and embryos. 3D coordinates for the reference points and all nuclei as well as mean fluorescence intensities for the nuclei were then extracted, and a previously published custom MATLAB script (Schauer et al., 2020) was used to calculate the geometric distance of all nuclei in the explant or embryo from the reference points upon projection along their *z*-axis.

Background subtraction was performed by manually placing 12 spots in the cytoplasm of cells located on the low pSmad5 side at the bottom of the analysis volume. For double stainings of pSmad5 and pSmad2, 12 spots were placed in the cytoplasm of cells located on either side at the bottom of the analysis volume. pSmad5 and pSmad2 intensities were normalized to the DAPI signal to correct for artifacts due to imaging depth and high-intensity nuclei were manually re-checked to exclude nuclei with potential artificially high signal due to very weak or inhomogeneous DAPI staining or dividing nuclei following the criteria for the initially described correction. To account for differences in explant and embryo size, the calculated distances were normalized to the maximum distance in each explant/embryo. To plot the normalized pSmad5 and pSmad2 profiles from the low pSmad5 signaling side, the data were binned into steps of 0.04 for explants and 0.02 for embryos, as this bin size corresponds to approximately two cell diameters, respectively, and averaged across the experimental replicates. The first and last 0.04 bin of explants and embryos were excluded because they contained only few nuclei owing to the sample curvature. Note that for intensity measurements the same number of control and treated embryos or blastoderm explants were analyzed per replicate in each graph to reduce the inherent variability due to processing and imaging on different experimental days between replicates. The intensities are shown relative to the average intensity in the first bin closest to the high pSmad5 and the high pSmad2 side of control samples.

To quantify the extent of the pSmad5- and *sebox::EGFP*-positive domains along the explant axis, we generated maximum intensity projections from the middle plane of the explant to the top of the explant in Fiji. We then measured the distance from the back of the explant to the end of the pSmad5-positive domain, as assessed by the presence of clearly visible nuclear pSmad5 staining, in the center of the explant using the ‘line’ tool. Similarly, we determined the distance from the back of the explant to the start of the mesendoderm as assessed by the presence of *sebox::EGFP* signal and normalized to the overall length of the explant.

Categorizing pSmad5 profiles as graded or radial in control conditions or upon overexpression of 1 pg *bmp2b* plus treatment with 1 µM or 10 µM SB-505124 was achieved by defining a radial distribution when nuclear pSmad5 levels appeared uniformly elevated, whereas in graded conditions, a clear difference in pSmad5 levels along the prospective DV axis could be found.

Comparison of the changes in the distribution of high pSmad5 signaling nuclei upon perturbation of Nodal signaling between explants and embryos was performed by determining the mean position, i.e. the distance of the nuclei from the reference point normalized to the sample length, of the top 10% highest intensity pSmad5 nuclei in wild-type/DMSO-treated explants and embryos. The distance of the top 10% high-intensity pSmad5-positive nuclei in MZ*oep* and 1 µM SB-505124-treated explants and embryos relative to the average distance of the corresponding control nuclei was then determined.

### Analysis of embryonic axis length

The length of the mesendoderm along the AP axis, referred to as axis length, was determined by measuring the distance from the front of the *sebox::EGFP* domain to the tailbud using the segmented line tool along the curvature of the embryo in Fiji on maximum intensity projections. To normalize for differences in embryo size, the overall size of the embryo, referred to as embryo height, was determined by measuring the distance from the head domain of the embryo to the tailbud.

### Statistics

GraphPad Prism was used to perform statistical analysis and generate plots. The number of individual analyzed blastoderm explants or embryos (*n*) and independent biological replicates (*N*) are stated in the figure legends. No inclusion or exclusion criteria were used for analyzed samples. No statistical tests were performed for assessing the sample sizes. Error bars in box plots correspond to the minimum and maximum in the dataset with the box indicating the upper and lower quartiles, and the horizontal line indicating the median of the datasets. Error bars in graphs correspond to either ±s.d. or ±s.e.m., as indicated on the respective axis labels. All data were tested for normality using the D'Agostino–Pearson normality test before choosing the appropriate statistical test to assess significance. In Fig. S3I, the sample size was insufficient for the D'Agostino–Pearson normality test in one category and we thus used the Shapiro–Wilk test to assess normality. For comparison of two groups, we used a two-sided Student's *t*-test or the Mann–Whitney test, depending on whether the datasets showed a normal distribution or not. For comparison of more than two sample groups, we used a one-way ANOVA or the Kruskal–Wallis test, depending on whether the datasets showed a normal distribution or not. A correction for multiple comparisons was performed in such cases. The specific statistical tests as well as exact *P*-values are stated in the figure legends.

## Supplementary Material



10.1242/develop.202316_sup1 Supplementary information
