# Supplementary material for: Robust axis elongation by Nodal-dependent restriction of BMP signaling
Source: Development. 2024 Feb 19;151(4):dev202316. doi: 10.1242/dev.202316 (PMC10911127; doi:10.1242/dev.202316)
Supplement: Supplementary information [file develop-151-202316-s1.pdf]

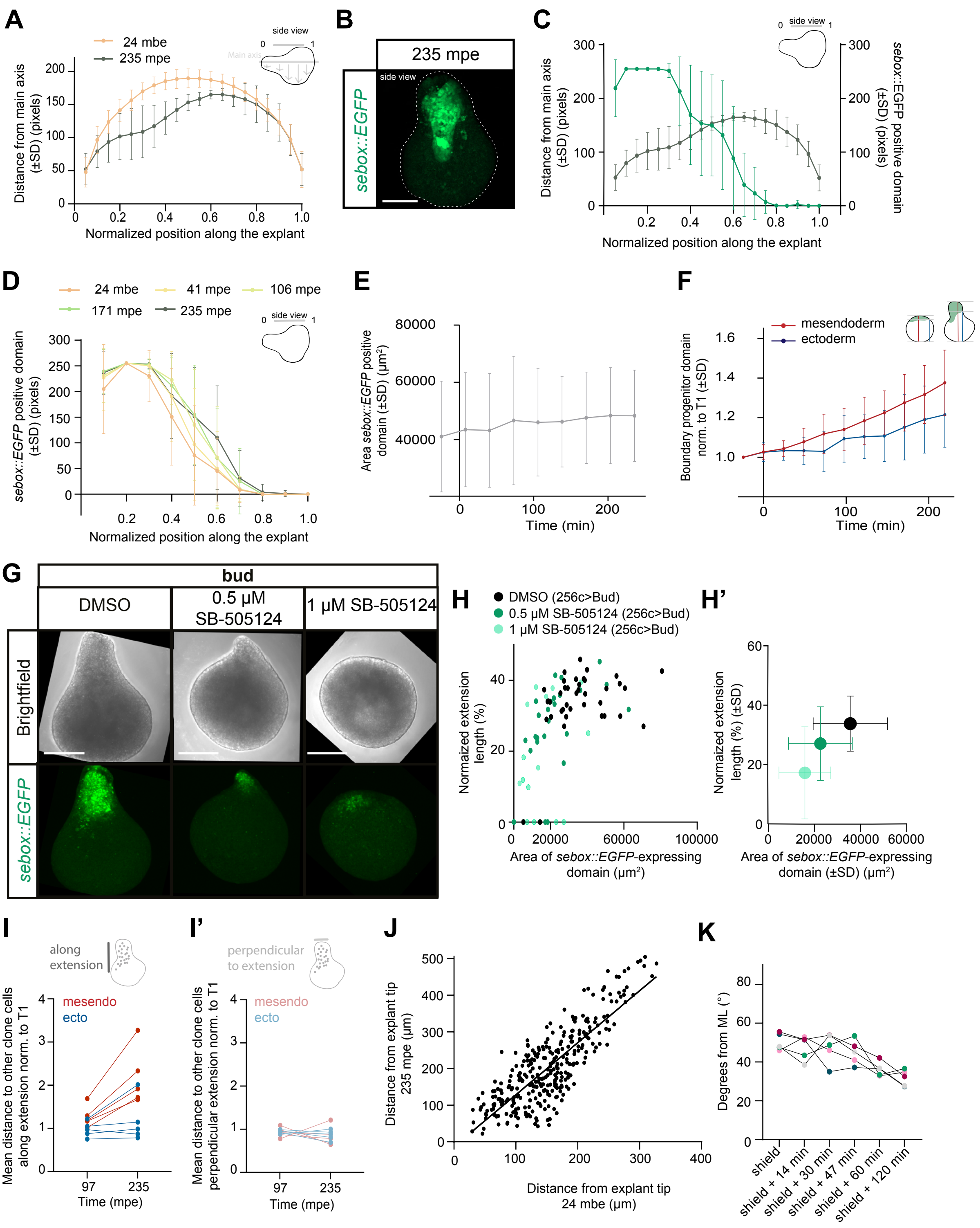

Development • Supplementary information

Fig. S1. Characterization of blastoderm explant elongation. (**A**) Blastoderm explant shape assessed by the distance from the rim to the middle axis of the explant 24 min before the onset of explant elongation (mbe) and 235 minutes post explant elongation onset (mpe) (n=6, N=6). (**B**) Maximum intensity projection of fluorescence images (side views) of blastoderm explants obtained from Tg(*sebox::EGFP*) embryos expressing EGFP (green) in mesendoderm progenitors at 235 mpe. (**C**) Blastoderm explant shape assessed by the distance from the rim to the middle axis of the explant at 235 mpe and frequency of EGFP expression in blastoderm explants obtained from Tg(*sebox::EGFP*) embryos illustrating the position of mesendodermal tissues along the blastoderm explant axis (n=6, N=6). (**D**) Likelihood of EGFP expression as a function of the normalized position along the explant main axis in blastoderm explants obtained from Tg(*sebox::EGFP*) embryos, illustrating the position of mesendodermal tissues along the blastoderm explant axis at different timepoints of explant elongation (n=6, N=6). (**E**) Area of the EGFP expression domain in blastoderm explants obtained from Tg(*sebox::EGFP*) embryos at different timepoints (n=6, N=6). (**F**) Domain boundary of the EGFP positive and EGFP negative domain in blastoderm explants obtained from Tg(*sebox::EGFP*) embryos at different timepoints normalized to timepoint 1 at 24 minutes before the explant elongation (mbe; n=5, N=5). (**G**) Maximum intensity projection of fluorescence images (side views) of blastoderm explants obtained from Tg(*sebox::EGFP*) embryos expressing EGFP (green) in mesendoderm progenitors at bud stage treated with DMSO (treated from 256c to bud: n=39, N=4) or Nodal inhibitor (SB-505124; treated from 256c to bud with 0.5 μM SB-505124: n=25, N=4; or 1 μM SB-505124: n=16, N=4). (**H,H'**) Area of the EGFP expression domain in blastoderm explants versus the normalized extension length in explants obtained from Tg(*sebox::EGFP*) embryos treated with DMSO (treated from 256c to bud: n=39, N=4) or Nodal inhibitor (SB-505124; treated from 256c to bud with 0.5 μM SB-505124: n=33, N=4; or 1 μM SB-505124: n=30, N=4) at bud stage. (H). (**H'**) Area of the EGFP expression domain in blastoderm explants obtained from Tg(*sebox::EGFP*) embryos versus the normalized extension length, shown as mean +/- SD, treated with DMSO (treated from 256c to bud: n=39, N=4) or Nodal inhibitor (SB-505124; treated from 256c to bud with 0.5 μM SB-505124: n=25, N=4; or 1 μM SB-505124: n=16, N=4) at bud stage. Notably, only explants which had formed mesendoderm, as assessed by Tg(*sebox::EGFP*) expression, were considered. (**I,I'**)

Development • Supplementary information

Clone dispersal parallel (**I**) and perpendicular (**I'**) to the axis of explant elongation assessed by the mean distance of each cell in the clone to other clone cells at two timepoints during explant elongation (97 mpe and 235 mpe) for each individual explant corresponding to Fig. 1H,I (n=5, N=5). (**J**) Correlation between the position of clonally labeled cell nuclei in the mesendoderm before the onset of explant elongation (24 mbe) and during explant elongation (235 mpe) relative to the tip of the extension in wildtype explants (R^2=0.6748, N=5). (**K**) Mean cell alignment assessed by the deviation (degrees) of the main cell extension axis from the main mediolateral explant axis at the onset of explant elongation (shield) and during explant elongation (shield + 14 min, shield + 30 min, shield + 47 min, shield + 60 min, shield + 120 min) for each individual explant corresponding to Fig. 1K (n=5, N=5). Each color indicates a different explant. Scale bars: 200 µm (**B,G**).

Development • Supplementary information

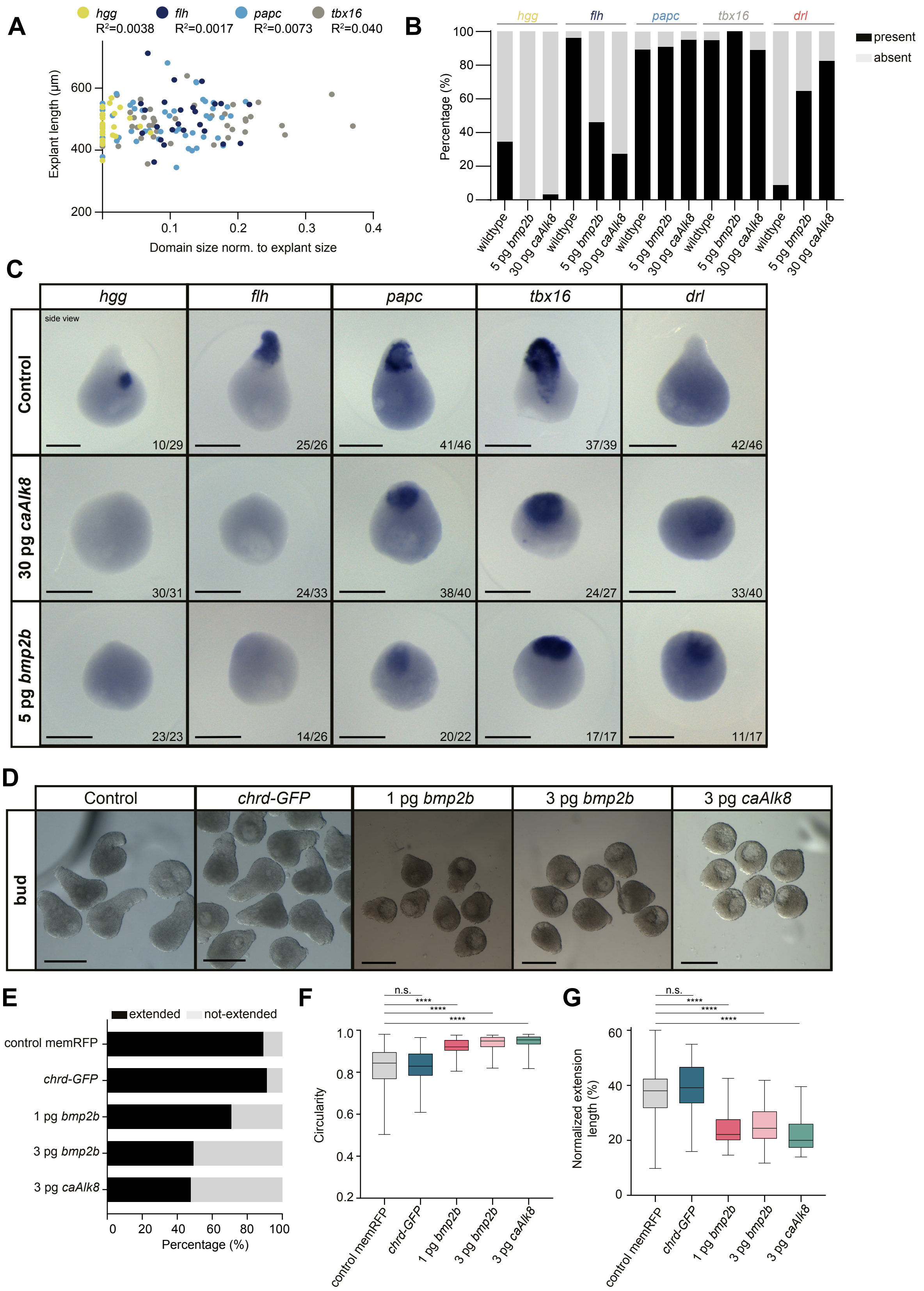

Development • Supplementary information

Fig. S2. Changes in mesendoderm marker expression and blastoderm explant morphogenesis upon BMP signaling alterations. (**A**) Relationship between the length of blastoderm explants and the size of mesendodermal marker expression as assessed by *in situ* hybridization normalized to explant size at bud stage (*hgg*: n=29, N=3; *flh*: n=24, N=3; *papc*: n=47, N=5; *tbx16*: n=39, N=4). (**B**) Percentage of blastoderm explants from wildtype embryos, and embryos injected with 5 pg *bmp2b* or 30 pg *caAlk8* mRNA at bud stage expressing/not expressing prechordal plate (*hgg*: wildtype: n=29, N=3; 5pg *bmp2b*: n=23, N=2; 30pg *caAlk8*: n=31, N=3), notochord (*flh*: wildtype: n=26, N=3; 5pg *bmp2b*: n=26, N=2; 30pg *caAlk8*: n=33, N=3), paraxial mesoderm (*papc*: wildtype: n=46, N=5; 5 pg *bmp2b*: n=22, N=2; 30 pg *caAlk8*: n=40, N=4), ventrolateral mesoderm (*tbx16*: wildtype: n=39, N=4; 5 pg *bmp2b*: n=17, N=2; 30 pg *caAlk8*: n=27, N=4) or ventral mesoderm (*drl*: wildtype: n=46, N=3; 5 pg *bmp2b*: n=17, N=2; 30 pg *caAlk8*: n=40, N=3) markers assessed by *in situ* hybridization. All embryos were co-injected with 50-100 pg *memRFP* as injection control. (**C**) Expression of prechordal plate (*hgg*), notochord (*flh*), paraxial mesoderm (*papc*), ventrolateral mesoderm (*tbx16*) and ventral mesoderm (*drl*) markers assessed by *in situ* hybridization in blastoderm explants from wildtype embryos, and embryos injected with 5 pg *bmp2b* or 30 pg *caAlk8* mRNA (side views) at bud stage. Images correspond to quantifications in (B). The proportion of explants with a similar expression pattern to the shown image is indicated in the lower right corner. (**D**) Single-plane bright-field images of blastoderm explants from wildtype embryos (control: n=236, N=13), and embryos injected with 37 pg *chrd-GFP* (n=45, N=4), 1 pg *bmp2b* (n=41, N=5), 3 pg *bmp2b* (n=59, N=5) or 3 pg *caAlk8* mRNA (n=44, N=4) (side views) at bud stage. Control explants correspond to explants shown in Fig. 2A. All embryos were co-injected with 50-100 pg *memRFP* or *memGFP* as injection control. (**E**) Percentage of extended/not-extended explants from wildtype embryos (control: n=236, N=13), and embryos injected with 37 pg *chrd-GFP* (n=45, N=4), 1 pg *bmp2b* (n=41, N=5), 3 pg *bmp2b* (n=59, N=5) or 3 pg *caAlk8* mRNA (n=44, N=4) at bud stage. Control explants partially correspond to explants shown in Fig. 2B. All embryos were co-injected with 50-100 pg *memRFP* or *memGFP* as injection control. (**F**) Circularity of extended/not-extended blastoderm explants from wildtype embryos (control: n=236, N=13), and embryos injected with 37 pg *chrd-GFP* (n=45, N=4), 1 pg *bmp2b* (n=41, N=5), 3 pg *bmp2b* (n=59, N=5) or 3 pg *caAlk8* mRNA (n=44, N=4) at bud stage. Control explants partially correspond to explants shown in Fig. 2C. All embryos were co-injected with

Development • Supplementary information

50-100 pg *memRFP* or *memGFP* as injection control. ****p<0.0001, ns, not significant (Kruskal-Wallis test). (**G**) Normalized extension length of extended blastoderm explants from wildtype embryos (control: n=210, N=13), and embryos injected with 37 pg *chrd-GFP* (n=41, N=4), 1 pg *bmp2b* (n=29, N=5), 3 pg *bmp2b* (n=29, N=5) or 3 pg *caAlk8* mRNA (n=21, N=4) at bud stage. Control explants partially correspond to explants shown in Fig. 2D. All embryos were co-injected with 50-100 pg *memRFP* or *memGFP* as injection control. ****p<0.0001, ns, not significant (Kruskal-Wallis test). Scale bars: 250 μm (**C**), 500 μm (**D**).

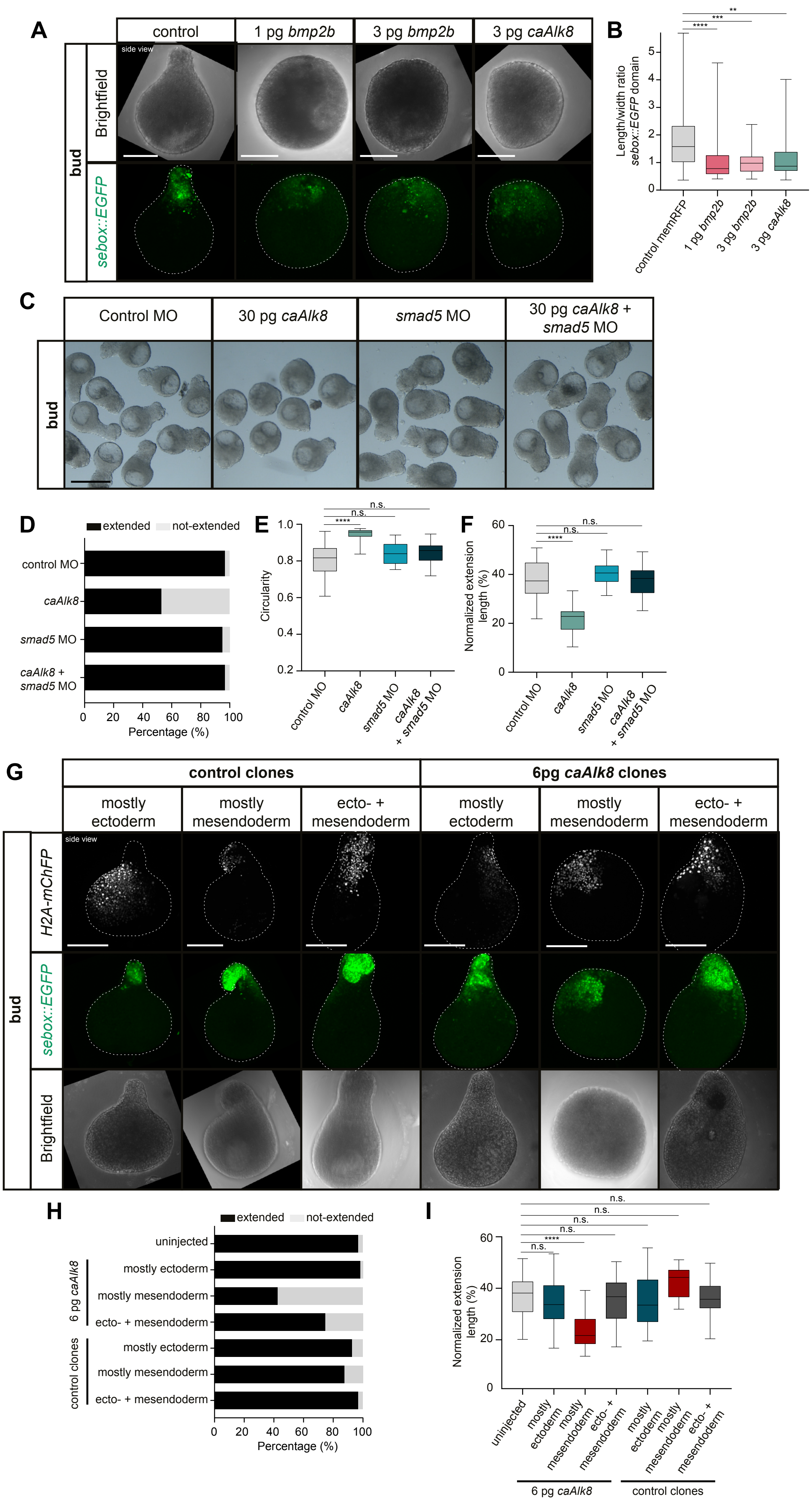
A
control
1 pg bmp2b
3 pg bmp2b
3 pg caAlk8
bud
Brightfield
side view
sebox::EGFP
B
Length/width ratio sebox::EGFP domain
**
***
****
control memRFP
1 pg bmp2b
3 pg bmp2b
3 pg caAlk8
C
Control MO
30 pg caAlk8
smad5 MO
30 pg caAlk8 + smad5 MO
bud
D
extended
not-extended
control MO
caAlk8
smad5 MO
caAlk8 + smad5 MO
Percentage (%)
E
Circularity
n.s.
****
control MO
caAlk8
smad5 MO
caAlk8 + smad5 MO
F
Normalized extension length (%)
n.s.
****
control MO
caAlk8
smad5 MO
caAlk8 + smad5 MO
G
control clones
6pg caAlk8 clones
mostly ectoderm
mostly mesendoderm
ecto- + mesendoderm
bud
H2A-mChFP
side view
sebox::EGFP
Brightfield
H
extended
not-extended
uninjected
6 pg caAlk8
control clones
mostly ectoderm
mostly mesendoderm
ecto- + mesendoderm
Percentage (%)
I
Normalized extension length (%)
n.s.
****
uninjected
mostly ectoderm
mostly mesendoderm
ecto- + mesendoderm
6 pg caAlk8
control clones

Fig. S3. Changes in blastoderm explant length upon BMP signaling overactivation. (**A**) Maximum intensity projection of fluorescence images (side views) of blastoderm explants from Tg(*sebox::EGFP*) wildtype embryos expressing EGFP (green) in mesendoderm progenitors (control: n=62, N=8) and Tg(*sebox::EGFP*) embryos injected with 1 pg *bmp2b* (n=31, N=6), 3 pg *bmp2b* (n=25, N=6) and 3 pg *caAlk8* (n=21, N=4) at bud stage. Control explants correspond to explants shown in Fig. 2E. All embryos were co-injected with 50-100 pg *memRFP* as injection control. (**B**) Length/width ratio of the EGFP expression domain in blastoderm explants from Tg(*sebox::EGFP*) wildtype embryos marking mesendoderm progenitors (control: n=62, N=8) and Tg(*sebox::EGFP*) embryos injected with 1 pg *bmp2b* (n=31, N=6), 3 pg *bmp2b* (n=25, N=6) and 3 pg *caAlk8* (n=21, N=4) at bud stage. Control explants correspond to explants shown in Fig. 2F. All embryos were co-injected with 50-100 pg *memRFP* as injection control. ****p<0.0001, ***p=0.0008, **p=0.0032 (Kruskal-Wallis test). (**C**) Single-plane bright-field images of blastoderm explants from wildtype embryos (controlMO: n=31, N=3) and embryos injected with 30 pg *caAlk8* (n=36, N=3), *smad5*MO (n=20, N=3) and 30 pg *caAlk8* + *smad5*MO (n=29, N=3) (side views) at bud stage. All embryos were co-injected with 50-100 pg *memRFP* or *memGFP* as injection control. (**D**) Percentage of extended/not-extended blastoderm explants from wildtype embryos (controlMO: n=31, N=3) and embryos injected with 30 pg *caAlk8* (n=36, N=3), *smad5*MO (n=20, N=3) and 30pg *caAlk8* + *smad5*MO (n=29, N=3) at bud stage. All embryos were co-injected with 50-100pg *memRFP* or *memGFP* as injection control. (**E**) Circularity of extended or not-extended blastoderm explants from wildtype embryos (controlMO: n=31, N=3) and embryos injected with 30 pg *caAlk8* (n=36, N=3), *smad5*MO (n=20, N=3) and 30 pg *caAlk8* + *smad5*MO (n=29, N=3) at bud stage. All embryos were co-injected with 50-100 pg *memRFP* or *memGFP* as injection control. ****p<0.0001, ns, not significant (Kruskal-Wallis test). (**F**) Normalized extension length of extended blastoderm explants from wildtype embryos (controlMO: n=30, N=3) and embryos injected with 30 pg *caAlk8* (n=18, N=3), *smad5*MO (n=19, N=3) and 30 pg *caAlk8* + *smad5*MO (n=29, N=3) at bud stage. All embryos were co-injected with 50-100 pg *memRFP* or *memGFP* as injection control. ****p<0.0001, ns, not significant (One-way ANOVA). (**G**) Maximum intensity projection of fluorescence images (side views) of blastoderm explants obtained from Tg(*sebox::EGFP*) embryos expressing EGFP (green) in mesendoderm progenitors at bud stage showing clonally labeled cell nuclei of control blastomere injections (5 pg *H2A-mChFP* mRNA injected) or

Development • Supplementary information

blastomere injections with 6 pg *caAlk8* and 5 pg *H2A-mChFP* mRNA (grey) positioned either mostly in the ectoderm (control: n=27, N=4; 6 pg *caAlk8*: n=57, N=4), mostly in the mesendoderm (control: n=8, N=4; 6 pg *caAlk8*: n=33, N=4) or in both the ectoderm and mesendoderm (control: n=30, N=4; 6 pg *caAlk8*: n=47, N=4). (**H**) Percentage of extended/not-extended blastoderm explants obtained from uninjected wildtype embryos (n=60, N=4), single blastomere injected embryos with 5 pg *H2A-mChFP* (positioned mostly in the ectoderm: n=27, N=4; mostly in the mesendoderm: n=8, N=4; in both the ectoderm and mesendoderm: n=30, N=4) and single blastomere injected embryos with 5 pg *H2A-mChFP* + 6 pg *caAlk8* positioned mostly in ectoderm: n=57, N=4; mostly in mesendoderm: n=33, N=4; in ectoderm and mesendoderm: n=47, N=4) blastoderm explants at bud stage. (**I**) Normalized extension length of extended blastoderm explants obtained from uninjected wildtype embryos (n=58, N=4), single blastomere injected embryos with 5 pg *H2A-mChFP* (positioned mostly in ectoderm: n=25, N=4; mostly in mesendoderm: n=7, N=4; in ectoderm and mesendoderm: n=29, N=4) and single blastomere injected embryos with 5 pg *H2A-mChFP* + 6 pg *caAlk8* (mostly in ectoderm: n=56, N=4; mostly in mesendoderm: n=14, N=4; in ectoderm and mesendoderm: n=35, N=4) at bud stage. ****$p<0.0001$, ns, not significant (One-way ANOVA). Scale bars: 200 µm (**A,G**), 500 µm (**C**).

Development • Supplementary information

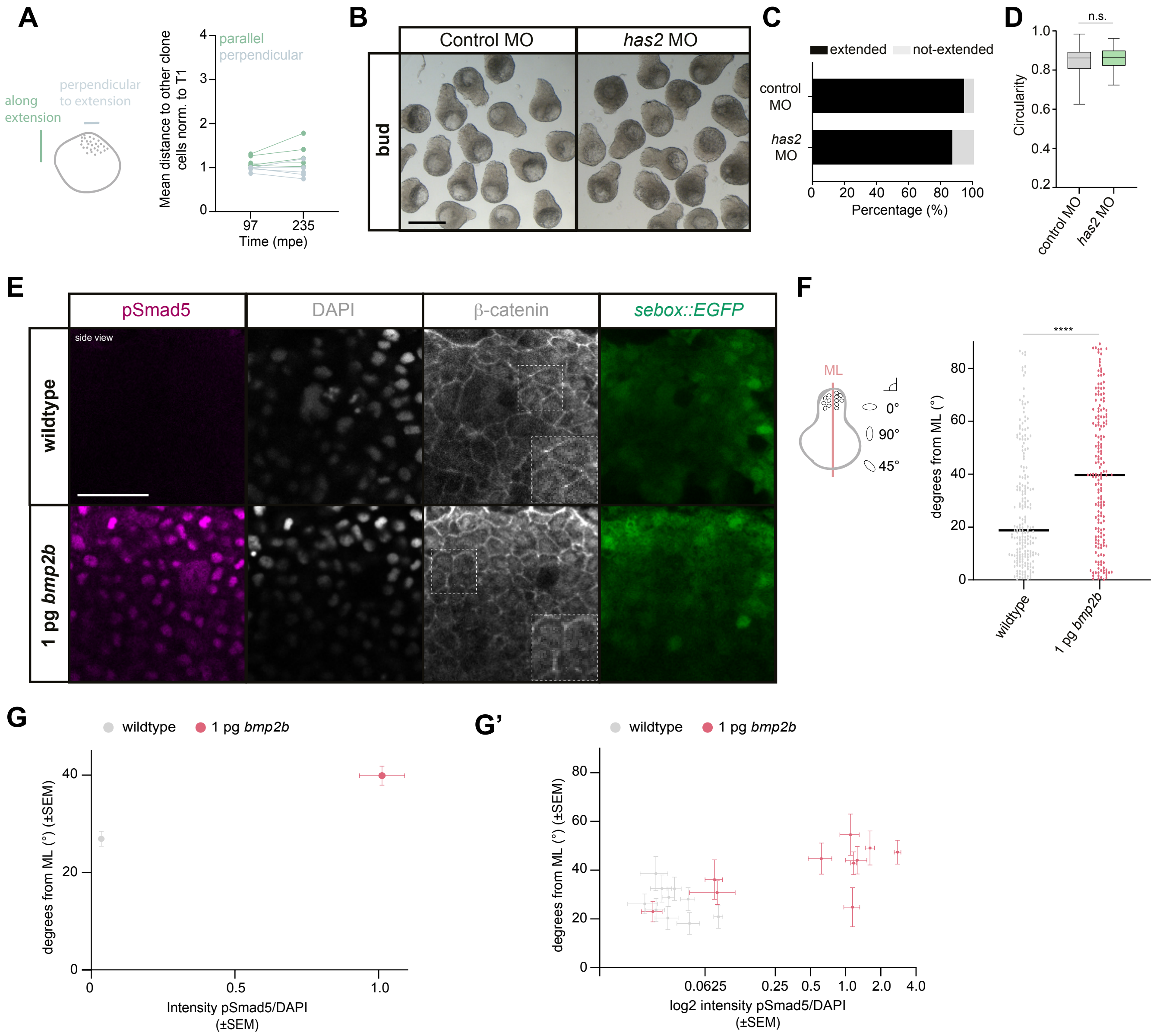
A
along extension
perpendicular to extension
parallel
perpendicular
Mean distance to other clone cells norm. to T1
Time (mpe)
B
Control MO
has2 MO
bud
C
extended
not-extended
control MO
has2 MO
Percentage (%)
D
n.s.
Circularity
control MO
has2 MO
E
pSmad5
DAPI
β-catenin
sebox::EGFP
side view
wildtype
1 pg bmp2b
F
ML
0°
90°
45°
****
degrees from ML (°)
wildtype
1 pg bmp2b
G
wildtype
1 pg bmp2b
degrees from ML (°) (±SEM)
Intensity pSmad5/DAPI (±SEM)
G'
wildtype
1 pg bmp2b
degrees from ML (°) (±SEM)
log2 intensity pSmad5/DAPI (±SEM)

Fig. S4. Elongation of blastoderm explants upon *has2* knock-down. (**A**) Clone dispersal parallel (dark green) and perpendicular (light green) to the axis of explant elongation assessed by the mean distance of each cell in the clone to other clone cells at two timepoints during explant elongation (97 mpe and 235 mpe) for each individual blastoderm explant from 30 pg *caAlk8* injected embryos. (**B**) Single-plane bright-field images (side views) of blastoderm explants from wildtype embryos (controlMO: n=102, N=6) and embryos injected with *has2*MO (n=91, N=6) at bud stage. All embryos were co-injected with 50-100 pg *memRFP* or *memGFP* as injection control. (**C**) Percentage of extended/not-extended blastoderm explants from wildtype embryos (controlMO: n=102, N=6) and embryos injected with *has2*MO (n=91, N=6) at bud stage. All embryos were co-injected with 50-100 pg *memRFP* or *memGFP* as injection control. (**D**) Circularity of extended or not-extended blastoderm explants from wildtype embryos (controlMO n=102, N=6) and embryos injected with *has2*MO (n=91, N=6) at bud stage. All embryos were co-injected with 50-100pg *memRFP* or *memGFP* as injection control. ns, not significant (Mann-Whitney test). (**E**) Single-plane high-resolution images (side views) of blastoderm explants obtained from Tg(*sebox::EGFP*) embryos expressing EGFP (green) in mesendoderm progenitors, uninjected (control) or injected with 1 pg *bmp2b* during explant elongation (corresponding to embryonic 90% epiboly stage) stained for pSmad5 (BMP signaling activity; magenta), β-catenin (cell outlines; grey) and DAPI (nuclei; grey) (wildtype: n=10, N=2; 1 pg *bmp2b*: n=10, N=2). (**F**) Cell alignment assessed by the deviation (degrees) of the main cell extension axis from the main mediolateral (ML) explant axis during elongation for the explants described in (**E**) (90% epiboly: wildtype: 205 cells, n=10, N=2; 1 pg *bmp2b*: 176 cells, n=10, N=2). ****$p<0.0001$ (Mann-Whitney test). (**G**) Cell alignment assessed by the deviation (degrees) of the main cell extension axis from the main mediolateral (ML) explant axis during explant elongation (corresponding to embryonic 90% epiboly stage) versus pSmad5 intensity normalized to DAPI for the explants described in (**E**) (wildtype: n=10, N=2; 1 pg *bmp2b*: n=10, N=2). (**G’**) Cell alignment assessed by the deviation (degrees) of the main cell extension axis from the main mediolateral (ML) explant axis during explant elongation (corresponding to embryonic 90% epiboly stage) versus pSmad5 intensity normalized to DAPI for the explants described in (**E**) (wildtype: n=10, N=2; 1 pg *bmp2b*: n=10, N=2) shown for individual explants. Scale bar: 500 µm (**B**), 50 µm (**E**).

Development • Supplementary information

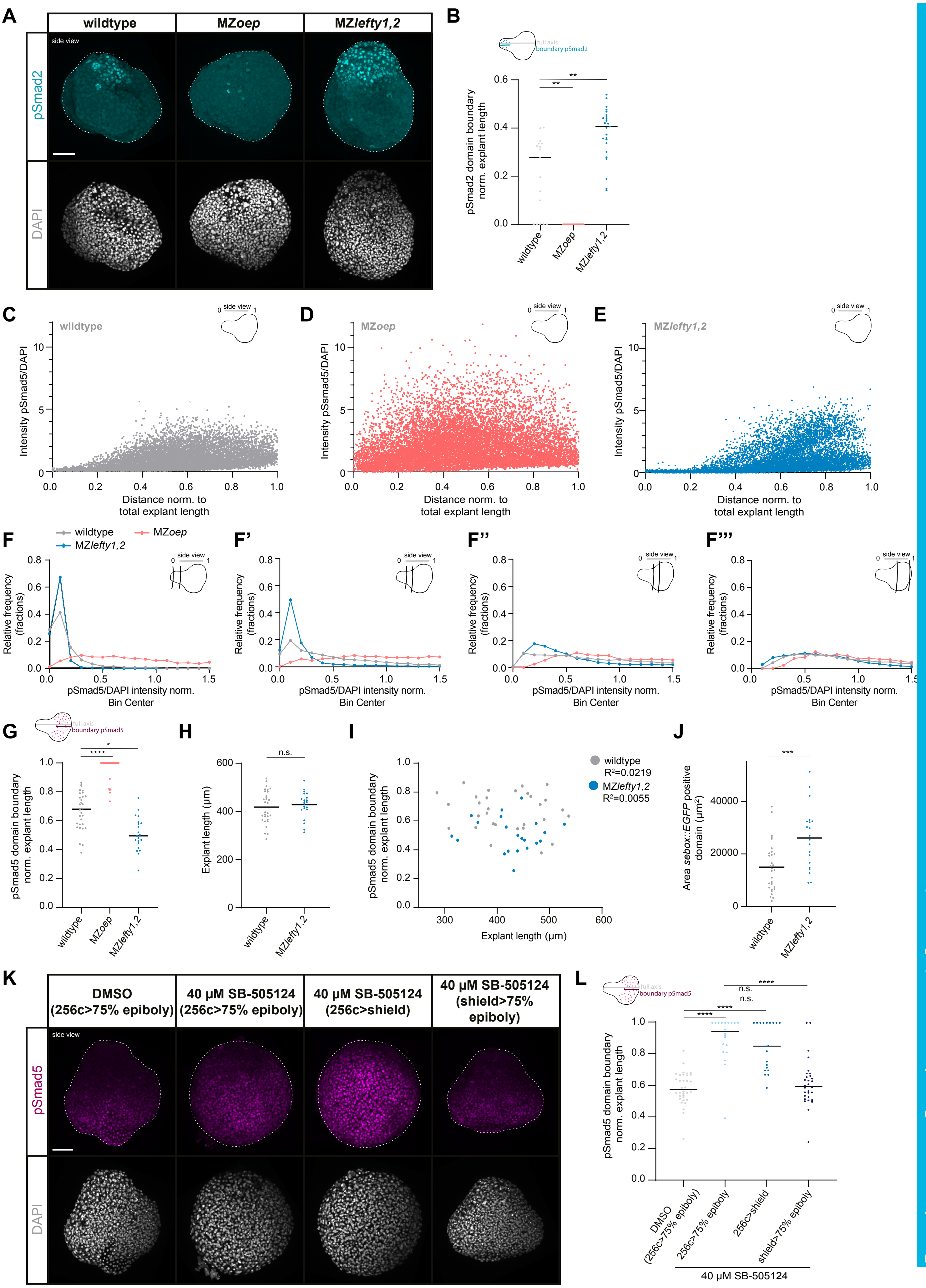

Development • Supplementary information

Fig. S5. Profile of pSmad5 distribution upon perturbation of Nodal signaling. (**A**) Maximum intensity projection of fluorescence images (side views) of blastoderm explants from wildtype, MZ*oep* and MZ*lefty1,2* embryos during explant elongation (corresponding to embryonic 75% epiboly stage) stained for pSmad2 (Nodal signaling activity; cyan) and DAPI (nuclei; grey) (wildtype: n=17, N=3; MZ*oep*: n=22, N=3; MZ*lefty1,2*: n=25, N=3). (**B**) Domain extent of nuclear pSmad2 measured along a line from the tip of the explant through its center to the back of the domain normalized to the explant length in blastoderm explants obtained from wildtype embryos (n=17, N=3), MZ*oep* embryos (n=22, N=3) and MZ*lefty1,2* embryos (n=25, N=3) during explant elongation (corresponding to embryonic 75% epiboly stage) with the black line indicating the median. **p=0.0066 (wildtype, MZ*oep*), **p=0.0053 (wildtype, MZ*lefty1,2*) (Kruskal-Wallis test). (**C**) Intensity of nuclear pSmad5 normalized to DAPI as a function of the distance from the explant tip in blastoderm explants from wildtype embryos (n=9, N=4) during explant elongation (corresponding to embryonic 75% epiboly stage). (**D**) Intensity of nuclear pSmad5 normalized to DAPI as a function of the distance from the explant tip in blastoderm explants from MZ*oep* embryos (n=9, N=4) during explant elongation (corresponding to embryonic 75% epiboly stage). (**E**) Intensity of nuclear pSmad5 normalized to DAPI as a function of the distance from the explant tip in blastoderm explants from MZlefty*1,2* embryos (n=9, N=4) during explant elongation (corresponding to embryonic 75% epiboly stage). (**F-F'''**) Frequency distribution of pSmad5/DAPI ratio relative to the mean intensity in the bin closest to the back (high intensity domain) of the wildtype explants, as determined in Fig. 4F, in discrete spatial bins across the tip-back axis for blastoderm explants from wildtype (grey), MZ*oep* (salmon) and MZ*lefty1,2* (blue) mutant embryos during explant elongation (corresponding to embryonic 75% epiboly stage). Notably the first and last 4% of the explant were excluded due to low number of nuclei at the ends. The spatial bins are thus shown as (F) 0.04-0.25 from the tip, (F') 0.25-0.5 from the tip, (F'') 0.5-0.75 from the tip, (F''') 0.75-0.96 from the tip. (**G**) Domain extent of nuclear pSmad5 measured along a line from the back of the explant through its center to the start of the domain normalized to the explant length in blastoderm explants obtained from wildtype embryos (n=30, N=5), MZ*oep* embryos (n=31, N=5) and MZ*lefty1,2* embryos (n=21, N=5) during explant elongation (corresponding to embryonic 75% epiboly stage) with the black line indicating the median. ****p<0.0001, *p=0.028 (Kruskal-Wallis test). (**H**) Length of blastoderm explants obtained from Tg(*sebox::EGFP*)

Development • Supplementary information

embryos (n=30, N=5) and MZ*lefty1,2*;Tg(*sebox::EGFP*) embryos (n=21, N=5) during explant elongation (corresponding to embryonic 75% epiboly stage) with the black line indicating the mean. ns, not significant (Unpaired t-test). (**I**) Relationship between the length of blastoderm explants obtained from Tg(*sebox::EGFP*) embryos (n=30, N=5) and MZ*lefty1,2*; Tg(*sebox::EGFP*) embryos (n=21, N=5) during explant elongation (corresponding to embryonic 75% epiboly stage) and the pSmad5 domain extent normalized to the whole explant length. (**J**) Area of the EGFP expression domain marking mesendodermal progenitors normalized to the overall explant area in blastoderm explants obtained from Tg(*sebox::EGFP*) embryos (n=30, N=5) and MZ*lefty1,2*;Tg(*sebox::EGFP*) embryos (n=20, N=5) during explant elongation (corresponding to embryonic 75% epiboly stage) with the black line indicating the mean. ***p=0.0006 (Unpaired t-test). (**K**) Maximum intensity projection of fluorescence images (side views) of blastoderm explants obtained from Tg(*sebox::EGFP*) embryos treated with DMSO (treated from 256c to 75% epiboly: n=34, N=6) or Nodal inhibitor (40 µM SB-505124; treated from 256c to 75% epiboly: n=20, N=6; treated from 256c to shield stage: n=20, N=6; treated from shield stage to 75% epiboly: n=30, N=6) during explant elongation (corresponding to embryonic 75% epiboly stage) stained for pSmad5 (BMP signaling activity; magenta) and DAPI (nuclei; grey). (**L**) Domain extent of nuclear pSmad5 measured along a line from the tip of the explant through its center to the back of the domain normalized to the explant length in blastoderm explants obtained from Tg(*sebox::EGFP*) embryos treated with DMSO (treated from 256c to 75% epiboly: n=34, N=6) or Nodal inhibitor (40 µM SB-505124; treated from 256c to 75% epiboly: n=20, N=6; treated from 256c to shield stage: n=20, N=6; treated from shield stage to 75% epiboly: n=30, N=6) during explant elongation (corresponding to embryonic 75% epiboly stage) with the black line indicating the median. ****p<0.0001, ns, not significant (Kruskal-Wallis test). Scale bars: 100 µm (**A,K**)

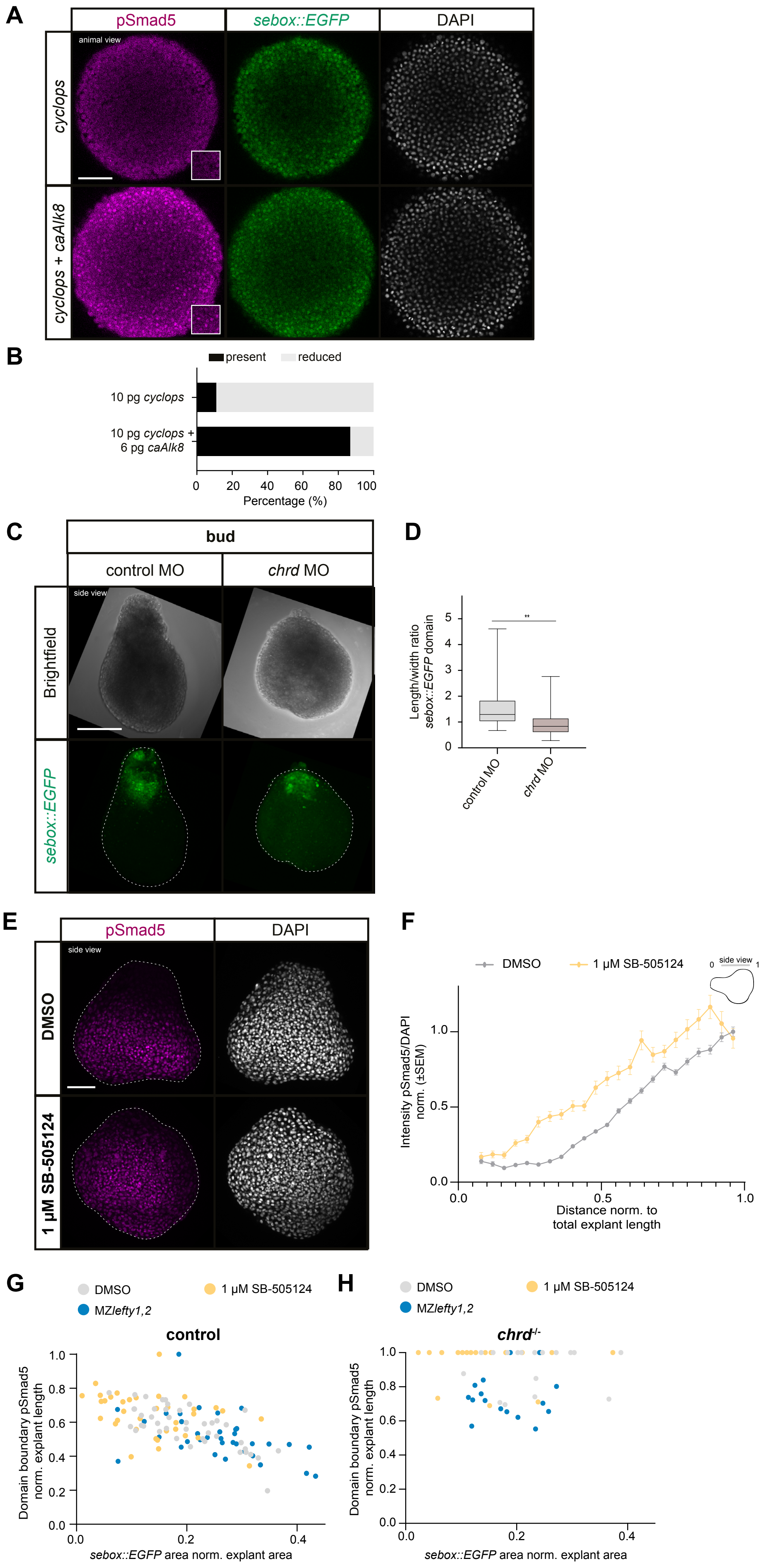

Development • Supplementary information

Fig. S6. Repression of BMP signaling activity by Nodal-dependent regulation of *chordin* expression. (**A**) Single-plane high-resolution images (animal view) of Tg(*sebox::EGFP*) embryos marking mesendoderm progenitors (green) at 75% epiboly stained for pSmad5 (BMP signaling activity; magenta) and DAPI (nuclei; grey) injected with 10 pg *cyclops* (top) (n=18, N=3) or 10 pg *cyclops* + 6 pg *caAlk8* mRNA (bottom) (n=15, N=3). (**B**) Percentage of present/absent nuclear pSmad5 in embryos injected with 10pg *cyclops* (n=18, N=3) or 10 pg *cyclops* + 6 pg *caAlk8* mRNA (n=15, N=3) at 75% epiboly stage. (**C**) Maximum intensity projection of fluorescence images (side views) of blastoderm explants from Tg(*sebox::EGFP*) embryos expressing EGFP (green) in mesendoderm progenitors (5 ng controlMO: n=26, N=3) and embryos injected with 4.5 ng *chrd*MO (n=17, N=3) at bud stage. All embryos were co-injected with 50-100pg *memRFP* as injection control. (**D**) Length/width ratio of the EGFP expression domain in blastoderm explants from Tg(*sebox::EGFP*) wildtype embryos marking mesendoderm progenitors (5 ng controlMO: n=26, N=3) and embryos injected with 4.5 ng *chrd*MO (n=17, N=3) at bud stage. All embryos were co-injected with 50-100 pg *memRFP* as injection control. **p=0.0013 (Mann-Whitney test). (**E**) Maximum intensity projection of fluorescence images (side views) of blastoderm explants obtained from Tg(*sebox::EGFP*) embryos treated with DMSO (treated from 256c to 75% epiboly: n=5, N=3) or Nodal inhibitor (1 μM SB-505124; treated from 256c to 75% epiboly: n=5, N=3) during explant elongation (corresponding to embryonic 75% epiboly stage) stained for pSmad5 (BMP signaling activity; magenta) and DAPI (nuclei; grey). (**F**) Intensity of nuclear pSmad5 normalized to DAPI as a function of the distance from the explant tip during elongation (corresponding to embryonic 75% epiboly stage) for the embryos described in (**E**) (DMSO treated from 256c to 75% epiboly: n=5, N=3; 1 μM SB-505124 treated from 256c to 75% epiboly: n=5, N=3). Intensities are shown relative to the mean intensity in the first bin closest to the back (high intensity domain) of the wildtype explants. 4% of the embryo at the sample edges were excluded due to low number of nuclei in these regions. Only explants, which had induced mesendoderm, as assessed by the expression of the mesendodermal marker *sebox::EGFP,* were analyzed. (**G**) Domain of nuclear pSmad5 measured along a line from the back of the explant through its center to the start of the domain normalized to the explant length, and area of *sebox::EGFP* expression, normalized to explant area, in blastoderm explants obtained from wildtype embryos treated with DMSO (treated from 256c to 75% epiboly: wildtype: n=43, N=6) or 1 μM Nodal inhibitor (SB-505124;

Development • Supplementary information

treated from 256c to 75% epiboly: wildtype: n=36, N=6) and MZ*lefty1,2* (n=35, N=5) embryos during explant elongation (corresponding to embryonic 75% epiboly stage) with Tg(*sebox::EGFP*) marking mesendoderm progenitors. (**H**) Domain of nuclear pSmad5 measured along a line from the back of the explant through its center to the start of the domain normalized to the explant length, and area of *sebox::EGFP* expression, normalized to explant area, in blastoderm explants obtained from *chrd*$^{-/-}$ embryos treated with DMSO (treated from 256c to 75% epiboly: *chrd*$^{-/-}$: n=17, N=4) or 1 µM Nodal inhibitor (SB-505124; treated from 256c to 75% epiboly: *chrd*$^{-/-}$: n=17, N=6) and MZ*lefty1,2;chrd*$^{-/-}$ (n=17, N=4) embryos during explant elongation (corresponding to embryonic 75% epiboly stage) with Tg(*sebox::EGFP*) marking mesendoderm progenitors. Scale bars: 100 µm (**A,E**), 200 µm (**C**).

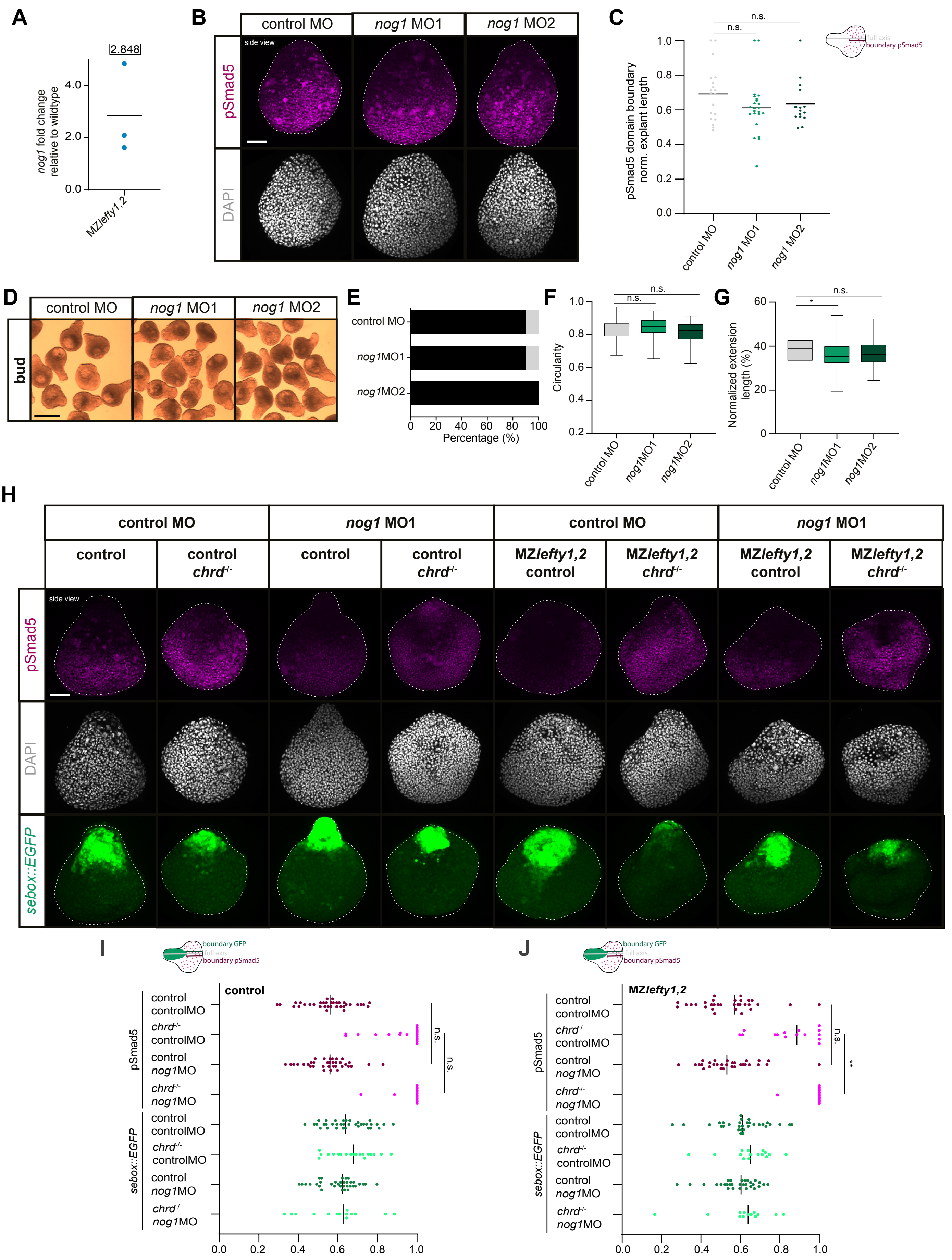
A
B
C
D
E
F
G
H
I
J
control MO
nog1 MO1
nog1 MO2
pSmad5
DAPI
side view
sebox::EGFP
bud
MZlefty1,2
chrd-/-
nog1 fold change relative to wildtype
2.848
pSmad5 domain boundary norm. explant length
Circularity
Normalized extension length (%)
Percentage (%)
n.s.
boundary GFP
full axis
boundary pSmad5
control
controlMO
nog1MO
**

Fig. S7. Nodal-activated *nog1* functions partially redundantly with *chrd* in repressing BMP signaling within the explant mesendoderm. (**A**) Fold change of *nog1* expression in MZ*lefty1,2* (N=3) explants relative to wildtype explants during explant elongation (corresponding to embryonic 75% epiboly stage). The black line indicates the mean. (**B**) Maximum intensity projection of fluorescence images (side views) of blastoderm explants obtained from wildtype embryos (4 ng control MO: n=19, N=3), embryos injected with 3 ng *nog1*-MO1 (n=23, N=3) and embryos injected with 4 ng *nog1*-MO2 (n=15, N=3) during explant elongation (corresponding to embryonic 75% epiboly stage) stained for pSmad5 (BMP signaling activity; magenta) and DAPI (nuclei; grey). (**C**) Domain of nuclear pSmad5 measured along a line from the back of the explant through its center to the end of the domain normalized to the explant length in blastoderm explants obtained from wildtype embryos (4 ng control MO: n=19, N=3), embryos injected with 3 ng *nog1*-MO1 (n=23, N=3) and embryos injected with 4 ng *nog1*-MO2 (n=15, N=3) during explant elongation (corresponding to embryonic 75% epiboly stage). ns, not significant (Kruskal-Wallis test) (**D**) Single-plane bright-field images (side views) of blastoderm explants obtained from wildtype embryos (4 ng control MO: n=58, N=4), embryos injected with 3 ng *nog1*-MO1 (n=52, N=4) and embryos injected with 4 ng *nog1*-MO2 (n=62, N=4) at bud stage. All embryos were co-injected with 80 pg *memGFP* as injection control. (**E**) Percentage of extended/not-extended blastoderm explants from wildtype embryos (4 ng control MO: n=58, N=4), embryos injected with 3 ng *nog1*-MO1 (n=52, N=4) and embryos injected with 4 ng *nog1*-MO2 (n=62, N=4) at bud stage. All embryos were co-injected with 80 pg *memGFP* as injection control. (**F**) Circularity of extended/not-extended blastoderm explants from wildtype embryos (4 ng control MO: n=58, N=4), embryos injected with 3 ng *nog1*-MO1 (n=52, N=4) and embryos injected with 4 ng *nog1*-MO2 (n=62, N=4) at bud stage. All embryos were co-injected with 80 pg *memGFP* as injection control. ns, not significant (Kruskal-Wallis test) (**G**) Normalized extension length of extended blastoderm explants from wildtype embryos (4 ng control MO: n=52, N=4), embryos injected with 3 ng *nog1*-MO1 (n=47, N=4) and embryos injected with 4 ng *nog1*-MO2 (n=62, N=4) at bud stage. All embryos were co-injected with 80 pg *memGFP* as injection control. *p=0.0338, ns, not significant (Kruskal-Wallis test) (**H**) Maximum intensity projection of fluorescence images (side views) of blastoderm explants obtained from 4 ng controlMO or 3 ng *nog1*-MO1 injected wildtype embryos (controlMO: n=35, N=4; *nog1*-MO1: n=35, N=4), *chrd*$^{-/-}$ (controlMO: n=19, N=4;

Development • Supplementary information

nog1-MO1: n=14, N=4), MZ*lefty1,2* (controlMO: n=31, N=5; *nog1*-MO1: n=30, N=5) and MZ*lefty1,2;chrd*$^{-/-}$ (controlMO: n=15, N=5, *nog1*-MO1: n=13, N=5) mutant Tg(*sebox::EGFP*) embryos marking mesendoderm progenitors (green) during explant elongation (corresponding to embryonic 75% epiboly stage) stained for pSmad5 (BMP signaling activity; magenta) and DAPI (nuclei; grey). (**I**) Domain of nuclear pSmad5 and EGFP expression measured along a line from the back of the explant through its center to the start of the domain normalized to the explant length in blastoderm explants obtained from 4 ng controlMO or 3 ng *nog1*-MO1 injected wildtype (controlMO: n=35, N=4; *nog1*-MO1: n=35, N=4) and *chrd*$^{-/-}$ mutant (controlMO: n=19, N=4; *nog1*-MO1: n=14, N=4) Tg(*sebox::EGFP*) embryos marking mesendoderm progenitors during explant elongation (corresponding to embryonic 75% epiboly stage). The black line indicates the median. ns, not significant (Unpaired t-test: wildtype controlMO/*nog1*MO; Mann-Whitney test: *chrd*$^{-/-}$ controlMO/*nog1*-MO). (**J**) Domain of nuclear pSmad5 and EGFP expression measured along a line from the back of the explant through its center to the start of the domain normalized to the explant length in blastoderm explants obtained from 4 ng controlMO or 3 ng *nog1*-MO1 injected MZ*lefty1,2* (controlMO: n=31, N=5; *nog1*-MO1: n=30, N=5) and MZ*lefty1,2;chrd*$^{-/-}$ (controlMO: n=15, N=5, *nog1*-MO1: n=13, N=5) mutant Tg(*sebox::EGFP*) embryos marking mesendoderm progenitors during explant elongation (corresponding to embryonic 75% epiboly stage). The black line indicates the median. **p=0.0023, ns, not significant (Mann-Whitney test). Scale bars: 100 µm (**B,H**), 500 µm (**D**).

Development • Supplementary information

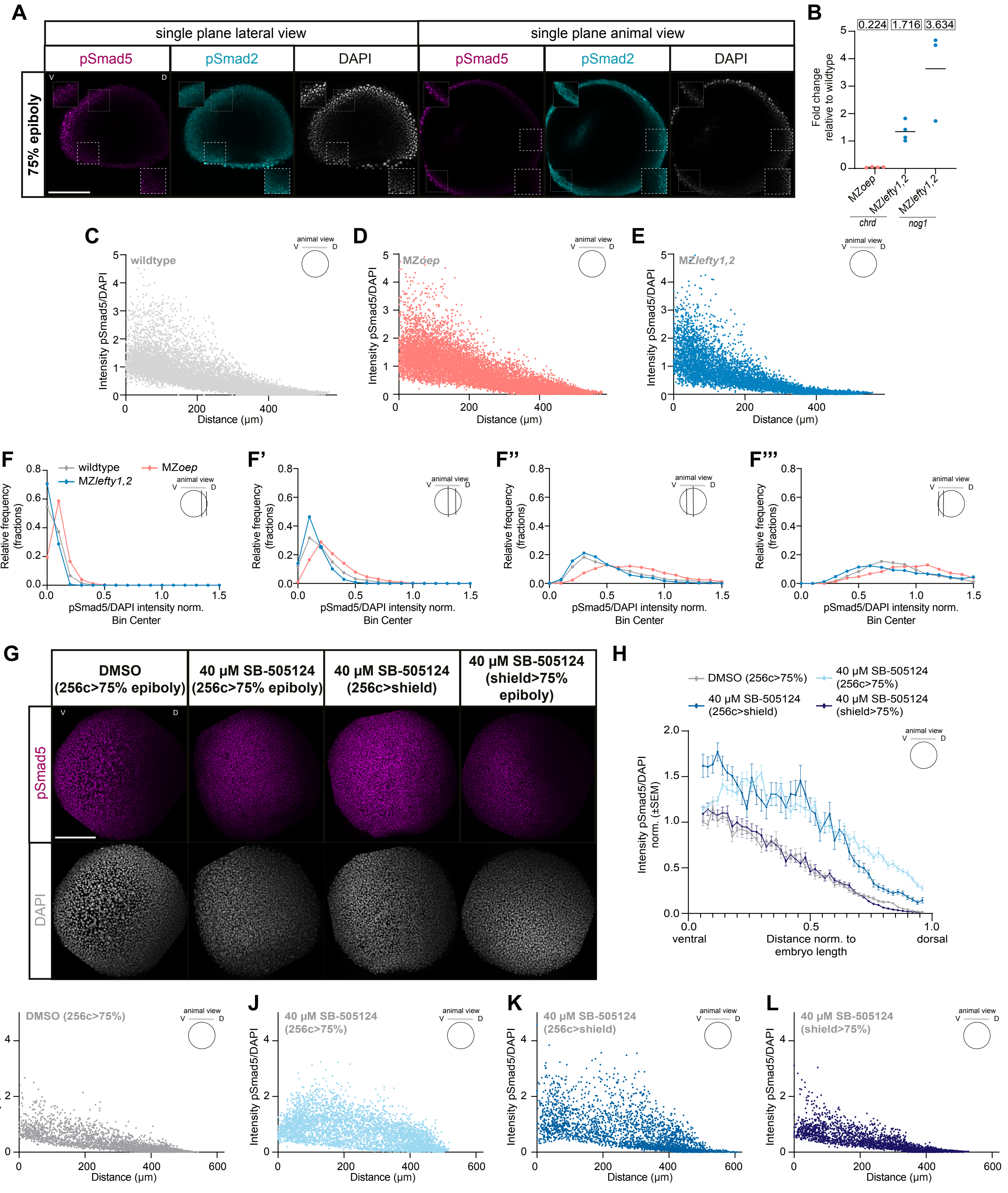

Development • Supplementary information

Fig. S8. Coordination of Nodal and BMP signaling domains in mid-gastrula stage zebrafish embryos. (**A**) Single-plane high-resolution images (lateral and animal views, respectively) of wildtype embryos at 75% epiboly stage stained for pSmad5 (BMP signaling activity; magenta), pSmad2 (Nodal signaling activity; cyan) and DAPI (nuclei; grey) (N=3). (**B**) Fold change of *chrd* expression in MZ*oep* (N=4) and MZ*lefty1,2* (N=4) embryos and of *nog1* expression in MZ*lefty1,2* (N=3) embryos relative to wildtype embryos at 75% epiboly stage. The black line indicates the mean. (**C**) Intensity of nuclear pSmad5 normalized to DAPI as a function of the distance from the ventral side in wildtype embryos (n=11, N=5) at 75% epiboly stage. D=dorsal, V=ventral. (**D**) Intensity of nuclear pSmad5 normalized to DAPI as a function of the distance from the ventral side in MZ*oep* embryos (n=11, N=5) at 75% epiboly stage. (**E**) Intensity of nuclear pSmad5 normalized to DAPI as a function of the distance from the ventral side in MZ*lefty1,2* embryos (n=11, N=5) at 75% epiboly stage. (**F-F'''**) Frequency distribution of pSmad5/DAPI ratio relative to the mean intensity in the bin closest to the ventral side (high intensity domain) of the wildtype embryos, as determined in Fig. 6B, in discrete spatial bins across the dorsoventral axis for wildtype (grey), MZ*oep* (salmon) and MZ*lefty1,2* (blue) mutant embryos respectively. Notably the first and last 4% of the embryo length were excluded due to low number of nuclei at the sample edges. The spatial bins are thus shown as (F) 0.04-0.25 from dorsal, (F') 0.25-0.5 from dorsal, (F'') 0.5-0.75 from dorsal, (F''') 0.75-0.96 from dorsal. (**G**) Maximum intensity projection of fluorescence images (animal views) of DMSO treated (treated from 4-16c to 75% epiboly: n=6, N=3) and Nodal inhibitor treated (40 μM SB-505124; treated from 4-16c to 75% epiboly: n=6, N=3; treated from 4-16c to shield stage: n=6, N=3; treated from shield stage to 75% epiboly: n=6, N=3) embryos at 75% epiboly stage stained for pSmad5 (BMP signaling activity; magenta) and DAPI (nuclei; grey). D=dorsal, V=ventral. (**H**) Intensity of nuclear pSmad5 normalized to DAPI as a function of the distance from the ventral side of 75% epiboly stage DMSO treated (from 4-16c to 75% epiboly: n=6, N=3) and 40 μM SB-505124 treated (from 4-16c to 75% epiboly: n=6, N=3; from 4-16c to shield stage: n=6, N=3; from shield stage to 75% epiboly: n=6, N=3) embryos. Intensities are shown relative to the mean intensity in the first bin closest to the ventral side (high intensity domain) of wildtype embryos. 4% of the embryo at the sample edges were excluded due to low number of nuclei in these regions. (**I**) Intensity of nuclear pSmad5 normalized to DAPI as a function of the distance from the ventral side in DMSO treated embryos (from 4-16c to 75% epiboly: n=6, N=3) at 75% epiboly stage. D=dorsal,

Development • Supplementary information

V=ventral. (**J**) Intensity of nuclear pSmad5 normalized to DAPI as a function of the distance from the ventral side in Nodal inhibitor treated embryos (40 μM SB-505124; treated from 4-16c to 75% epiboly: n=6, N=3) at 75% epiboly stage. (**K**) Intensity of nuclear pSmad5 normalized to DAPI as a function of the distance from the ventral side in Nodal inhibitor treated embryos (40 μM SB-505124; treated from 4-16c to shield: n=6, N=3) at 75% epiboly stage. (**L**) Intensity of nuclear pSmad5 normalized to DAPI as a function of the distance from the ventral side in Nodal inhibitor treated embryos (40 μM SB-505124; treated from shield to 75% epiboly: n=6, N=3) at 75% epiboly stage. Scale bars: 200 μm (**A**,**G**).

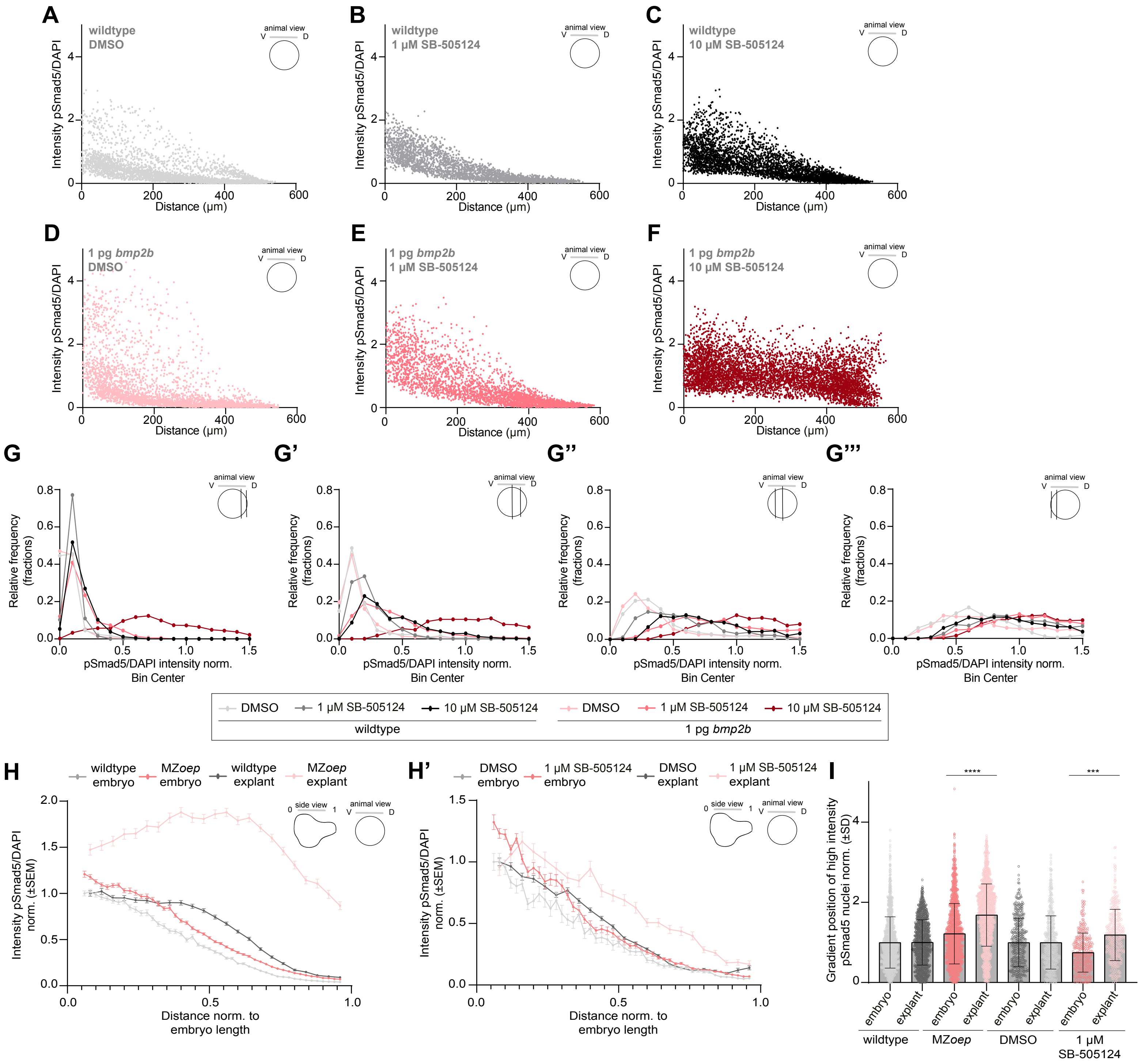

Development • Supplementary information

Fig. S9. pSmad5 activity upon concomitant Nodal and *bmp* perturbation. (**A**) Intensity of nuclear pSmad5 normalized to DAPI as a function of the distance from the ventral side in wildtype embryos treated with DMSO (treated from 4-16c to 75% epiboly: n=7, N=3) at 75% epiboly stage. D=dorsal, V=ventral. (**B**) Intensity of nuclear pSmad5 normalized to DAPI as a function of the distance from the ventral side in wildtype embryos treated with Nodal inhibitor (1 µM SB-505124; treated from 4-16c to 75% epiboly: n=7, N=3) at 75% epiboly stage. (**C**) Intensity of nuclear pSmad5 normalized to DAPI as a function of the distance from the ventral side in wildtype embryos treated with Nodal inhibitor (10 µM SB-505124; treated from 4-16c to 75% epiboly: n=7, N=3) at 75% epiboly stage. (**D**) Intensity of nuclear pSmad5 normalized to DAPI as a function of the distance from the ventral side in wildtype embryos injected with 1 pg *bmp2b* and treated with DMSO (treated from 4-16c to 75% epiboly: n=7, N=3) at 75% epiboly stage. (**E**) Intensity of nuclear pSmad5 normalized to DAPI as a function of the distance from the ventral side in wildtype embryos injected with 1 pg *bmp2b* and treated with Nodal inhibitor (1 µM SB-505124; treated from 4-16c to 75% epiboly: n=7, N=3) at 75% epiboly stage. (**F**) Intensity of nuclear pSmad5 normalized to DAPI as a function of the distance from the ventral side in wildtype embryos injected with 1 pg *bmp2b* and treated with Nodal inhibitor (10 µM SB-505124; treated from 4-16c to 75% epiboly: n=7, N=3) at 75% epiboly stage. All embryos in (**A**-**F**) were co-injected with 80-100 pg *memGFP* as injection control. (**G-G'''**) Frequency distribution of pSmad5/DAPI ratio relative to the mean intensity in the bin closest to the ventral side (high intensity domain) of wildtype embryos, as determined in Fig. 6E, in discrete spatial bins across the dorsoventral axis for DMSO treated (treated from 4-16c to 75% epiboly, control: n=7, N=3 (light grey); and injected with 1pg *bmp2b*: n=7, N=3 (light pink)), 1 µM SB-505124 treated (treated from 4-16c to 75% epiboly, control: n=7, N=3 (dark grey); and injected with 1pg *bmp2b*: n=7, N=3 (red)) and 10 µM SB-505124 (treated from 4-16c to 75% epiboly, control: n=7, N=3 (black); and injected with 1pg *bmp2b*: n=7, N=3 (dark red)) embryos. All embryos were co-injected with 80-100 pg *memGFP* as injection control. 4% of the embryo at the sample edges were excluded due to low number of nuclei in these regions. Consequently, the spatial bins are defined as (**G**) 0.04-0.25 from dorsal, (**G'**) 0.25-0.5 from dorsal, (**G''**) 0.5-0.75 from dorsal, (**G'''**) 0.75-0.96 from dorsal. (**H**) Intensity of nuclear pSmad5 normalized to DAPI as a function of the distance from the back of elongating explants (corresponding to embryonic 75% epiboly stage) and the ventral side of embryos (explants: wildtype:

Development • Supplementary information

n=9, N=4; MZ*oep*: n=9, N=4; embryos: wildtype: n=11, N=5; MZ*oep*: n=11, N=5). Intensities are shown relative to the mean intensity in the first bin closest to the back (high intensity domain) of the DMSO treated (control) explants and ventral side of the DMSO treated (control) embryos respectively. 4% of the embryo/explant at the sample edges were excluded due to low number of nuclei in these regions. The explant data corresponds to the data shown in Fig.4F. The embryo data corresponds to the data shown in Fig.6B. (**H'**) Intensity of nuclear pSmad5 normalized to DAPI as a function of the distance from the back of elongating explants (corresponding to embryonic 75% epiboly stage) and the ventral side of embryos (explants: DMSO treated from 256c to 75% epiboly: n=5, N=3; 1 µM SB-505124 treated from 256c to 75% epiboly: n=5, N=3; embryos: DMSO treated from 4-16c to 75% epiboly: n=7, N=3; 1 µM SB-505124 treated from 4-16c to 75% epiboly: n=7, N=3). Intensities are shown relative to the mean intensity in the first bin closest to the back (high intensity domain) of the DMSO treated (control) explants and ventral side of the DMSO treated (control) embryos respectively. 4% of the embryo at the sample edges were excluded due to low number of nuclei in these regions. The explant data corresponds to the data shown in Fig.S6F. The embryo data corresponds to the data shown in Fig.6E. Notably only explants and embryos, which contained mesendoderm, as assessed by the expression of the mesendodermal marker *sebox::EGFP*, were analyzed. (**I**) Relative position of top 10% of highest intensity pSmad5 positive nuclei from the ventral side of the embryo in wildtype (n=11, N=5) and MZ*oep* mutant (n=11, N=5) embryos relative to the mean position in wildtype embryos and in DMSO (control) treated (from 4-16c to 75% epiboly: n=7, N=3) and Nodal inhibitor treated (1 µM SB-505124 treated from 4-16c to 75% epiboly: n=7, N=3) embryos relative to the mean position in DMSO treated embryos. Relative position of top 10% of highest intensity pSmad5 positive nuclei from the back side of blastoderm explants in explants prepared from wildtype (n=9, N=4) and MZ*oep* mutant (n=9, N=4) embryos relative to the mean position in wildtype explants and in blastoderm explants prepared from DMSO (control) treated (from 256c to 75% epiboly: n=5, N=3) and Nodal inhibitor treated (1 µM SB-505124 treated from 256c to 75% epiboly: n=5, N=3) embryos relative to the mean position in DMSO treated explants. Error bars indicate the standard deviation. Notably only explants and embryos, which contained mesendoderm, as assessed by the expression of the mesendodermal marker *sebox::EGFP*, were analyzed. ****p<0.0001, ***p=0.0001 (Mann-Whitney test).

Development • Supplementary information

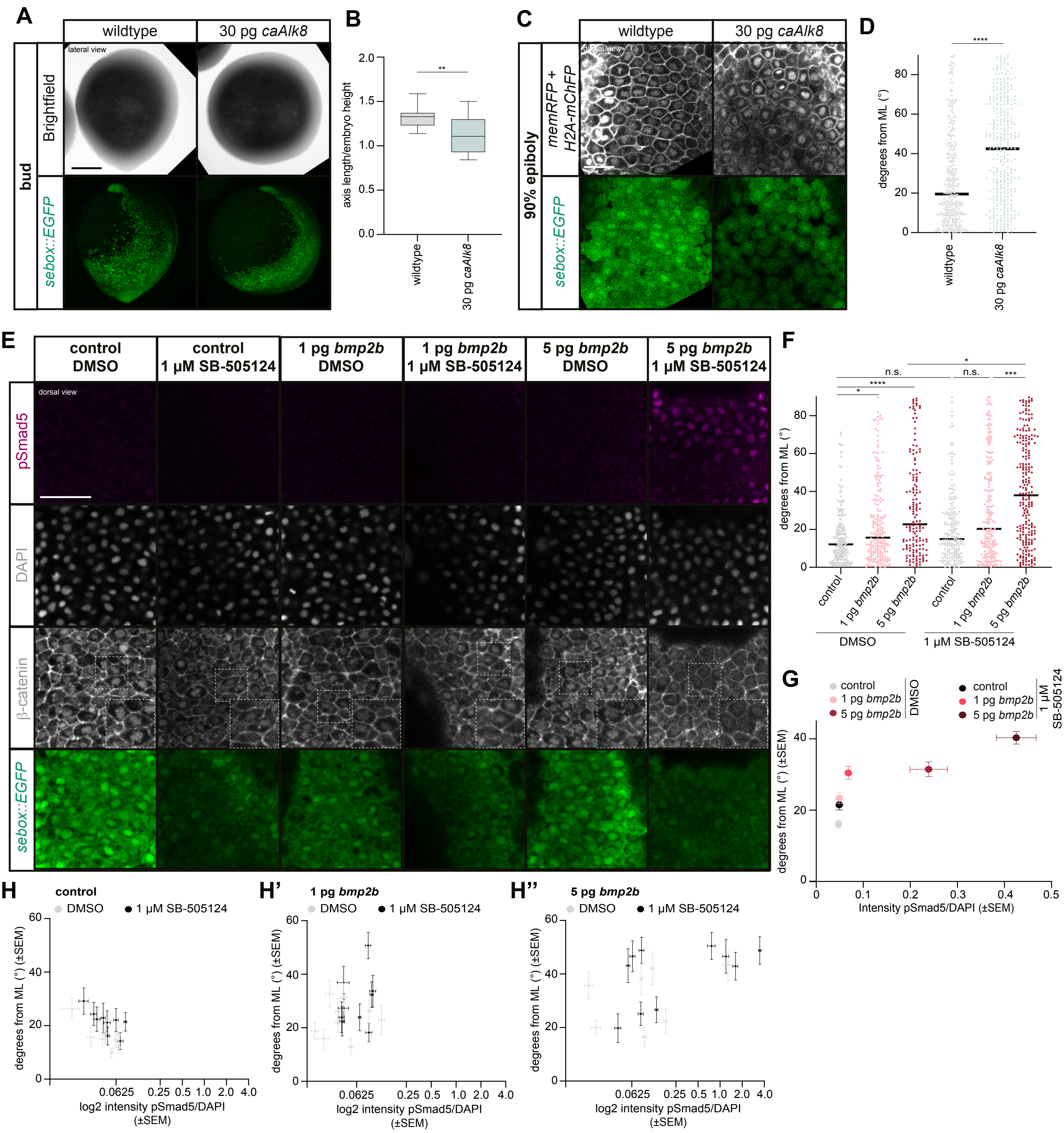

Development • Supplementary information

Fig. S10. Changes in axis elongation upon BMP signaling overactivation. (**A**) Maximum intensity projection of bright-field (top) and fluorescence (bottom) images (lateral views; dorsal pointing to the right) of Tg(*sebox::EGFP*) wildtype embryos (n=16, N=5) marking mesendodermal progenitors (left) or Tg(*sebox::EGFP*) embryos injected with 30 pg *caAlk8* (right) (n=17, N=5) at bud stage. All embryos were co-injected with 80 pg *memRFP* or 40 pg *H2A-mChFP* as injection control. (**B**) Length of the mesendodermal embryonic axis on the dorsal side over embryo height at bud stage in wildtype embryos (n=16, N=5) and embryos injected with 30 pg *caAlk8* mRNA (n=17, N=5). All embryos were co-injected with 80 pg *memRFP* or 40 pg *H2A-mChFP* as injection control. **p=0.0015 (Unpaired t-test). (**C**) Single-plane high-resolution images of wildtype and 30 pg *caAlk8* injected embryos (dorsal views) expressing Tg(*sebox::EGFP*) marking mesendodermal progenitors at 90% epiboly stage. Cell outlines are marked by 80-100 pg *memRFP* mRNA injection (grey) expression. Cell nuclei are marked by 40pg *H2A-mChFP* mRNA injection (grey) expression. (**D**) Cell alignment assessed by the deviation (degrees) of the main cell extension axis from the main mediolateral (ML) embryo axis during axis elongation at 90% epiboly (wildtype: 362 cells, n=7, N=4; 30pg *caAlk8*: 357 cells, n=7, N=4). ****p<0.0001 (Mann-Whitney test). (**E**) Single-plane high-resolution images (dorsal views) of DMSO treated (4-16c>75% epiboly, control: n=9, N=4; and injected with 1 pg *bmp2b*: n=9, N=4 or 5 pg *bmp2b*: n=7, N=4) or 1 μM SB-505124 treated (4-16c>75% epiboly, control: n=9, N=4; and injected with 1 pg *bmp2b*: n=9, N=4 or 5 pg *bmp2b*: n=10, N=4) 90% epiboly stage Tg(*sebox::EGFP*) embryos (marking mesendodermal progenitors in green) and stained for pSmad5 (BMP signaling activity; magenta), β-catenin (cell outlines; grey) and DAPI (nuclei; grey). (**F**) Cell alignment assessed by the deviation (degrees) of the main cell extension axis from the mediolateral (ML) axis in 90% epiboly stage embryos (DMSO treated: 4-16c>90% epiboly, control: n=9, N=4; and injected with 1 pg *bmp2b*: n=9, N=4 or 5 pg *bmp2b*: n=7, N=4; 1 μM SB-505124 treated: 4-16c>90% epiboly, control: n=9, N=4; and injected with 1 pg *bmp2b*: n=9, N=4 or 5pg *bmp2b*: n=10, N=4). *p=0.0425 (DMSO versus DMSO + 1 pg *bmp2b*), *p=0.0301 (DMSO + 5 pg *bmp2b* versus 1 μM SB-505124 + 5 pg *bmp2b*), ***p=0.0005, ****p<0.0001, ns, not significant (Kruskal-Wallis test). (**G**) Cell alignment assessed by the deviation (degrees) of the main cell extension axis from the mediolateral (ML) axis and pSmad5 intensity normalized to DAPI in DMSO treated (4-16c>90% epiboly, control: n=9, N=4; and

Development • Supplementary information

injected with 1pg *bmp2b*: n=9, N=4 or 5pg *bmp2b*: n=7, N=4) or 1 µM SB-505124 treated (4-16c>90% epiboly, control: n=9, N=4; and injected with 1pg *bmp2b*: n=9, N=4 or 5pg *bmp2b*: n=10, N=4) embryos at 90% epiboly. (**H**) Cell alignment assessed by the deviation (degrees) of the main cell extension axis from the mediolateral (ML) axis and pSmad5 intensity normalized to DAPI in DMSO treated (4-16c>90% epiboly, control: n=9, N=4) or 1 µM SB-505124 treated (4-16c>90% epiboly, control: n=9, N=4) embryos at 90% epiboly shown for individual explants. (**H'**) Cell alignment assessed by the deviation (degrees) of the main cell extension axis from the mediolateral (ML) axis and pSmad5 intensity normalized to DAPI in DMSO treated (4-16c>90% epiboly, injected with 1pg *bmp2b*: n=9, N=4) or 1 µM SB-505124 treated (4-16c>90% epiboly, injected with 1pg *bmp2b*: n=9, N=4) embryos at 90% epiboly shown for individual explants. (**H''**) Cell alignment assessed by the deviation (degrees) of the main cell extension axis from the mediolateral (ML) axis and pSmad5 intensity normalized to DAPI in DMSO treated (4-16c>90% epiboly, injected with 5pg *bmp2b*: n=7, N=4) or 1 µM SB-505124 treated (4-16c>90% epiboly, injected with 5pg *bmp2b*: n=10, N=4) embryos at 90% epiboly shown for individual explants. Scale bars: 200 µm (**A**), 100 µm (**C**), 50 µm (**E**).

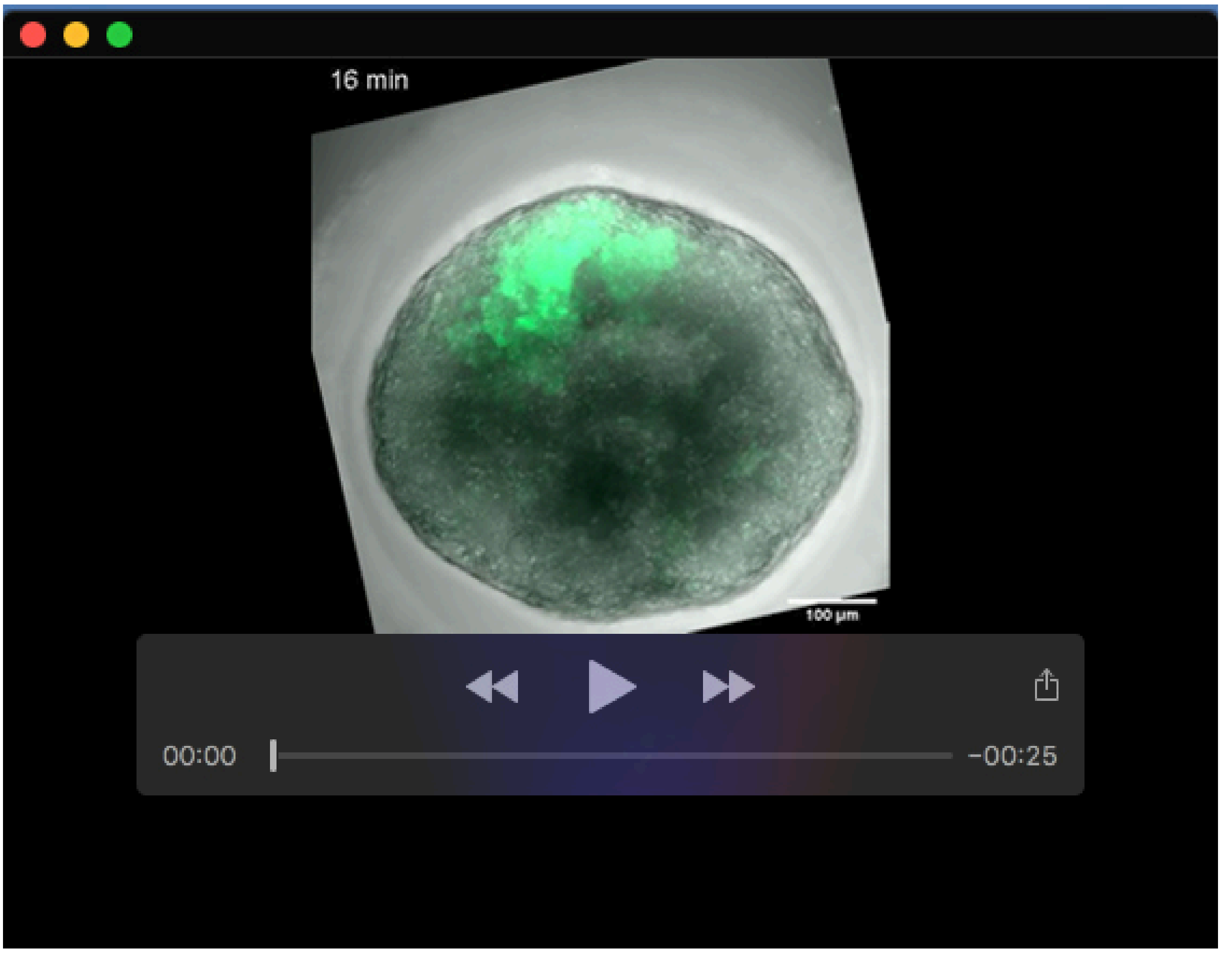

Movie 1. Blastoderm and mesendoderm morphogenesis in wildtype explants. Maximum intensity projection of fluorescence/brightfield high-resolution time-lapse imaging of blastoderm explants obtained from Tg(*sebox::EGFP*) embryos expressing EGFP (green) in mesendoderm progenitors from 24 minutes before the onset of extension (mbe). Time is shown in minutes. Scale bar: 100 µm.

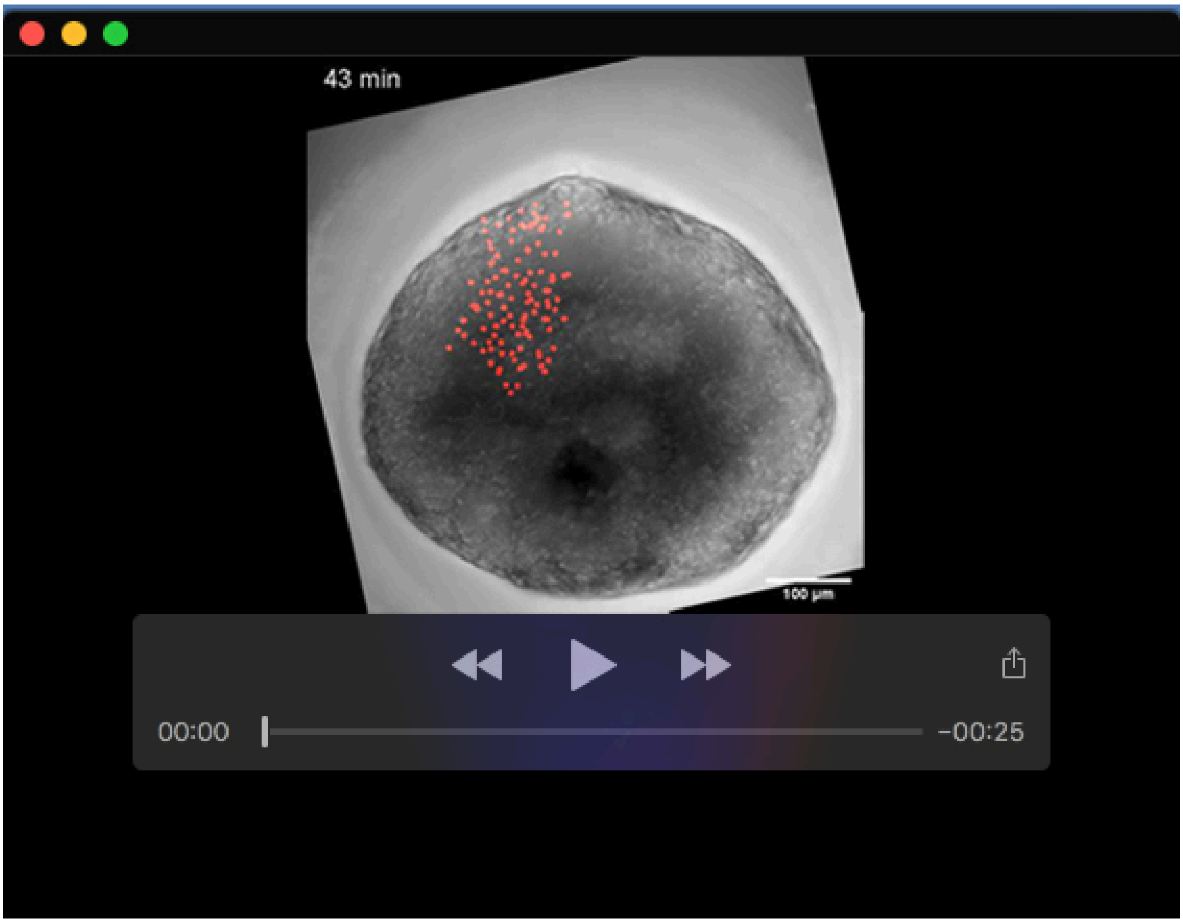

Movie 2. Mesendodermal clone dispersal in wildtype explants. Maximum intensity projection of fluorescence/brightfield high-resolution time-lapse imaging of blastoderm explants showing tracked clonally labeled cell nuclei in the mesendoderm (red) from 24 minutes before the onset of extension (mbe). Spots corresponding to individual tracked mesendodermal progenitors color-coded based on the individual track ID and corresponding cell trajectories generated in Imaris from 24 minutes before the onset of extension (mbe). Time is shown in minutes. Scale bar: 100 µm.

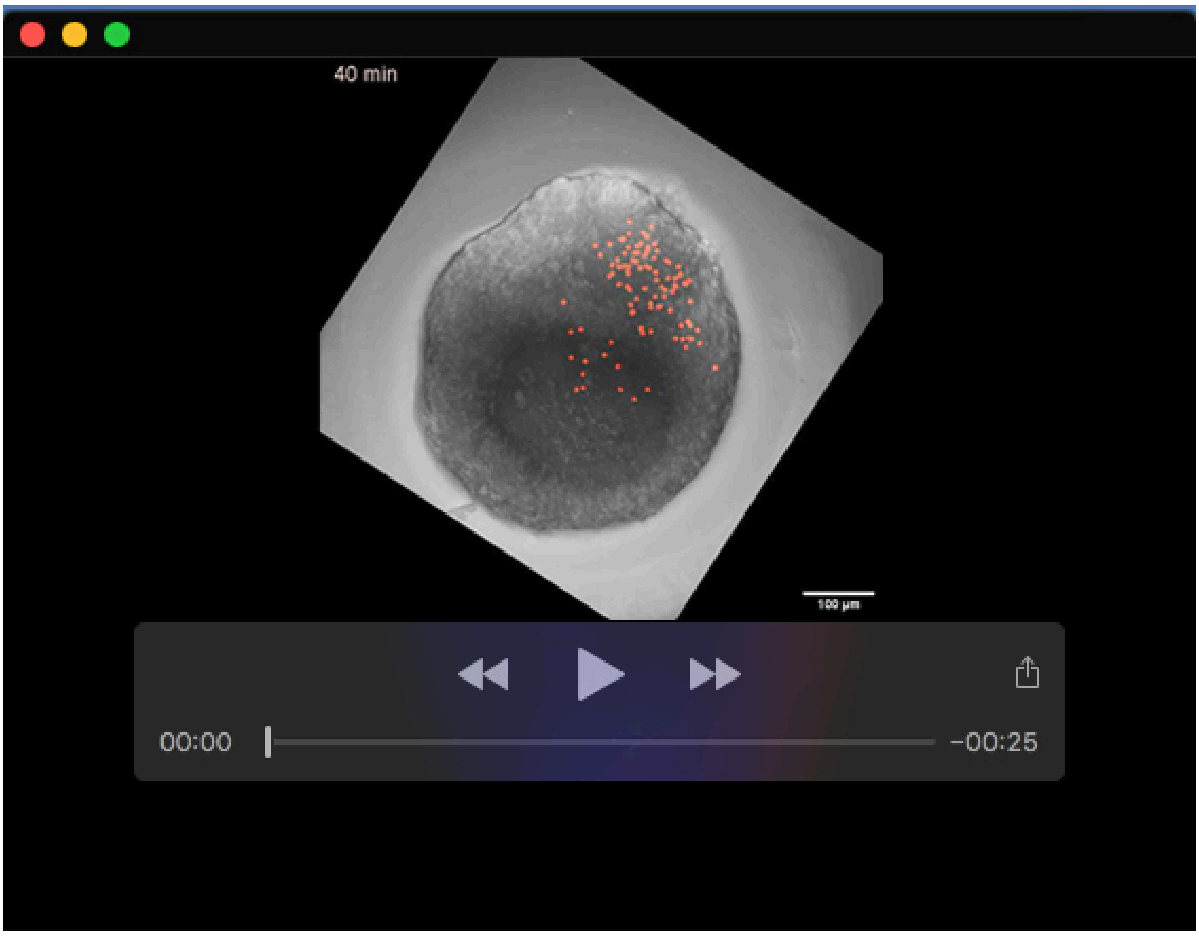

Movie 3. Mesendodermal clone dispersal in wildtype explants. Maximum intensity projection of fluorescence/brightfield high-resolution time-lapse imaging of blastoderm explants showing tracked clonally labeled cell nuclei in the mesendoderm (red) from 24 minutes before the onset of extension (mbe). Spots corresponding to individual tracked mesendodermal progenitors color-coded based on the individual track ID and corresponding cell trajectories generated in Imaris from 24 minutes before the onset of extension (mbe). Time is shown in minutes. Scale bar: 100 µm.

Development • Supplementary information

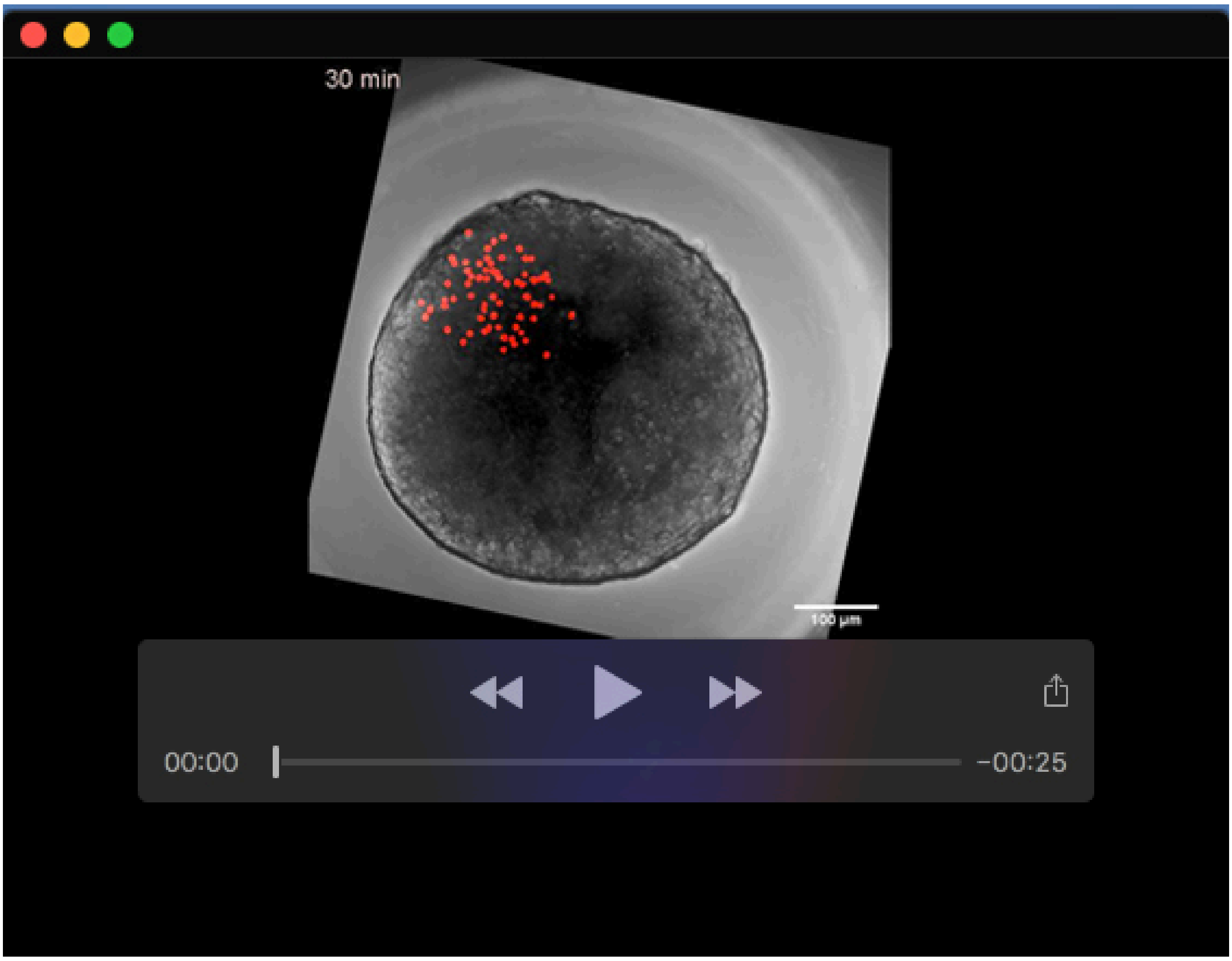

Movie 4. Mesendodermal clone dispersal in wildtype explants. Maximum intensity projection of fluorescence/brightfield high-resolution time-lapse imaging of blastoderm explants showing tracked clonally labeled cell nuclei in the mesendoderm (red) from 24 minutes before the onset of extension (mbe). Spots corresponding to individual tracked mesendodermal progenitors color-coded based on the individual track ID and corresponding cell trajectories generated in Imaris from 24 minutes before the onset of extension (mbe). Time is shown in minutes. Scale bar: 100 µm.

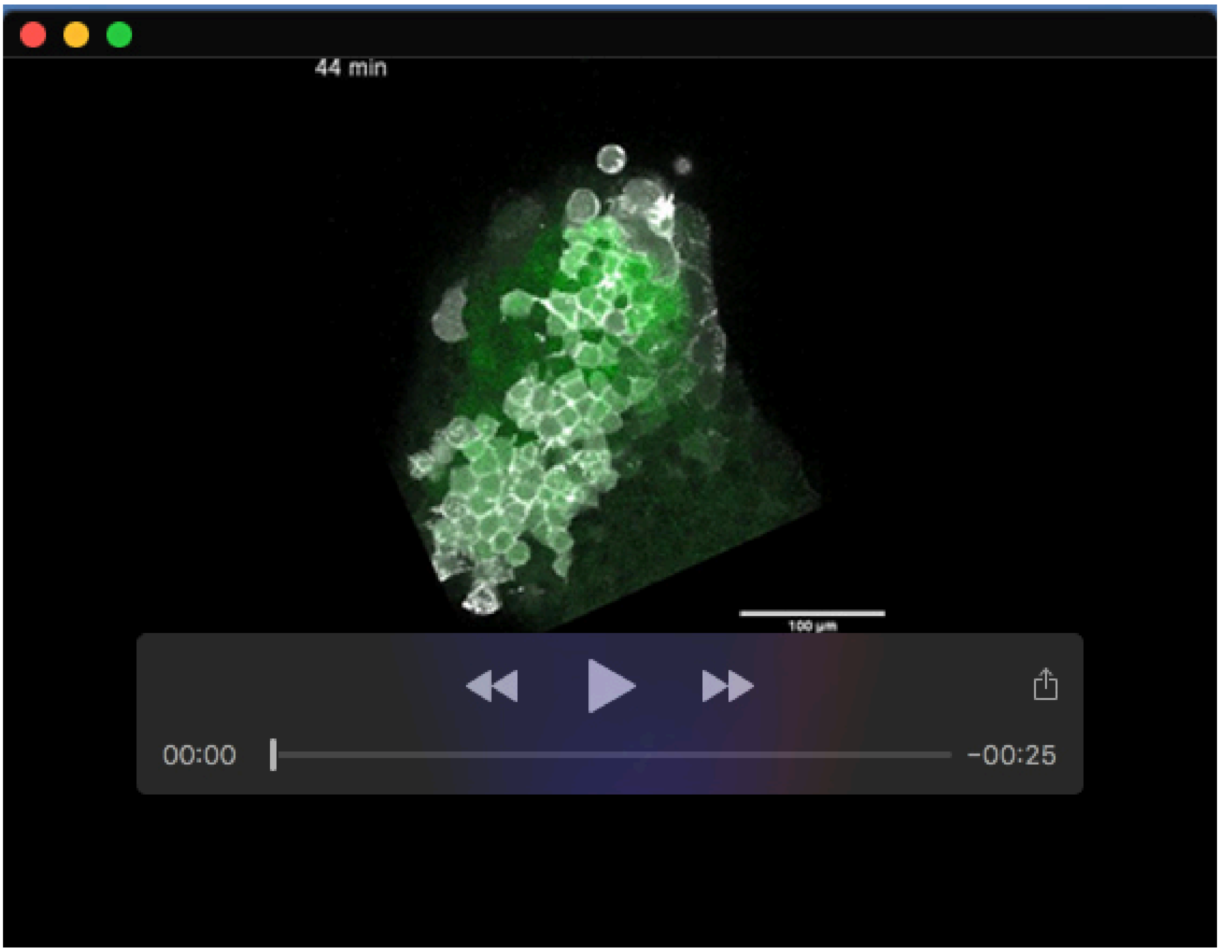

Movie 5. Mesendodermal cell alignment in wildtype explants. Maximum intensity projection of medium planes of high-resolution images of blastoderm explants (side views) obtained from Tg(*sebox::EGFP*) embryos expressing EGFP in mesendoderm progenitors at the onset of explant elongation (corresponding to embryonic shield stage). Mesendodermal cell boundaries within the explant extension are marked by clonal lifeact-RFP (grey) expression. Time is shown in minutes. Scale bar: 100 µm.

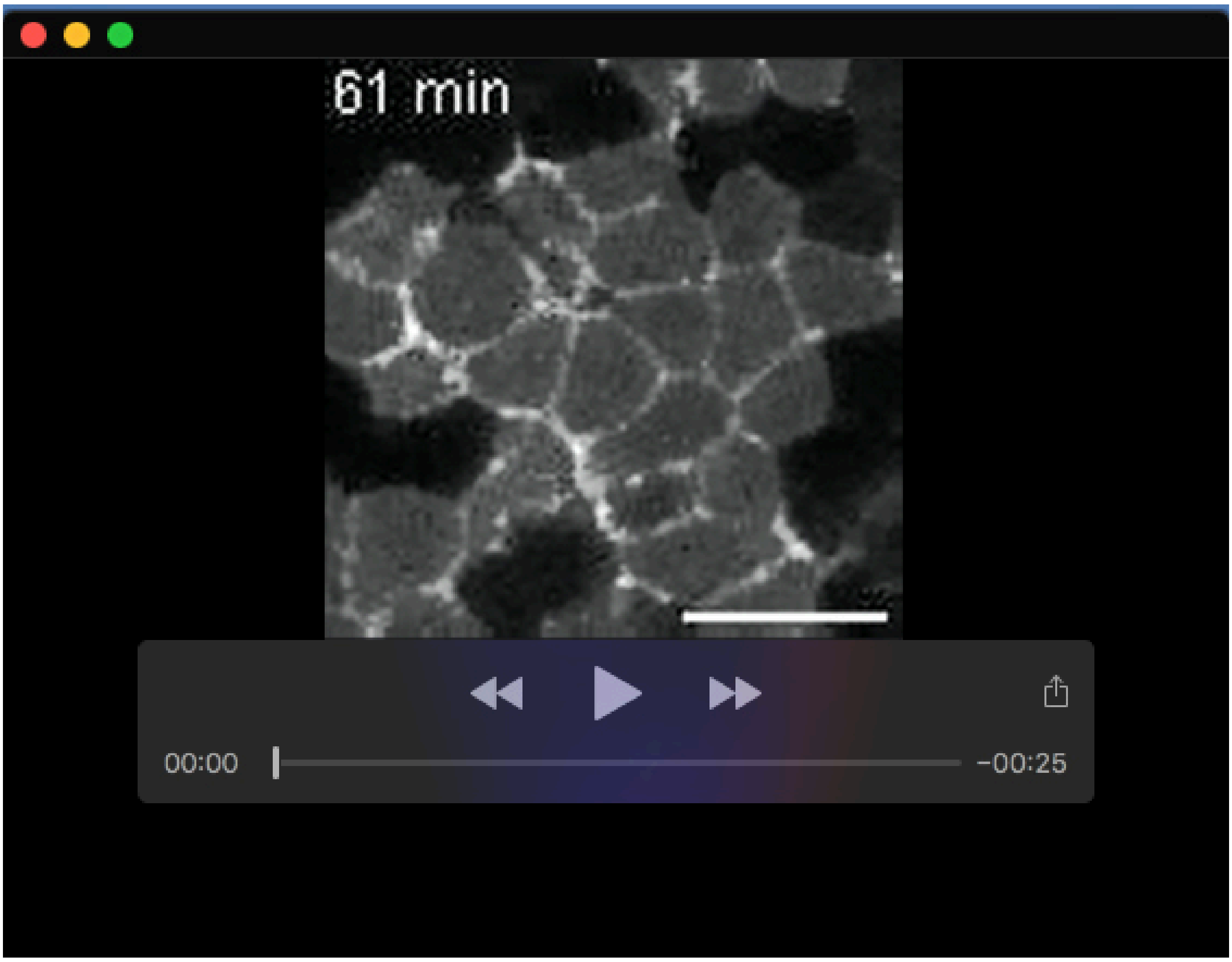

Movie 6. High magnification movie of intercalating mesendodermal progenitors in wildtype explants. Single plane high-resolution images of blastoderm explants (side views) obtained from Tg(*sebox::EGFP*) embryos expressing EGFP in mesendoderm progenitors at the onset of explant elongation (corresponding to embryonic shield stage). Mesendodermal cell boundaries within the explant extension are marked by clonal lifeact-RFP (grey) expression. Time is shown in minutes. Scale bar: 30 µm.

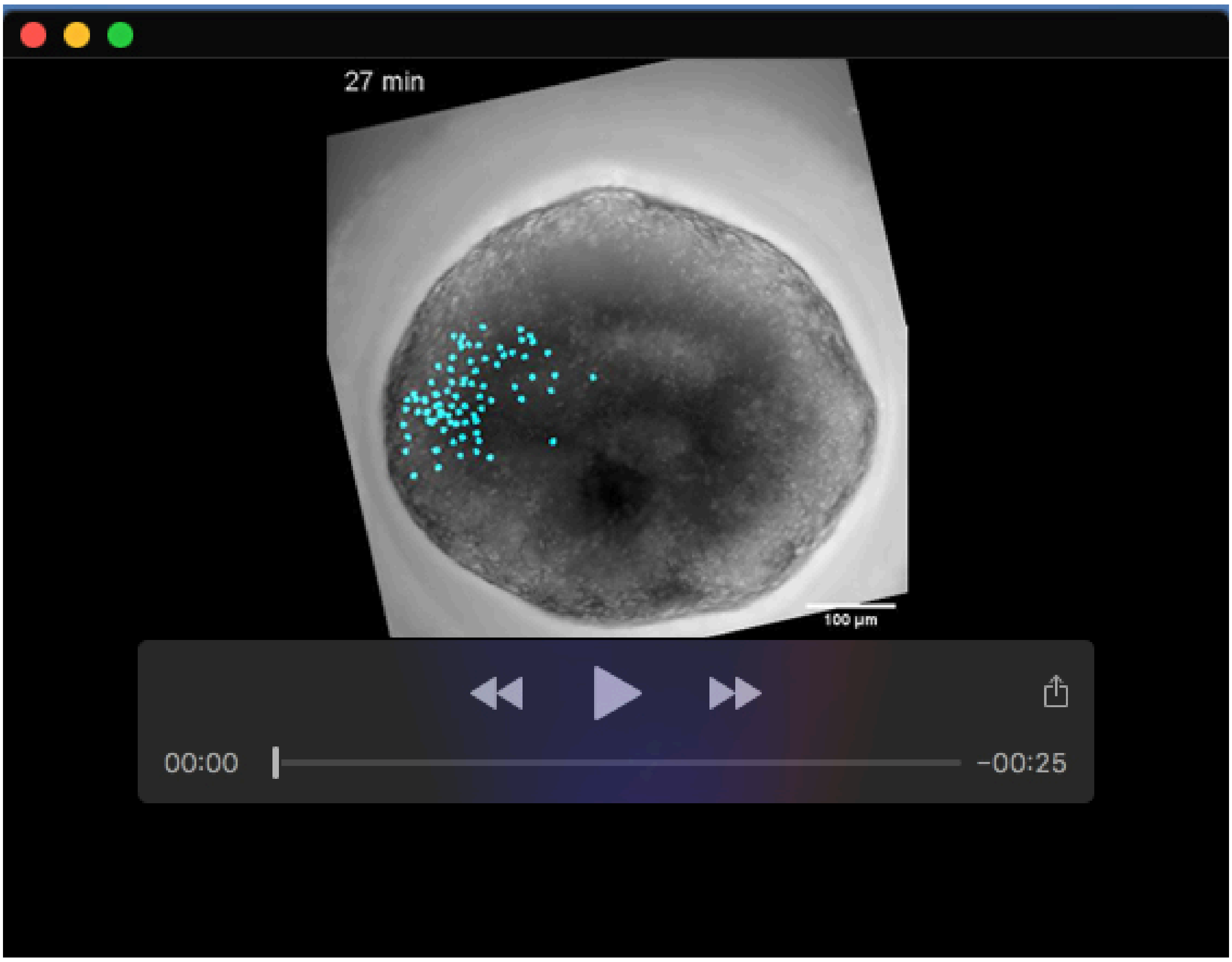

Movie 7. Ectodermal clone dispersal in wildtype explants. Maximum intensity projection of fluorescence/brightfield high-resolution time-lapse imaging of blastoderm explants showing tracked clonally labeled cell nuclei in the ectoderm (blue) from 24 minutes before the onset of extension (mbe). Spots corresponding to individual tracked ectodermal progenitors color-coded based on the individual track ID and corresponding cell trajectories generated in Imaris from 24 minutes before the onset of extension (mbe). Time is shown in minutes. Scale bar: 100 µm.

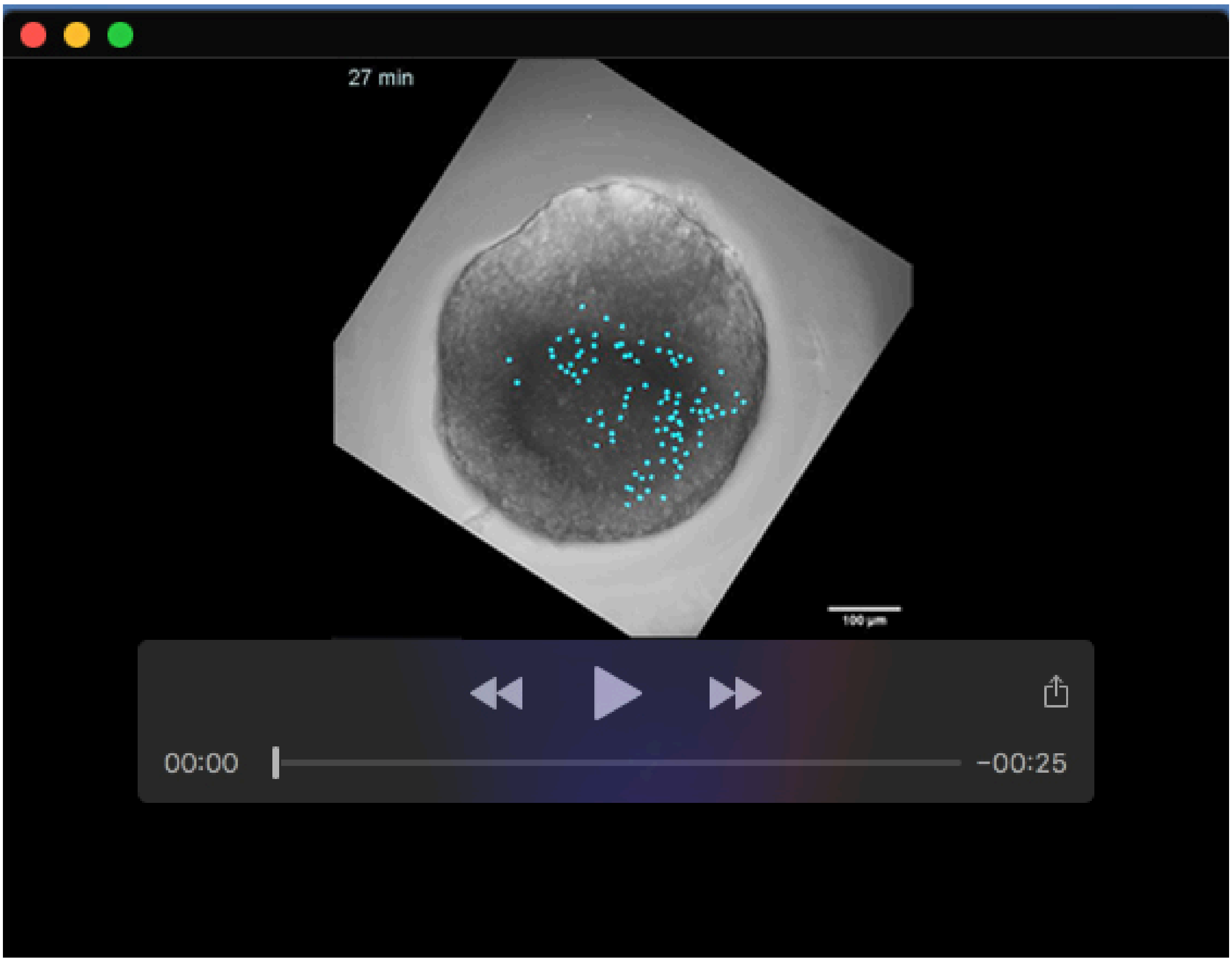

Movie 8. Ectodermal clone dispersal in wildtype explants. Maximum intensity projection of fluorescence/brightfield high-resolution time-lapse imaging of blastoderm explants showing tracked clonally labeled cell nuclei in the ectoderm (blue) from 24 minutes before the onset of extension (mbe). Spots corresponding to individual tracked ectodermal progenitors color-coded based on the individual track ID and corresponding cell trajectories generated in Imaris from 24 minutes before the onset of extension (mbe). Time is shown in minutes. Scale bar: 100 µm.

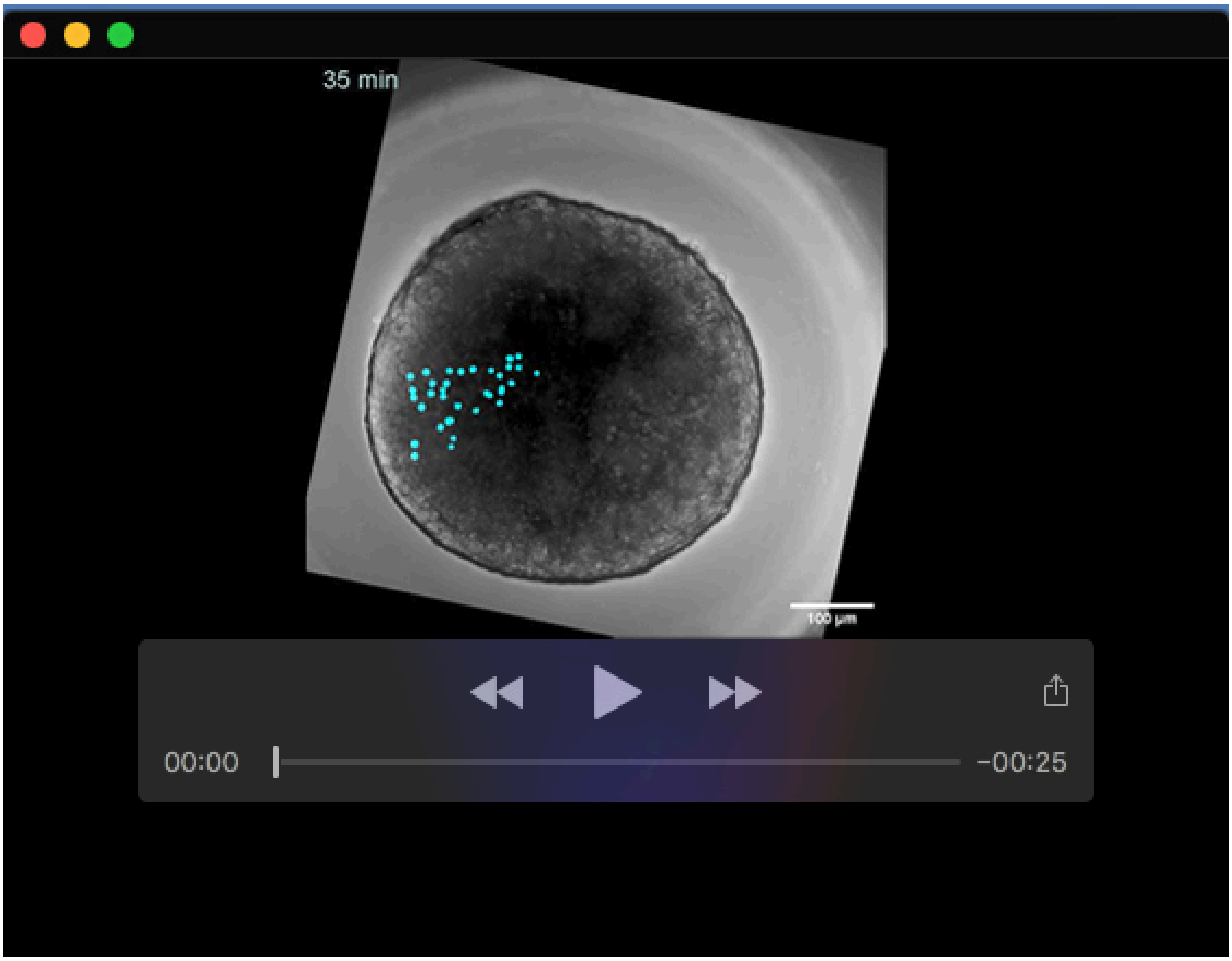

Movie 9. Ectodermal clone dispersal in wildtype explants. Maximum intensity projection of fluorescence/brightfield high-resolution time-lapse imaging of blastoderm explants showing tracked clonally labeled cell nuclei in the ectoderm (blue) from 24 minutes before the onset of extension (mbe). Spots corresponding to individual tracked ectodermal progenitors color-coded based on the individual track ID and corresponding cell trajectories generated in Imaris from 24 minutes before the onset of extension (mbe). Time is shown in minutes. Scale bar: 100 µm.

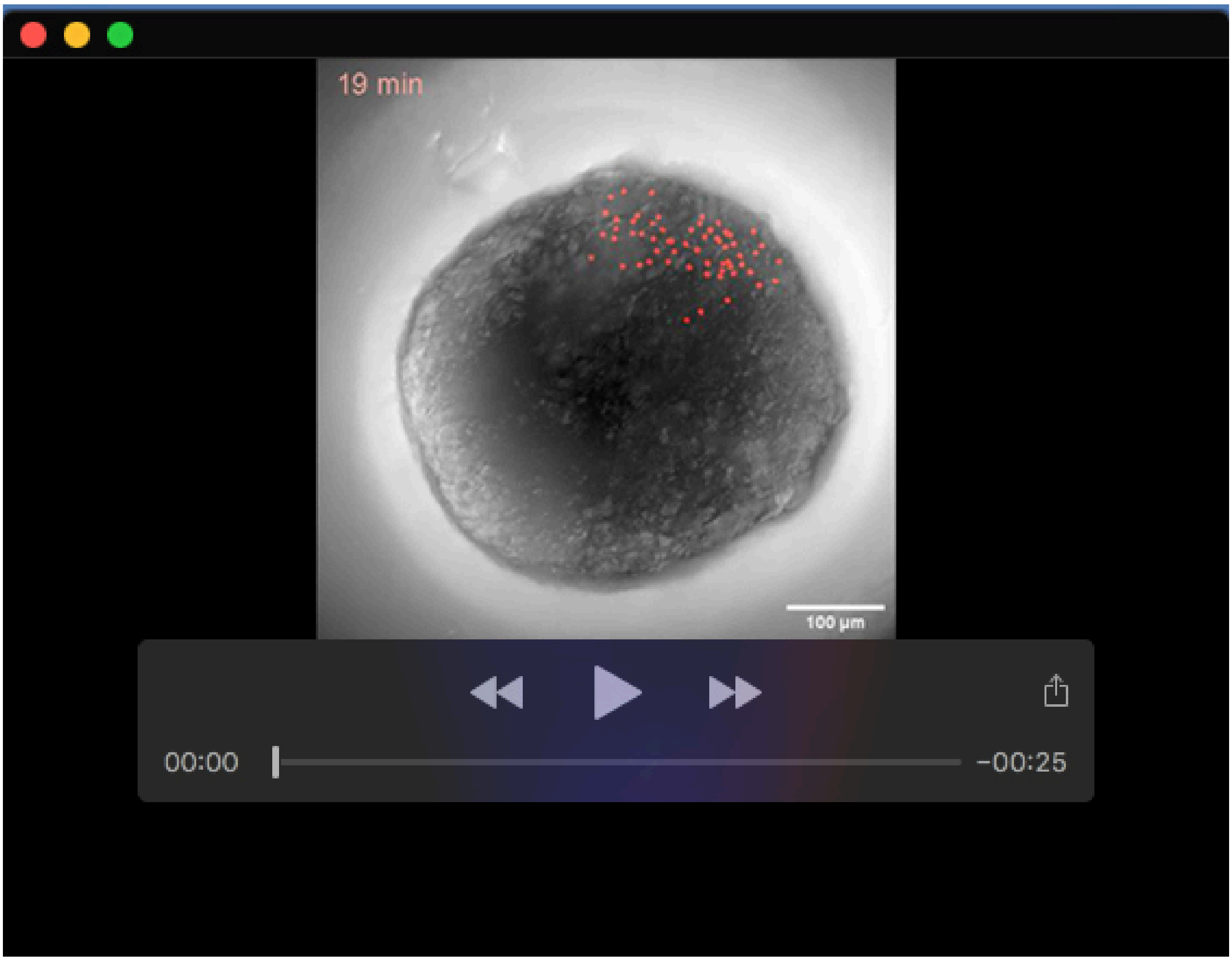

Movie 10. Mesendodermal clone dispersal in *caAlk8* overexpressing explants. Maximum intensity projection of fluorescence/brightfield high-resolution time-lapse imaging of blastoderm explants showing tracked clonally labeled cell nuclei in the mesendoderm (red) of explants prepared from embryos overexpressing 30pg *caAlk8* from 24 minutes before the onset of extension (mbe). Time is shown in minutes. Scale bar: 100 µm.

Development • Supplementary information
