# Supplementary material for: Robust axis elongation by Nodal-dependent restriction of BMP signaling
Source: Development. 2024 Feb 19;151(4):dev202316. doi: 10.1242/dev.202316 (PMC10911127; doi:10.1242/dev.202316)
Supplement: Supplementary file 1 [file develop-151-202316_review_history.pdf]

Robust axis elongation by Nodal-dependent restriction of BMP signaling

Alexandra Schauer, Kornelija Pranjic-Ferscha, Robert Hauschild and Carl-Philipp Heisenberg
DOI: 10.1242/dev.202316

Editor: Steve Wilson

Review timeline

Submission to Review Commons:	25 June 2023
Original submission:	29 August 2023
Editorial decision:	18 September 2023
First revision received:	16 November 2023
Editorial decision:	27 December 2023
Second revision received:	3 January 2024
Accepted:	4 January 2024

Reviewer 1

Evidence, reproducibility and clarity

Summary

The authors presented intriguing observations on the molecular mechanisms regulating morphogenic cell movement, with a particular focus on convergent-extension (CE) movement associated with cell type specification in the zebrafish blastoderm explant. In this manuscript, Schauer et al. identified the CE movement of the mesendoderm as triggering the elongation of the zebrafish embryonic explant. In this process, the Nodal signal represses the BMP signal, which negatively regulates the movement of the mesendoderm precursors, through the induction of its inhibitor chordin. This suggests that the Nodal signal is the key factor coordinating cell fate specification and morphogenesis in the zebrafish blastoderm explant. Finally, suppression of Nodal signalling increases sensitivity to BMP signalling in the CE movement of intact embryos. This suggests that promotion of mesendoderm cell intercalation via BMP suppression by Nodal may be involved in conferring robustness to morphogenic cell movement in vivo.

Major comments

1. While one of the main conclusions of this manuscript is that "Nodal signaling regulates CE movement of mesendodermal cells by promoting their intercalation through inhibition of BMP signaling". However, this was predicted by changes in individual cell morphology and cell dispersal, and the authors didn't directly examine the behavior of individual cells. It would be better to confirm intercalation during the process of explant elongation by cell tracking analysis.
2. Although the authors discuss that Nodal signaling inhibits BMP signaling in the later gastrulation stage, this has not been experimentally tested. If possible, the time window in which Nodal signaling acts should be investigated by temporal inhibition of Nodal signaling using chemical inhibitors.
3. Only the signal gradient of pSmad5 and axis elongation were examined in the intact embryo part of the study (Fig. 6 and Fig. S7). The information on the domain of pSmad2 and the expression of chordin would be helpful for the comparison of the blastoderm explant and the intact embryos.

Minor concerns

© 2024. Published by The Company of Biologists under the terms of the Creative Commons Attribution License (https://creativecommons.org/licenses/by/4.0/).

The first letter of a gene name should be in lowercase. (ex. Fig.S3C; Smad5 MO)

Significance

The zebrafish blastoderm explant assay has the potential to elucidate the molecular mechanisms regulating the complex processes of morphogenesis during vertebrate gastrulation, as the authors demonstrate in this paper. In this manuscript, the authors addressed the molecular mechanism coordinating cell fate specification and morphogenic cell movement in the blastoderm explant. All of the experiments are well-designed, the interpretation of the results is convincing and the paper is well-written. Also, the conclusion is very clear and well supported by the presented data. These findings provide fundamental and important insights for studying morphogenic cell movements in early vertebrate embryos using zebrafish blastoderm explants. On the other hand, most of the molecular mechanisms reported in this manuscript are already predicted by previous studies using intact embryos. Therefore, the impact of this work may be limited to ex vivo research.

Reviewer 2

Evidence, reproducibility and clarity

This paper from the Heisenberg lab takes a reductionist approach to understanding how BMP and Nodal signaling interact to coordinate morphogenesis. They mostly use blastoderm explants that they culture in vitro. These explants elongate over time, with Nodal signaling that induces mesendoderm driving the cell intercalations that explain the elongation. They show that increased BMP signaling inhibits this process, but reducing BMP signaling has no effect. They see that reducing Nodal signaling results in an upregulation of BMP activity as read out by phosphorylated Smad5 staining and increasing Nodal signaling has the opposite effect. They explain this mostly by the observation that Nodal induces the expression of the BMP antagonist, Chordin, and validate this idea by demonstrating that a reduction in Chordin expression reduces explant elongation. Returnign to the embryo, the authors show that manipulation of Nodal signaling levels influences the size of the BMP activity gradient as expected from the in vitro results. Finally, they show that reduction of Nodal signaling with SB505124 sensitises the embryos the effects of bmp2b overexpression, and that BMP overeactivation at 90% epiboly reduced C&E movements.

Major comments

In general I think the work is well done and the data justify the conclusions.

I have several suggestions for additional experiments and discussion that I think would improve the paper.

1. In Figure S1 they present data on elongation of explants treated with a Nodal inhibitor. It would be good to show some examples of images of the explants.
2. In Figure 1G and 3A, the same wildtype images are shown. This is mentioned and I assume therefore that the results were all part of the same experiment. How many times were these experiments performed? It would be much better to use different biological replicates in the two figures.
3. It is important for the authors to make clear how many biological replicates each of the experiments correspond to.
4. In Figure 4E, it would be good to show the levels of P-Smad2 in the Oep and MZ lefty1, 2 explants.
5. On page 11 the authors mention chordin-independent inhibition of BMP signaling. The most likely candidate would be noggin as it too is expressed dorsally and is at least in part activated by Nodal. This should be tested in their model.
6. The authors focus on Chordin as downstream of Nodal signaling, and discuss the role of Nodal signaling in inducing chordin as being due to peak Nodal signaling. However, Chordin has been shown to also be downstream of Fgf signaling and Bozozok (PMIDs 23499658 and 16873584), which likely explains its dorsal expression domain. Furthermore, Rogers et al, (PMID 33174840) who the

© 2024. Published by The Company of Biologists under the terms of the Creative Commons Attribution License (https://creativecommons.org/licenses/by/4.0/).

authors refer to, also show that to disrupt BMP signaling in embryos, inhibition of Nodal and Fgf is required. These issues need to be discussed in more detail. It is the combinatorial signaling that is thought to be responsible for the dorsal location of the chordin (and noggin) expression domains.

Minor comments

I think in general the manuscript is well written and the figures are clear. Previous data is generally well cited. My only comment is that there is a wealth of data from Xenopus and zebrafish that BMP antagonists are induced as a result of combinatorial Nodal signaling and other pathways (dorsal wnt and fgf) that inhibit BMP signaling. I think this could be better referenced.

Significance

The paper is well done and provides important information about the interactions between Nodal and BMP signaling to induce axis elongation. I think the work would be improved if the authors revise it along the lines suggested above. In terms of novelty, many of the component parts of the paper are known (Nodal signaling is important for elongation via cell intercalation and Nodal and BMP can antagonize on another by the induction of BMP antagonists by Nodal), but it is novel to put them together to investigate axis extension using explants. The paper will be of interest to those interested in how these signaling pathways operate in early vertebrate development and to those interested in morphogenesis.

Reviewer 3

Evidence, reproducibility and clarity

Summary:

The manuscript by Schauer et al. uses embryonic explants to study the coordination of Nodal and BMP signaling for embryo morphogenesis. They show that Nodal signaling triggers explant elongation by inducing mesendodermal progenitors that undergo cell intercalation. Looking at the role of BMP signaling, they show that BMP overactivation ventralizes the explants, reducing cell intercalation and therefore explant elongation. Looking at pSmad5, they then establish that BMP signaling in the explant is attenuated by Nodal signaling, through activation of chordin expression, and through some unidentified chordin- independent mechanisms. Moving to the entire embryo, using combinations of BMP overexpression and Nodal inhibition, authors show that Nodal signaling limits BMP signaling on the dorsal side of the embryo, which is key to proper embryo elongation.

Major comments:

- The authors used the sebox::EGFP line to show that the growing region of the explant consists mostly of mesendodermal cells. Although this transgenic line had not been used to do so, the authors and others, had previously demonstrated that the extending part of the explant is mostly made of mesoderm and even shows some patterning (1,2). This should be stated and not presented as a new finding.
- Explant elongation is driven by cell intercalation. The authors analyzed the shape of the mesendodermal tissue to conclude that cells intercalate. While I do not question this conclusion, as it is well known in the embryo, direct observation of cell intercalation, as was done in the embryo (3), would be a better demonstration.
- Explant elongation is driven by mesendodermal cell intercalation. I certainly agree from the movies and images that the extending region is mostly made of mesendoderm. However, it seemed clear to me that in Movie 1, starting at about 140 minutes, most of the convergence movement is taking place in a non-green region of the explant, fueling the extension of the mesendodermal region. Also, to demonstrate that cell intercalation is occurring in the mesendoderm, the authors performed clone dispersal analysis, comparing clones of mesendodermal and ectodermal cells. However, the selected ectodermal clone is very far from the extending region. To show that the cell intercalation is specific to mesendoderm, I think the authors should try to compare the behavior of mesendodermal and non- mesendodermal cells that are located at the same distance from the extending region. For example, from the image in Figure 1E (235 mpe), it appears that

© 2024. Published by The Company of Biologists under the terms of the Creative Commons Attribution License (https://creativecommons.org/licenses/by/4.0/).

the right side of the base of the extended region is not green and could be compared to the left side. Currently, the quantification shown in 1G mostly demonstrates that the extending region is extending, and that the non-extending region is not.
- Based on their observations in explants, the authors propose that Nodal signaling maintains an area of low BMP signaling on the dorsal side of the gastrula for robust axis elongation. While I acknowledge that the experiments performed by the authors have not been previoulsy reported, I did not understand how this differs from the very well established fact that Nodal inhibits BMP signaling, in particular through chordin expression. Von der Hardt for instance already reported that overexpression of bmp and inhibition of chordin leads to severe elongation defects (4). More insight could probably be gained by analyzing the effect in more detail: Is the elongation defect due to cell intercalation defects? How are cell fates affected? Is this Nodal effect mediated by Chordin?...

Minor comments:

- Fig6B. Are the curves significantly different? If so, how were they compared?
- Fig6D-E, I found the quantification a bit confusing. The reader is left with the impression of an all-or-nothing answer (effect only with BMP overexpression and strong Nodal inhibition), whereas the effect on the pSmad5 gradient is gradual. Plotting and comparing the pSmad5 intensity gradients would be better.
- Fig6G. 'Axis length/embryo height' should appear on the x-axis, not the y-axis.

Referees cross-commenting

I feel that the three reviews are very much in agreement, recognising that the experiments carried out are well done and calling for a reasonable amount of additional data. The three reviews also agree that the results obtained here in explants were already known from intact embryos, limiting the relevance to ex vivo research.

Significance

Overall, the experiments appear carefully carried out, and very precisely quantified. The paper is well written and easy to read. The results add to our understanding of the morphogenetic events occurring in embryonic explants. I therefore support their publication.

My main concern is with the significance of the results. I am convinced that embryonic explants are great tools, to reduce the complexity of the embryo and to address questions that cannot be addressed in the embryo, as the authors and others have done, for instance, to address the role of extraembryonic tissues and patterning by maternal contributions. Here, however, I felt that most, if not all, of the experiments essentially demonstrate in embryonic explants, results that have been known for years in the intact embryo. While gathering detailed information on what happens in embryonic explants will certainly prove useful in further understanding the self-organizing abilities of these explants, and is worth publishing, the significance of the results reported here seems limited. Specifically, that elongation is driven by cell intercalation, that BMP-mediated dorsoventral patterning affects cell intercalation, that BMP signaling is attenuated by Nodal through Chordin, that Chordin is required for elongation, has been well established in the embryo over the last 20 years.
Again, showing that it works the same way in embryonic explants is of interest, but at this point, does not add to our understanding of embryonic development.

Author response to reviewers' comments

1. **General Statements**

We thank the reviewers for their positive and constructive feedback. We will incorporate their suggestions as outlined in detail in point 2 and 3 of this document. There is, however, one point, raised in particular by reviewer 3, which questions the novelty of our findings and to which we

© 2024. Published by The Company of Biologists under the terms of the Creative Commons Attribution License (https://creativecommons.org/licenses/by/4.0/).

would like to respond before going into the details of our revision plan. While we acknowledge that some of the regulatory links reported in our study could be inferred from previous studies in the intact embryo, which we have tried to cite extensively throughout the manuscript, there are in particular two main findings in our study - (i) regarding the connection between signaling activity and morphogenesis, and (ii) the processes by which the embryonic system can robustly adapt morphogenetic processes to different boundary conditions - which we believe are novel and important and, to the best of our knowledge, have not yet been shown in vivo or in explants.

1. *Both Nodal and BMP signaling have previously been implicated in the regulation of cell behavior during vertebrate gastrulation (reviewed in Zinski et al., 2018). Yet, how their combined morphogenetic activity domains are spatiotemporally correlated and integrated is only poorly understood. Our study shows that the Nodal-dependent capacity of cells to become polarized and align with the embryonic axis to undergo mediolateral cell intercalations is disrupted by ectopic BMP signaling. This provides insight not only into how the balance of Nodal and BMP signaling regulates the coordination of cell intercalations (mediated by Nodal signaling) and cell migration (mediated by BMP signaling), but also more generally, how such a developmental program is adapted to the specific morphology of the system, i.e. the requirement of convergent migration in the embryo with a large yolk cell, but not in explants lacking such a structure. We will additionally challenge these conclusions in the revision process by further analyzing the relationship between Nodal signaling, changes in BMP signaling levels and cell alignment (see also our detailed response below).*

2. *As one of the hallmarks of vertebrate gastrulation, axis elongation has to be reliable and robust and thus, as outlined above, requires the range of BMP signaling being excluded from the dorsal mesendoderm. Our findings show that Nodal signaling leads to comparatively mild changes of the BMP signaling domain in the intact embryo during gastrulation, while being the key determinant of the pSmad5 domain extent in the explant context. This suggests that apparently redundant factors are important to render the process of BMP gradient formation robust and reproducible under different constraints (Wolpert, 1992). As germ layer patterning is largely determined downstream of maternal dorsal determinants in zebrafish explants (Schauer et al., 2020; Fulton et al., 2020), while a combination of maternal dorsal determinants and extraembryonic signals provided by the yolk syncytial layer are patterning the intact zebrafish embryo (reviewed in Marlow, 2020; Fuentes et al., 2020; Hill, 2022; Concha & Reig, 2022), our findings suggest that in the intact embryonic context this interplay of maternal determinants and extraembryonic signaling factors provides such additional robustness to BMP gradient formation in absence of Nodal signaling. Vice versa, when extraembryonic signaling factors are removed from the system, as in the explants, the BMP signaling gradient becomes more sensitive to variations in Nodal signaling. We will further challenge these conclusions by comparing the sensitivity of chordin expression towards changes in Nodal signaling levels both in vitro and in vivo as well as comparing the changes in the BMP signaling gradient upon gradual reduction of Nodal signaling in both explants and embryos.*

Collectively, we realize that we have not clearly enough explained the novel aspects of our findings in the original version of our manuscript, something we will improve by a combination of additional experiments and revisions of the text.

2. Description of the planned revisions

Please find below our plan of how we will address the reviewer comments. For better overview, we have highlighted suggestions, which have already been incorporated into the updated manuscript in blue and points which we are planning to address in a full revision in red. Similarly, already implemented changes within the updated manuscript are highlighted in blue.

Reviewer #1:

Major comments

1. Although the authors discuss that Nodal signaling inhibits BMP signaling in the later gastrulation stage, this has not been experimentally tested. If possible, the time window in which Nodal signaling acts should be investigated by temporal inhibition of Nodal signaling using chemical inhibitors.

We thank the reviewer for raising this point. To analyze the time window during which Nodal

© 2024. Published by The Company of Biologists under the terms of the Creative Commons Attribution License (https://creativecommons.org/licenses/by/4.0/).

signaling is required for restricting BMP signaling, we have now performed treatments with the Nodal inhibitor SB-505124 in discrete time windows from fertilization until the onset of gastrulation and from the onset of gastrulation until 75% epiboly and analyzed the effect on the pSmad5 domain. These results, shown in Fig.S5K,L and mentioned in the text on page 9/line 259-267, suggest that Nodal signaling is required predominantly prior to the onset of gastrulation to create a domain of low BMP signaling.

We are currently expanding this analysis to also analyze the temporal requirement of Nodal signaling for modulating the pSmad5 gradient in the intact embryo by performing analogous treatments of intact embryos with the Nodal inhibitor SB-505124.

2. Only the signal gradient of pSmad5 and axis elongation were examined in the intact embryo part of the study (Fig. 6 and Fig. S7). The information on the domain of pSmad2 and the expression of chordin would be helpful for the comparison of the blastoderm explant and the intact embryos.

To provide a more comprehensive view of how Nodal signaling and BMP signaling are also coordinated in the intact embryo, we have now included a qPCR analysis of *chordin* expression in MZ*oep* and MZ*lefty1,2* mutant embryos at 75% epiboly, showing that *chordin* expression is strongly downregulated in MZ*oep* mutant embryos and upregulated in MZ*lefty1,2* mutant embryos at 75% epiboly (Fig.S7A, line 332-335, page 11/12), thus corroborating the findings in the explants.

We are currently expanding this analysis by performing double immunostainings for pSmad2 and pSmad5 in MZ*oep* and MZ*lefty1,2* mutant embryos to reveal relative changes in Nodal and BMP signaling domains.

Reviewer #2:

Major comments

In general I think the work is well done and the data justify the conclusions.

I have several suggestions for additional experiments and discussion that I think would improve the paper.

5. On page 11 the authors mention chordin-independent inhibition of BMP signaling. The most likely candidate would be noggin as it too is expressed dorsally and is at least in part activated by Nodal. This should be tested in their model.

We thank the reviewer for raising this very interesting point. Indeed, our preliminary results show increased *noggin* expression in MZ*lefty1,2* mutant explants (Reviewer Figure 1).

We are currently expanding this analysis by functionally analyzing whether this effect on *noggin* expression can account for the Chordin-independent component in the restriction of the pSmad5 domain by Nodal. To this end we are performing the following experiments:

1. Analysis of changes in the pSmad5 domain upon *noggin1* knockdown
2. Analysis of changes in the pSmad5 domain upon *noggin1* knockdown in

- explants derived from embryos with normal Nodal signaling
- explants derived from *chrd*$^{-/-}$ homozygous mutant embryos with normal Nodal signaling
- explants derived from embryos with increased Nodal signaling (MZ*lefty1,2*)
- explants derived from *chrd*$^{-/-}$ homozygous mutant embryos with increased Nodal signaling (MZ*lefty1,2*)

These experiments will complement the analysis in Fig.5I-L. Pending whether Noggin indeed fulfills this role as a Chordin-independent regulator of the pSmad5 domain, we will include these additional findings on the Chordin-independent Nodal-mediated BMP signaling inhibition in the revised version of our manuscript.

© 2024. Published by The Company of Biologists under the terms of the Creative Commons Attribution License (https://creativecommons.org/licenses/by/4.0/).

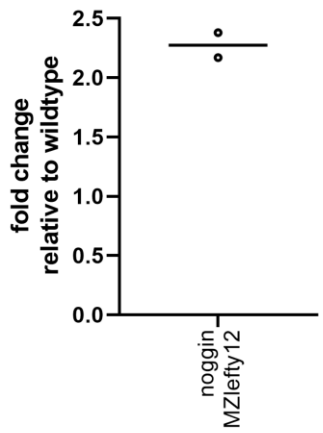

Reviewer figure 1. Upregulation of *noggin* expression in MZ*lefty1,2* mutant explants at 75% epiboly compared to wildtype explants.

Reviewer #3:

Major comments:

- Based on their observations in explants, the authors propose that Nodal signaling maintains an area of low BMP signaling on the dorsal side of the gastrula for robust axis elongation. While I acknowledge that the experiments performed by the authors have not been previoulsy reported, I did not understand how this differs from the very well established fact that Nodal inhibits BMP signaling, in particular through chordin expression. Von der Hardt for instance already reported that overexpression of bmp and inhibition of chordin leads to severe elongation defects (4). More insight could probably be gained by analyzing the effect in more detail: Is the elongation defect due to cell intercalation defects? How are cell fates affected? Is this Nodal effect mediated by Chordin?...

We respectfully disagree with the notion that our experimental set up is equivalent to experiments reported by von der Hardt et al., given that the findings from the von der Hardt et al. study provide evidence for increased BMP signaling leading to axis elongation defects, while our work shows the importance for Nodal signaling in restricting BMP signaling activity to allow proper axis elongation. We also disagree with the notion that Nodal inhibiting BMP signaling is already a well- established fact. Reports to date have been limited to analyzing the effect of Nodal signaling on the expression of different components of the BMP signaling network, amongst those negative regulators, such as *chordin* (Gritsman et al., 1999; Sirotkin et al., 2000; Bennett et al., 2007; Varga et al., 2007), but also positive regulators, such as *bmp4* and *admp* (Gritsman et al., 1999; Lele et al., 2001), thus precluding easy predictions of how the actual domain of BMP signaling activity is affected by changes in Nodal signaling levels. In fact, the only study as of yet, which actually addressed the effect of Nodal signaling on BMP signaling activity, has shown that BMP signaling is largely independent of Nodal signaling until the onset of gastrulation (Rogers et al., 2020). Our work also goes beyond just showing that BMP signaling activity is affected by changes in Nodal signaling levels, by demonstrating how high levels of Nodal signaling allow the embryo to buffer small increases of *bmp*, thereby providing robustness to the axis elongation process. We thus believe that our findings provide important conceptual advances over previous studies on this topic.

This said, we agree with the reviewer's assessment that important questions remain in the von der Hardt study as to the cellular basis of the observed axis elongation defects in BMP overexpressing embryos. In our present study, we have addressed this question by using explants as a simplified model of axis elongation that predominantly shows hallmarks of cell intercalation movements in the mesendoderm, rather than a combination of cell intercalation and convergent migration as found in the intact embryo. We show that cell polarization and alignment, prerequisites for medially-oriented intercalations (Glickman et al., 2003; Williams & Solnica-Krezel, 2020), are disrupted by overactivation of BMP signaling (Fig.3, Fig.S4), a phenotype that is also detectable in the dorsal mesendoderm of intact embryos (Fig.S7H,I).

To further challenge these results, we will perform the following analysis:

1. Analysis of the relationship between pSmad5 levels and cell polarization and alignment in wildtype and 1 pg *bmp2b* overexpressing explants at 90% epiboly (preliminary results in Reviewer figure 2)

© 2024. Published by The Company of Biologists under the terms of the Creative Commons Attribution License (https://creativecommons.org/licenses/by/4.0/).

2. Analysis of the relationship between pSmad5 levels and dorsal cell polarization and alignment in intact embryos at 90% epiboly in
   - wildtype embryos
   - embryos with reduced Nodal signaling
   - 1 pg *bmp2b* overexpressing embryos
   - 1 pg *bmp2b* overexpressing embryos with reduced Nodal signaling
   - 5 pg *bmp2b* overexpressing embryos
   - 5 pg *bmp2b* overexpressing embryos with reduced Nodal signaling

   This will hopefully provide further novel insights into the relationship between BMP signaling levels and cell polarization as well as relate these phenomena with the extent of overall axis elongation as shown in Fig.6F,G.

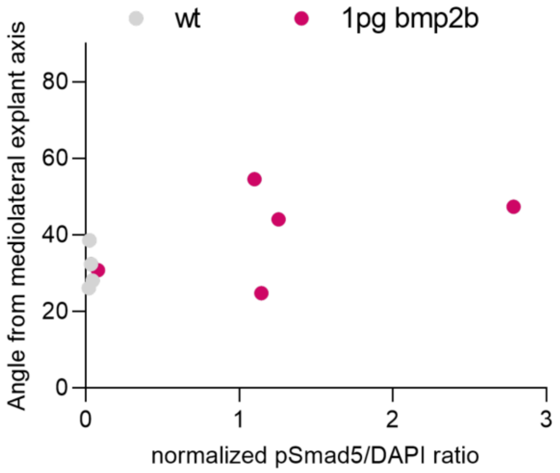

Reviewer figure 2. Cell alignment as assessed by the angle of the longest axis of mesendodermal progenitors from the main explant axis as a function of pSmad5 levels in wildtype and 1 pg *bmp2b* overexpressing explants at 90% epiboly.

Minor comments:

- Fig6B. Are the curves significantly different? If so, how were they compared?

We will include a table of p values for each bin in comparison to wildtype.

- Fig6D-E, I found the quantification a bit confusing. The reader is left with the impression of an all-or-nothing answer (effect only with BMP overexpression and strong Nodal inhibition), whereas the effect on the pSmad5 gradient is gradual. Plotting and comparing the pSmad5 intensity gradients would be better.

We will add quantifications of the gradients as in Fig.6B.

3. Description of the revisions that have already been incorporated in the transferred manuscript

Reviewer #1:

Major comments

1. While one of the main conclusions of this manuscript is that "Nodal signaling regulates CE movement of mesendodermal cells by promoting their intercalation through inhibition of BMP signaling". However, this was predicted by changes in individual cell morphology and cell dispersal, and the authors didn't directly examine the behavior of individual cells. It would be better to confirm intercalation during the process of explant elongation by cell tracking analysis.

The analysis of cell dispersal was actually performed based on the coordinates of individually tracked cells by calculating the distance of each cell to all other cells in the mediolateral and anterior-posterior direction of the main explant axis at each time point as this would allow us to capture the key features of cell intercalation behavior. We have changed the text to point this out more clearly. The reason why we quantified changing distances between cells in the two directions

© 2024. Published by The Company of Biologists under the terms of the Creative Commons Attribution License (https://creativecommons.org/licenses/by/4.0/).

rather than effective cell displacement is that the samples can move drastically during the live imaging as they cannot be constrained, which greatly affects the values for cell displacements and directional velocity. In contrast, quantifying the relative distances between individual cells is less sensitive towards such artifacts. We have included movies showing the cell tracks and cell movement along the tracks to further illustrate the cell intercalation behavior (Supplementary Videos 5,11). Additionally, we have included a higher magnification movie of the clonally labelled cell outlines to show cell intercalations (Supplementary Video 7).

Minor concerns
The first letter of a gene name should be in lowercase. (ex. Fig.S3C; Smad5 MO)
Thank you for noting! This has been changed throughout the manuscript.

Reviewer #2:
Major comments
1. In Figure S1 they present data on elongation of explants treated with a Nodal inhibitor. It would be good to show some examples of images of the explants.
We have now included images of such explants (Fig. S1G).

2. In Figure 1G and 3A, the same wildtype images are shown. This is mentioned and I assume therefore that the results were all part of the same experiment. How many times were these experiments performed? It would be much better to use different biological replicates in the two figures.
As mentioned in the original version of the manuscript, the data for the clone dispersal in the wildtype explants correspond to the data presented in Fig.1G,H and are derived from 5 individual explants from 5 independent experiments. We have now more explicitly pointed this out in the figure legends. Given the difficulty of acquiring movies of explant elongation, and that we see very consistent behavior in the overall clone dispersal in wildtype (Fig.S1I,I') as well as *caAlk8* overexpressing explants (Fig.S4A), we believe that it is legitimate to show data from the same biological replicates in the two figures. Moreover, we have now also included two more sample movies (Supplementary Video 3,4 for mesendodermal clones, Supplementary Video 9,10 for ectodermal clones) of wildtype explants for the clone dispersal data to provide the reader with a more comprehensive view of the cell rearrangements in several different examples of this experiment. Finally, we will try to acquire and analyze more movies to increase the overall sample size; however, due to the difficulty of acquiring movies in the correct orientation and sample movement during imaging, we are not yet sure whether this is achievable within a reasonable timeframe of revision.

3. It is important for the authors to make clear how many biological replicates each of the experiments correspond to.
We thank the reviewer for noting that this might not be fully clear. We have reported in the figure legends the number of analyzed samples (n – individual explants or embryos respectively) and biological replicates (N – independent biological replicates) for each experiment and the number of individually analyzed cells if applicable. We have now included a more explicit definition of what we report as n and N in the statistics section of the methods (page 53, line 1371-1373). We hope that this is now clearer.

4. In Figure 4E, it would be good to show the levels of P-Smad2 in the Oep and MZ lefty1, 2 explants.
As suggested, we have performed stainings for pSmad2 in MZ*oep* and MZ*lefty1,2* explants to verify that the findings from intact embryos on the changes in pSmad2 levels can be translated to the explant context. The results are now mentioned on page 9/line 248-249 and shown in Fig.S5A,B.

6. The authors focus on Chordin as downstream of Nodal signaling, and discuss the role of Nodal signaling in inducing chordin as being due to peak Nodal signaling. However, Chordin has been shown to also be downstream of Fgf signaling and Bozozok (PMIDs 23499658 and 16873584), which likely explains its dorsal expression domain. Furthermore, Rogers et al, (PMID 33174840) who the authors refer to, also show that to disrupt BMP signaling in embryos, inhibition of Nodal and Fgf is required. These issues need to be discussed in more detail. It is the combinatorial signaling that is thought to be responsible for the dorsal location of the chordin (and noggin) expression domains.
We thank you for raising this point. We have now tried to reference the combinatorial signaling

© 2024. Published by The Company of Biologists under the terms of the Creative Commons Attribution License (https://creativecommons.org/licenses/by/4.0/).

nature which is integral to the regulation of BMP signaling more extensively. This can be found on page 14/15/ line 415, 438-449.

Minor comments
I think in general the manuscript is well written and the figures are clear. Previous data is generally well cited. My only comment is that there is a wealth of data from Xenopus and zebrafish that BMP antagonists are induced as a result of combinatorial Nodal signaling and other pathways (dorsal wnt and fgf) that inhibit BMP signaling. I think this could be better referenced.
As mentioned above, we are discussing these important points more extensively on page 14/15/ line 415, 438-449.

Reviewer #3:
Major comments:
- The authors used the sebox::EGFP line to show that the growing region of the explant consists mostly of mesendodermal cells. Although this transgenic line had not been used to do so, the authors and others, had previously demonstrated that the extending part of the explant is mostly made of mesoderm and even shows some patterning (1,2). This should be stated and not presented as a new finding.
We thank the reviewer for raising this point. We have now changed the text (page 4/ line 102- 107) to refer to the earlier work showing that the explant extension mainly consists of mesendoderm and to clarify that the novelty of the presented result lies in dissecting the contribution of different germ layer progenitors to the formation of the explant extension.

- Explant elongation is driven by cell intercalation. The authors analyzed the shape of the mesendodermal tissue to conclude that cells intercalate. While I do not question this conclusion, as it is well known in the embryo, direct observation of cell intercalation, as was done in the embryo (3), would be a better demonstration.
Our conclusions are not solely based on the analysis of the morphology of the mesendodermal tissue, but also on the following features:

- Elongation and narrowing of mesendodermal cell clones (Fig.1G-I, Fig.S1I,I'). Notably, this analysis of the dispersal pattern is performed on individually tracked progenitors (see response to reviewer 1).
- Polarization and medially-oriented alignment of mesendodermal progenitors perpendicular to the axis of explant elongation (Fig.1J,K, Fig.S1K).

To further demonstrate the cell intercalation behavior of mesendodermal progenitors, we have now included movies of the cell movement with the corresponding cell tracks as well as high magnification movies of the cell outlines which show the intercalation events (Supplementary Video 5, 7, 11).

- Explant elongation is driven by mesendodermal cell intercalation. I certainly agree from the movies and images that the extending region is mostly made of mesendoderm. However, it seemed clear to me that in Movie 1, starting at about 140 minutes, most of the convergence movement is taking place in a non-green region of the explant, fueling the extension of the mesendodermal region. Also, to demonstrate that cell intercalation is occurring in the mesendoderm, the authors performed clone dispersal analysis, comparing clones of mesendodermal and ectodermal cells. However, the selected ectodermal clone is very far from the extending region. To show that the cell intercalation is specific to mesendoderm, I think the authors should try to compare the behavior of mesendodermal and non-mesendodermal cells that are located at the same distance from the extending region. For example, from the image in Figure 1E (235 mpe), it appears that the right side of the base of the extended region is not green and could be compared to the left side. Currently, the quantification shown in 1G mostly demonstrates that the extending region is extending, and that the non-extending region is not.
We thank the reviewer for raising this very important point and would like to clarify that we do not want to claim that cell intercalation behavior is unique to the mesendoderm. As pointed out by the reviewer and in the original version of our manuscript, ectodermal cells also show a dispersal pattern indicative of cell intercalations, in line with previous studies (Williams & Solnica-Krezel, 2020); however, ectodermal cell rearrangements seem to be restricted to later stages of the explant elongation (Fig.1I). In line with the reviewer's suggestion, we have now analyzed the elongation of the ectoderm and mesendoderm by quantifying the change in the relative location of

© 2024. Published by The Company of Biologists under the terms of the Creative Commons Attribution License (https://creativecommons.org/licenses/by/4.0/).

the boundary of the mesendoderm and ectoderm closest to the extension (Fig. S1F), circumventing potential biases due to clone location. This shows - similarly to the clone dispersal
- that the mesendoderm elongates earlier and to a larger extent than the ectoderm within the analyzed timeframe. These results are now mentioned on page 4,5/line117-121. We have also revised the text to more explicitly mention this point and explain that, although the elongation movement seems to be initiated by mesendodermal progenitors (Fig.1C-H, Fig.S1D), the coordinated activity of ectodermal and mesendodermal C&E movements is most likely required to reach the full elongation potential page 6/line148-156 (see also Discussion page 15,16/ line 456-458).

Minor comments:
- Fig6G. 'Axis length/embryo height' should appear on the x-axis, not the y-axis.

This has been changed.

4. Description of analyses that authors prefer not to carry out

Please include a point-by-point response explaining why some of the requested data or additional analyses <u>might not be necessary or cannot be provided within the scope of a revision</u>. This can be due to time or resource limitations or in case of disagreement about the necessity of such additional data given the scope of the study. Please leave empty if not applicable.

Original submission

<u>First decision letter</u>

MS ID#: DEVELOP/2023/202316

MS TITLE: Robust axis elongation by Nodal-dependent restriction of BMP signaling

AUTHORS: Alexandra Schauer, Kornelija Pranjic-Ferscha, Robert Hauschild, and Carl-Philipp Heisenberg

Apologies for the delay in making a decision on your manuscript submitted through Review Commons. I have now read the referee reports and your responses to them including the experiments you have done and the ones you intend to do. I think that your suggestions are all reasonable and if the referees are happy with your revisions, then we will be happy to publish the manuscript in Development. I should add that I accept your comments in rebuttal of reviewer 3 questioning the novelty/impact of the work. You can access this letter and all other files on BenchPressand click on the 'Manuscripts with Decisions' queue in the Author Area.

Please complete your additional experiments and attend to all of the reviewers' comments and ensure that you clearly highlight all changes made in the revised manuscript. Please avoid using 'Tracked changes' in Word files as these are lost in PDF conversion. I should be grateful if you would also provide a point-by-point response detailing how you have dealt with the points raised by the reviewers in the 'Response to Reviewers' box.

First revision

<u>Author response to reviewers' comments</u>

We want to thank all reviewers for their constructive feedback. Please find below our point-by-point response. We have indicated the page numbers and line numbers of key changes as shown in the compiled PDF of the main text and figures.

© 2024. Published by The Company of Biologists under the terms of the Creative Commons Attribution License (https://creativecommons.org/licenses/by/4.0/).

Reviewer #1 (Evidence, reproducibility and clarity (Required)):

Summary
The authors presented intriguing observations on the molecular mechanisms regulating morphogenic cell movement, with a particular focus on convergent-extension (CE) movement associated with cell type specification in the zebrafish blastoderm explant. In this manuscript, Schauer et al. identified the CE movement of the mesendoderm as triggering the elongation of the zebrafish embryonic explant. In this process, the Nodal signal represses the BMP signal, which negatively regulates the movement of the mesendoderm precursors, through the induction of its inhibitor chordin. This suggests that the Nodal signal is the key factor coordinating cell fate specification and morphogenesis in the zebrafish blastoderm explant. Finally, suppression of Nodal signalling increases sensitivity to BMP signalling in the CE movement of intact embryos. This suggests that promotion of mesendoderm cell intercalation via BMP suppression by Nodal may be involved in conferring robustness to morphogenic cell movement in vivo.

Major comments
1. While one of the main conclusions of this manuscript is that "Nodal signaling regulates CE movement of mesendodermal cells by promoting their intercalation through inhibition of BMP signaling". However, this was predicted by changes in individual cell morphology and cell dispersal, and the authors didn't directly examine the behavior of individual cells. It would be better to confirm intercalation during the process of explant elongation by cell tracking analysis.
The analysis of cell dispersal was actually performed based on the coordinates of individually tracked cells by calculating the distance of each cell to all other cells in the mediolateral and anterior-posterior direction of the main explant axis at each time point, allowing us to capture the key features of cell intercalation behavior based on the movement of individual cells. We have revised the text to point this out more clearly.
The reason why we quantified changing distances between cells in the two directions rather than effective cell displacement is that the samples often shift during the live imaging, which greatly affects the values for cell displacements and directional velocity. In contrast, we found quantifying the relative distances between individual cells to be less sensitive towards such artifacts, while still taking individual cell movement over time into account. We have included movies showing the cell tracks and cell movement along the tracks to further illustrate the cell intercalation behavior (Supplementary Videos 2-4, 7-9). Additionally, we have included a higher magnification movie of the clonally labeled cell outlines to directly visualize several examples of cell intercalation events (Supplementary Video 6).

2. Although the authors discuss that Nodal signaling inhibits BMP signaling in the later gastrulation stage, this has not been experimentally tested. If possible, the time window in which Nodal signaling acts should be investigated by temporal inhibition of Nodal signaling using chemical inhibitors.
We thank the reviewer for raising this point. To analyze the time window during which Nodal signaling is required for restricting BMP signaling, we have now performed treatments with the Nodal inhibitor SB-505124 in consecutive time windows from fertilization until the onset of gastrulation and from the onset of gastrulation until 75% epiboly and analyzed the effect on the pSmad5 domain. These results, shown in Fig. S5K,L and mentioned in the text on page 9, line 243-250, suggest that Nodal signaling is required predominantly prior to the onset of gastrulation to create a domain of low BMP signaling in the explants. In line with this, treatments from 4-16-cell stage until shield stage led to an expansion of the pSmad5 gradient in intact embryos at 75% epiboly, while treatment only from shield stage until 75% epiboly did not clearly alter the gradient profile (Fig. S8G-L, page 12/line 339-343).

3. Only the signal gradient of pSmad5 and axis elongation were examined in the intact embryo part of the study (Fig. 6 and Fig. S7). The information on the domain of pSmad2 and the expression of chordin would be helpful for the comparison of the blastoderm explant and the intact embryos.
Thank you for pointing this out! To provide a more comprehensive view of how Nodal signaling and BMP signaling are also coordinated in the intact embryo, we have now included a qPCR analysis of *chordin* expression in MZ*oep* and MZ*lefty1,2* mutant embryos at 75% epiboly, showing that *chordin* expression is strongly downregulated in MZ*oep* mutant embryos and upregulated in MZ*lefty1,2* mutant embryos at 75% epiboly (Fig. S8B, page 11/line 327-329), similar to the findings in the

© 2024. Published by The Company of Biologists under the terms of the Creative Commons Attribution License (https://creativecommons.org/licenses/by/4.0/).

explants. We have further performed double immunostainings for pSmad2 and pSmad5 in embryos at 75% epiboly to reveal the relative Nodal and BMP signaling domains, which showed that nuclear pSmad2 is found in the margin and dorsal axis, while nuclear pSmad5 forms a gradient from ventral to dorsal across the animal-vegetal axis (Fig. S8A, page 11/line 321-326) (reviewed in Zinski et al., 2018, Rogers & Müller, 2019; Hill, 2022).

Minor concerns
The first letter of a gene name should be in lowercase. (ex. Fig.S3C; Smad5 MO)
This has been changed throughout the manuscript.

Reviewer #1 (Significance (Required)):
The zebrafish blastoderm explant assay has the potential to elucidate the molecular mechanisms regulating the complex processes of morphogenesis during vertebrate gastrulation, as the authors demonstrate in this paper. In this manuscript, the authors addressed the molecular mechanism coordinating cell fate specification and morphogenic cell movement in the blastoderm explant. All of the experiments are well- designed, the interpretation of the results is convincing and the paper is well-written.
Also, the conclusion is very clear and well supported by the presented data. These findings provide fundamental and important insights for studying morphogenic cell movements in early vertebrate embryos using zebrafish blastoderm explants. On the other hand, most of the molecular mechanisms reported in this manuscript are already predicted by previous studies using intact embryos. Therefore, the impact of this work may be limited to ex vivo research.

Reviewer #2 (Evidence, reproducibility and clarity (Required)):
This paper from the Heisenberg lab takes a reductionist approach to understanding how BMP and Nodal signaling interact to coordinate morphogenesis. They mostly use blastoderm explants that they culture in vitro. These explants elongate over time, with Nodal signaling that induces mesendoderm driving the cell intercalations that explain the elongation. They show that increased BMP signaling inhibits this process, but reducing BMP signaling has no effect. They see that reducing Nodal signaling results in an upregulation of BMP activity as read out by phosphorylated Smad5 staining and increasing Nodal signaling has the opposite effect. They explain this mostly by the observation that Nodal induces the expression of the BMP antagonist, Chordin, and validate this idea by demonstrating that a reduction in Chordin expression reduces explant elongation. Returnign to the embryo, the authors show that manipulation of Nodal signaling levels influences the size of the BMP activity gradient as expected from the in vitro results. Finally, they show that reduction of Nodal signaling with SB505124 sensitises the embryos the effects of bmp2b overexpression, and that BMP overeactivation at 90% epiboly reduced C&E movements.

Major comments
In general I think the work is well done and the data justify the conclusions.
I have several suggestions for additional experiments and discussion that I think would improve the paper.

1. In Figure S1 they present data on elongation of explants treated with a Nodal inhibitor. It would be good to show some examples of images of the explants.
We have now included images of such explants (Fig. S1G).

2. In Figure 1G and 3A, the same wildtype images are shown. This is mentioned and I assume therefore that the results were all part of the same experiment. How many times were these experiments performed? It would be much better to use different biological replicates in the two figures.
As mentioned in the original version of the manuscript, the data for the clone dispersal in the wildtype explants correspond to the data presented in Fig.1G,H and are derived from 5 individual explants from 5 independent experiments. We have now more explicitly pointed this out in the figure legends. Given the difficulty and low efficiency of acquiring movies of explant elongation, and that we see consistent behavior in the overall clone dispersal between wildtype (Fig.S1I,I') as well as *caAlk8* overexpressing explants (Fig.S4A), we believe that it is legitimate to show data from the same biological replicates in the two figures. Still, we fully agree with the reviewer that it would be beneficial to provide the readers with more examples of how the elongation process in

© 2024. Published by The Company of Biologists under the terms of the Creative Commons Attribution License (https://creativecommons.org/licenses/by/4.0/).

the explants looks like and have thus included two additional sample movies (Supplementary Video S2-4 for mesendodermal clones, Supplementary Video S7-9 for ectodermal clones) of wildtype explants corresponding to the clone dispersal data.

3. It is important for the authors to make clear how many biological replicates each of the experiments correspond to.
We had reported in the figure legends the number of analyzed samples (n – individual explants or embryos respectively) and biological replicates (N – independent biological replicates) for each experiment and the number of individually analyzed cells if applicable. We have now also included a more explicit definition of what we report as n and N in the statistics section of the methods (page 39/line 1079-1080).

4. In Figure 4E, it would be good to show the levels of P-Smad2 in the Oep and MZ lefty1, 2 explants.
As suggested, we have performed stainings for pSmad2 in MZ*oep* and MZ*lefty1,2* explants to verify that the findings from intact embryos on the changes in the pSmad2 domain extent can be translated to the explant context. The results are now mentioned on page 8/line 232-233 and shown in Fig.S5A,B.

5. On page 11 the authors mention chordin-independent inhibition of BMP signaling. The most likely candidate would be noggin as it too is expressed dorsally and is at least in part activated by Nodal. This should be tested in their model.
We thank the reviewer for raising this very interesting point. We have now examined the role of *noggin1* for restricting BMP signaling activity downstream of Nodal signaling.
These results are presented in a new supplementary figure (Figure S7) and discussed on page 10-11/line 298-315. In short, we find that *noggin1* expression is strongly upregulated in MZ*lefty1,2* mutant explants relative to wildtype explants (Fig. S7A), while knockdown of *noggin1* on its own does not significantly affect explant elongation or the BMP signaling domain (Fig. S7B-G). However, knockdown of *noggin1* in absence of *chrd* can cause the pSmad5 domain to expand across the full explant axis, even in MZ*lefty1,2* explants (Fig. S7H-J). This suggests that *noggin1* functions downstream of Nodal signaling and redundantly with *chrd* in restricting the BMP signaling domain. We have mentioned these results on page 10-11/line 298-315.

6. The authors focus on Chordin as downstream of Nodal signaling, and discuss the role of Nodal signaling in inducing chordin as being due to peak Nodal signaling. However, Chordin has been shown to also be downstream of Fgf signaling and Bozozok (PMIDs 23499658 and 16873584), which likely explains its dorsal expression domain. Furthermore, Rogers et al, (PMID 33174840) who the authors refer to, also show that to disrupt BMP signaling in embryos, inhibition of Nodal and Fgf is required. These issues need to be discussed in more detail. It is the combinatorial signaling that is thought to be responsible for the dorsal location of the chordin (and noggin) expression domains.
We have now more extensively referenced the combinatorial signaling nature which is integral to the regulation of BMP signaling, with particular emphasis on the potential implications for the observed differences in the penetrance of the Nodal phenotype between explants and embryos. This can be found on page 15/line 431-434 and page 15-16/line 452-466.

Minor comments
I think in general the manuscript is well written and the figures are clear. Previous data is generally well cited. My only comment is that there is a wealth of data from Xenopus and zebrafish that BMP antagonists are induced as a result of combinatorial Nodal signaling and other pathways (dorsal wnt and fgf) that inhibit BMP signaling. I think this could be better referenced.
As mentioned above, we are discussing these important points more extensively on page 15/line 431-433 and page 15-16/line 452-466. We hope this now provides a more detailed view of the complex combinatorial nature of the regulation of BMP signaling effectors.

Reviewer #2 (Significance (Required)):
The paper is well done and provides important information about the interactions between Nodal and BMP signaling to induce axis elongation. I think the work would be improved if the authors revise it along the lines suggested above. In terms of novelty, many of the component parts of the paper are known (Nodal signaling is important for elongation via cell intercalation and Nodal and BMP can antagonize on another by the induction of BMP antagonists by Nodal), but it is novel to put

© 2024. Published by The Company of Biologists under the terms of the Creative Commons Attribution License (https://creativecommons.org/licenses/by/4.0/).

them together to investigate axis extension using explants. The paper will be of interest to those interested in how these signaling pathways operate in early vertebrate development and to those interested in morphogenesis.

Reviewer #3 (Evidence, reproducibility and clarity (Required)): Summary:
The manuscript by Schauer et al. uses embryonic explants to study the coordination of Nodal and BMP signaling for embryo morphogenesis. They show that Nodal signaling triggers explant elongation by inducing mesendodermal progenitors that undergo cell intercalation. Looking at the role of BMP signaling, they show that BMP overactivation ventralizes the explants, reducing cell intercalation and therefore explant elongation.
Looking at pSmad5, they then establish that BMP signaling in the explant is attenuated by Nodal signaling, through activation of chordin expression, and through some unidentified chordin-independent mechanisms. Moving to the entire embryo, using combinations of BMP overexpression and Nodal inhibition, authors show that Nodal signaling limits BMP signaling on the dorsal side of the embryo, which is key to proper embryo elongation.

Major comments:
- The authors used the sebox::EGFP line to show that the growing region of the explant consists mostly of mesendodermal cells. Although this transgenic line had not been used to do so, the authors and others, had previously demonstrated that the extending part of the explant is mostly made of mesoderm and even shows some patterning (1,2). This should be stated and not presented as a new finding.
We thank the reviewer for raising this point. We have now changed the text (page 4/line 95-100) to more explicitly refer to the earlier work showing that the explant extension mainly consists of mesendoderm and to clarify that the novelty of the presented results lies in dissecting the contribution of different germ layer progenitor cell types to explant extension and cell rearrangement patterns.

- Explant elongation is driven by cell intercalation. The authors analyzed the shape of the mesendodermal tissue to conclude that cells intercalate. While I do not question this conclusion, as it is well known in the embryo, direct observation of cell intercalation, as was done in the embryo (3), would be a better demonstration.
We would like to argue that our conclusions are not solely based on the analysis of the morphology of the mesendodermal tissue, but also on the following features:
- elongation and narrowing of mesendodermal cell clones (Fig.1G-I, Fig.S1I,I'). Importantly, this analysis of the dispersal pattern was performed on individually tracked progenitors (see response to reviewer 1), and we have now additionally included movies of the cell tracks to better visualize single cell movements (Supplementary Video 2-4).
- polarization and medially-oriented alignment of mesendodermal progenitors perpendicular to the axis of explant elongation (Fig.1J,K, Fig.S1K).

To further address the cell intercalation behavior of mesendodermal progenitors, we have now included, as mentioned, additional movies of the tracked cell movement showing the corresponding cell tracks (Supplementary Video 2-4 for mesendodermal clones) as well as high magnification movies of the cell outlines which show examples of intercalation events (Supplementary Video 6).

- Explant elongation is driven by mesendodermal cell intercalation. I certainly agree from the movies and images that the extending region is mostly made of mesendoderm. However, it seemed clear to me that in Movie 1, starting at about 140 minutes, most of the convergence movement is taking place in a non-green region of the explant, fueling the extension of the mesendodermal region. Also, to demonstrate that cell intercalation is occurring in the mesendoderm, the authors performed clone dispersal analysis, comparing clones of mesendodermal and ectodermal cells. However, the selected ectodermal clone is very far from the extending region. To show that the cell intercalation is specific to mesendoderm, I think the authors should try to compare the behavior of mesendodermal and non-mesendodermal cells that are located at the same distance from the extending region. For example, from the image in Figure 1E (235 mpe), it appears that the right side of the base of the extended region is not green and could be compared to the left side. Currently, the quantification shown in 1G mostly demonstrates that the extending region is extending, and that the non-extending region is not.

© 2024. Published by The Company of Biologists under the terms of the Creative Commons Attribution License (https://creativecommons.org/licenses/by/4.0/).

We thank the reviewer for raising this very important point and would like to clarify that we do not want to claim that cell intercalation behavior is unique to the mesendoderm. As pointed out by the reviewer and in the original version of our manuscript, ectodermal cells also show a dispersal pattern indicative of cell intercalations, in line with previous studies (Williams & Solnica-Krezel, 2020); however, ectodermal cell rearrangements seem to be restricted to later stages of the explant elongation (Fig.1I). We acknowledge that this is not easily understood from the original text and have thus revised the text (note in particular page 5/line 141-147) to more explicitly mention this point and explain that, although the elongation movement seems to be initiated by mesendodermal progenitors (Fig.1C-H, Fig.S1D), the coordinated activity of ectodermal and mesendodermal C&E movements is most likely required to reach the full axis extension (see also Discussion page 16/ line 473-476). Moreover, we have revised the text throughout the manuscript to clarify that mesendoderm elongation during explant elongation is mediated by mesendodermal cell intercalations, but that the overall explant elongation process likely depends on coordinated mesendoderm and ectoderm progenitor cell rearrangements.
Moreover, and in line with the reviewer's suggestion, we have further analyzed the elongation of the ectoderm and mesendoderm by quantifying the change in the relative location of the boundary of the mesendoderm and ectoderm closest to the explant extension (Fig. S1F), circumventing potential biases due to clone location. This shows - similarly to the clone dispersal - that the mesendoderm elongates earlier and to a larger extent than the ectoderm (within the analyzed time-frame). These results are now mentioned on page 4/line112-116.

- Based on their observations in explants, the authors propose that Nodal signaling maintains an area of low BMP signaling on the dorsal side of the gastrula for robust axis elongation. While I acknowledge that the experiments performed by the authors have not been previolsy reported, I did not understand how this differs from the very well established fact that Nodal inhibits BMP signaling, in particular through chordin expression. Von der Hardt for instance already reported that overexpression of bmp and inhibition of chordin leads to severe elongation defects (4). More insight could probably be gained by analyzing the effect in more detail: Is the elongation defect due to cell intercalation defects? How are cell fates affected? Is this Nodal effect mediated by Chordin?...
We would like to argue that our experimental set up differs from the experiments reported by von der Hardt et al: the findings from the von der Hardt study provide evidence for increased BMP signaling leading to axis elongation defects, while our work shows the importance for Nodal signaling in restricting BMP signaling activity to allow proper axis elongation. We have revised the text to more clearly outline this difference in experimental design and interpretation. We also respectfully disagree with the notion that Nodal inhibiting BMP signaling is already a well-established fact: to the best of our knowledge reports to date have been limited to analyzing the effect of Nodal signaling on the expression of both negative regulators of BMP signaling, such as *chordin* (Gritsman et al., 1999; Sirotkin et al., 2000; Bennett et al., 2007; Varga et al., 2007), and positive regulators, such as *bmp4* and *admp* (Gritsman et al., 1999; Lele et al., 2001), making it difficult to clearly predict how changes in Nodal signaling levels affect BMP signaling activity as such. In fact, the only study as of yet, which actually addresses the effect of Nodal signaling on the pSmad5 gradient, has shown that BMP signaling activity is largely independent of Nodal signaling until the onset of gastrulation (Rogers et al., 2020). Our work also goes beyond just showing that BMP signaling activity is affected by changes in Nodal signaling levels, by demonstrating how high levels of Nodal signaling allow the embryo to buffer small increases of BMP signaling, thereby providing robustness to the axis elongation process.
This said, we do agree with the reviewer's assessment that important questions remain in the von der Hardt study as to the cellular basis of the observed axis elongation defects in BMP overexpressing embryos. In our present study, we have addressed this question by using explants as a simplified model of axis elongation that predominantly shows hallmarks of cell intercalation movements in the mesendoderm, rather than a combination of cell intercalation and convergent migration as found in the intact embryo. We show that cell polarization and alignment, prerequisites for medially-oriented intercalations (Glickman et al., 2003; Williams & Solnica-Krezel, 2020), are disrupted by overactivation of BMP signaling (Fig. 3C,D), a phenotype that is also detectable in the dorsal mesendoderm of intact embryos (Fig.S10C,D). To further dissect the relationship between BMP signaling levels and cell alignment under concomitant disruption of proper Nodal and BMP signaling, we have now shown that increased levels of pSmad5 coincided with a loss of mesendoderm cell alignment, and that upon overexpression of the highest tested *bmp2b* concentration (5 pg) with concomitant reduction of Nodal signaling (1 μM SB-505124), even

© 2024. Published by The Company of Biologists under the terms of the Creative Commons Attribution License (https://creativecommons.org/licenses/by/4.0/).

cells which do not show a clearly recognizable increase in pSmad5 activity became less polarized. This suggests a complex interplay between Nodal- and BMP-sensitive effectors and/or long-range regulation by BMP signaling in disrupting cell alignment, which will be of interest for more detailed analysis in future studies. These results are shown in Fig.S10E-H'' and on page 13-14/line 382- 397.

Minor comments:

- Fig6B. Are the curves significantly different? If so, how were they compared?

Please find in the figure below the p values following statistical comparison in the individual bins across the embryo axis (Reviewer figure 1). We would also like to point to the graphs showing the raw data for this experiment (Fig. S8C-E), as well as the frequency distribution plots, which show the comparative expansion of high pSmad5 nuclear intensity domain towards the dorsal side in MZ*oep* mutant embryos (Fig. S8F- F'''). Moreover, we now verified this expansion of the pSmad5 domain upon loss of Nodal signaling by treating embryos with the Nodal inhibitor SB-505124 (Fig. S8G-J).

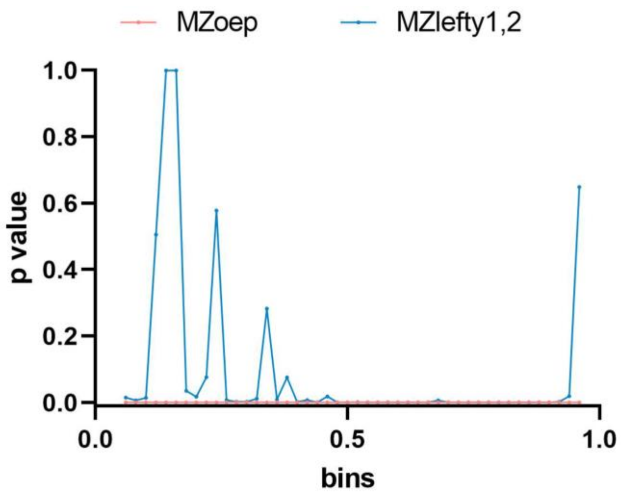

Reviewer figure 1. P-values determined by Kruskal-Wallis test calculated for the distribution of pSmad5/DAPI intensities in 0.02-wide bins from the ventral towards the dorsal side between MZ*oep* and wildtype and MZ*lefty1,2* and wildtype embryos, respectively. The data correspond to Fig.6B.

- Fig6D-E, I found the quantification a bit confusing. The reader is left with the impression of an all-or-nothing answer (effect only with BMP overexpression and strong Nodal inhibition), whereas the effect on the pSmad5 gradient is gradual. Plotting and comparing the pSmad5 intensity gradients would be better.

Thank you for pointing this out! We have now performed the analysis of the pSmad5 gradients, which can be found in Fig.6E, Fig.S9A-G'''.

- Fig6G. 'Axis length/embryo height' should appear on the x-axis, not the y-axis.

This has been changed.

Referees cross-commenting

I feel that the three reviews are very much in agreement, recognising that the experiments carried out are well done and calling for a reasonable amount of additional data. The three reviews also agree that the results obtained here in explants were already known from intact embryos, limiting the relevance to ex vivo research.

Reviewer #3 (Significance (Required)):

Overall, the experiments appear carefully carried out, and very precisely quantified. The paper is well written and easy to read. The results add to our understanding of the morphogenetic events occurring in embryonic explants. I therefore support their publication.

My main concern is with the significance of the results. I am convinced that embryonic explants are great tools, to reduce the complexity of the embryo and to address questions that cannot be

© 2024. Published by The Company of Biologists under the terms of the Creative Commons Attribution License (https://creativecommons.org/licenses/by/4.0/).

addressed in the embryo, as the authors and others have done, for instance, to address the role of extraembryonic tissues and patterning by maternal contributions. Here, however, I felt that most, if not all, of the experiments essentially demonstrate in embryonic explants, results that have been known for years in the intact embryo. While gathering detailed information on what happens in embryonic explants will certainly prove useful in further understanding the self- organizing abilities of these explants, and is worth publishing, the significance of the results reported here seems limited. Specifically, that elongation is driven by cell intercalation, that BMP-mediated dorsoventral patterning affects cell intercalation, that BMP signaling is attenuated by Nodal through Chordin, that Chordin is required for elongation, has been well established in the embryo over the last 20 years. Again, showing that it works the same way in embryonic explants is of interest, but at this point, does not add to our understanding of embryonic development.

While we fully acknowledge that some of the regulatory links we present in our study can be inferred from previous work, to which we have extensively referred throughout the manuscript, we also would like to argue that our results go clearly beyond previous work in several aspects as pointed out in our responses above. However, we also realize that we have failed to sufficiently convey some of the core messages and novelties of our work, applying to both explant and embryos, and have tried to now more clearly highlight those aspects by extensive revision of the text and new experiments. Specifically, we tried to emphasize and strengthen the following two points:

(i) The penetrance of the effect of Nodal signaling on the pSmad5 gradient is more pronounced in the explants than the intact embryo, with Nodal signaling leading to comparatively mild changes of the BMP signaling domain in the intact embryo during gastrulation, while being the key determinant of the pSmad5 domain extent in the explant. Given that germ layer patterning is largely determined downstream of maternal dorsal determinants in zebrafish explants (Schauer et al., 2020; Fulton et al., 2020), while a combination of maternal dorsal determinants and extraembryonic signals provided by the yolk syncytial layer are patterning the intact zebrafish embryo (reviewed in Marlow, 2020; Fuentes et al., 2020; Hill, 2022; Concha & Reig, 2022), our findings suggest that in the intact embryo the interplay of maternal determinants and extraembryonic signaling factors provides additional robustness to BMP gradient formation in absence of Nodal signaling. Vice versa, when extraembryonic signaling factors are absent, as in the explants, the BMP signaling gradient becomes more sensitive to variations in Nodal signaling. This context-dependency represents an interesting example where partially redundant processes (dorsal patterning and signaling from extraembryonic structures) provide robustness to allow key developmental programs to unfold relatively normally even in a perturbed environment (Wolpert, 1992). For a comparison of the sensitivity of the pSmad5 gradient to changes in Nodal signaling levels see page 12-13, line 355-365 and in Fig. S9H-I, and for a Discussion of this topic see page 15-16/line 452-466).

(ii) Both Nodal and BMP signaling have previously been implicated in the regulation of cell behavior during vertebrate gastrulation (reviewed in Zinski et al., 2018). Yet, how their combined morphogenetic activity domains are spatiotemporally correlated and integrated is only poorly understood. Our study shows that the Nodal-dependent capacity of cells to become polarized and align with the embryonic axis to undergo mediolateral cell intercalations is disrupted by ectopic BMP signaling. This provides insight not only into how the balance of Nodal and BMP signaling regulates the coordination of cell intercalations (mediated by Nodal signaling) and cell migration (mediated by BMP signaling), but also more generally, how such a developmental program is adapted to the specific morphology of the system, i.e. the requirement of convergent migration in the embryo with a large yolk cell, but not in explants lacking such a structure. To further dissect the relationship between Nodal and BMP signaling and cell alignment and polarization, we have now analyzed differences in pSmad5 levels together with cell alignment in embryos treated with low concentrations of Nodal inhibitor and overexpressing different concentrations of *bmp*. We found that reducing Nodal signaling drastically increases the sensitivity towards ectopic *bmp* regulating pSmad5 levels and controlling cell alignment. Interestingly, we also found that in embryos where pSmad5 levels are only very mildly or not at all upregulated, cell alignment can be affected when *bmp* was increased and Nodal signaling reduced, asking interesting questions as to the mechanisms and range of cell polarization regulation by combined Nodal and BMP signaling. These results are now included in Fig.S10E-H’’ and mentioned/discussed on page 13-14/line 383-397 and page 14-15/line 424-427.

© 2024. Published by The Company of Biologists under the terms of the Creative Commons Attribution License (https://creativecommons.org/licenses/by/4.0/).

Second decision letter

MS ID#: DEVELOP/2023/202316

MS TITLE: Robust axis elongation by Nodal-dependent restriction of BMP signaling

AUTHORS: Alexandra Schauer, Kornelija Pranjic-Ferscha, Robert Hauschild, and Carl-Philipp Heisenberg

I'm pleased to say that there are only a few minor comments from one of the reviewers for you to consider before we proceed to publication. It is your decision whether you wish to make further changes to the manuscript to address the comments made. The referees' comments are appended below, or you can access them online: please go to BenchPress and click on the 'Manuscripts with Decisions' queue in the Author Area.

Reviewer 1

Advance summary and potential significance to field

In this revised manuscript, the authors have addressed most of my concerns, either providing additional data or modifying the text. The point that doesn't fully convince me is the characterization of intercalation. The fact that measurements are made on cell trajectories does not in itself guarantee an analysis of cell behavior. In this case, the authors measure, for each cell, the distance to all other cells in the group (and not to neighbors). Thus, they measure the shape of the group of tracked cells. Any mechanism that causes a change in the shape of the group would lead to the same measurements. Nevertheless, I have no doubt that intercalation exists, and the authors now show some examples in a video. How much cell intercalation is responsible for the observed elongation, is, I think, not really analyzed.

Regarding significance, I do acknowledge that directly looking at the pSmad5 and pSmad2 gradients rather than at the expression of BMP regulators or target genes is new and raises some interesting questions (in particular the apparent discrepancy with previous results regarding the role of Nodal signaling in establishing the BMP gradient at the onset of gastrulation). However, as raised by the three reviewers, most of the molecular mechanisms reported in the explants were predicted by previous studies using intact embryos, and I am afraid I still don't fully understand how the experiments done here in explants contribute to understanding what happens in vivo.

Comments for the author

Minor point: In response to my question about the difference between the curves in Fig. 6B, the authors provided a graph of p-values obtained by performing Kruskal-Wallis tests on binned data. This may be correct, but it seems an odd way to compare distributions. As the authors point out, it would probably be better to compare the raw data (Fig. S8F), although I do not know how to do this properly (perhaps a two-dimensional KS test, like the Peacock test). I suppose it would be helpful to consult a statistician.

Reviewer 2

Advance summary and potential significance to field

The authors presented intriguing observations on the molecular mechanisms regulating morphogenic cell movement, with a particular focus on convergent-extension (CE) movement associated with cell type specification in the zebrafish blastoderm explant. In this manuscript, Schauer et al. identified the CE movement of the mesendoderm as triggering the elongation of the zebrafish embryonic explant. In this process, the Nodal signal represses the BMP signal, which negatively regulates the movement of the mesendoderm precursors, through the induction of its inhibitor chordin and noggin1. This suggests that the Nodal signal is the key factor coordinating cell fate specification and morphogenesis in the zebrafish blastoderm explant. Finally, suppression of

© 2024. Published by The Company of Biologists under the terms of the Creative Commons Attribution License (https://creativecommons.org/licenses/by/4.0/).

Nodal signalling increases sensitivity to BMP signalling in the CE movement of intact embryos. This suggests that promotion of mesendoderm cell intercalation via BMP suppression by Nodal may be involved in conferring robustness to morphogenic cell movement in vivo.

The zebrafish blastoderm explant assay has the potential to elucidate the molecular mechanisms regulating the complex processes of morphogenesis during vertebrate gastrulation, as the authors demonstrate in this paper. In this manuscript, the authors addressed the molecular mechanism coordinating cell fate specification and morphogenic cell movement in the blastoderm explant. All of the experiments are well-designed, the interpretation of the results is convincing and the paper is well-written. Also, the conclusion is clear and well supported by the presented data. These findings provide fundamental insights for studying morphogenic cell movements in early vertebrate embryos.

Comments for the author

The authors have satisfactorily addressed my previous comments, so I can now fully recommend publication in Development.

Reviewer 3

Advance summary and potential significance to field

This paper from the Heisenberg lab takes a reductionist approach to understanding how BMP and Nodal signaling interact to coordinate morphogenesis. They mostly use blastoderm explants that they culture in vitro. These explants elongate over time, with Nodal signaling that induces mesendoderm driving the cell intercalations that explain the elongation. They show that increased BMP signaling inhibits this process, but reducing BMP signaling has no effect. They see that reducing Nodal signaling results in an upregulation of BMP activity as read out by phosphorylated Smad5 staining and increasing Nodal signaling has the opposite effect. They explain this mostly by the observation that Nodal induces the expression of the BMP antagonist, Chordin, and validate this idea by demonstrating that a reduction in Chordin expression reduces explant elongation. Returning to the embryo, the authors show that manipulation of Nodal signaling levels influences the size of the BMP activity gradient as expected from the in vitro results. Finally, they show that reduction of Nodal signaling with SB505124 sensitises the embryos the effects of bmp2b overexpression, and that BMP overactivation at 90% epiboly reduced C&E movements.

The paper is well done and provides important information about the interactions between Nodal and BMP signaling to induce axis elongation. It will be of interest to those interested in how these signaling pathways operate in early vertebrate development and to those interested in morphogenesis.

Comments for the author

The authors have thoroughly revised their paper and in doing so have certainly improved it and have adequately addressed my previous comments and criticisms. I have no further questions.

Second revision

Author response to reviewers' comments

Dear Steve,
we would like to keep the manuscript as is. I think all the files we had submitted with the last version are fitting to the formatting guidelines. Please let me know if there is anything else I can do.

© 2024. Published by The Company of Biologists under the terms of the Creative Commons Attribution License (https://creativecommons.org/licenses/by/4.0/).

Third decision letter

MS ID#: DEVELOP/2023/202316

MS TITLE: Robust axis elongation by Nodal-dependent restriction of BMP signaling

AUTHORS: Alexandra Schauer, Kornelija Pranjic-Ferscha, Robert Hauschild, and Carl-Philipp Heisenberg
ARTICLE TYPE: Research Article

Thank you for sending your manuscript to Development through Review Commons.

I am happy to tell you that your manuscript has been accepted for publication in Development, pending our standard ethics checks.

© 2024. Published by The Company of Biologists under the terms of the Creative Commons Attribution License (https://creativecommons.org/licenses/by/4.0/).
